# Discovering Noncritical Organization: Statistical Mechanical, Information Theoretic, and Computational Views of Patterns in One-Dimensional Spin Systems

**DOI:** 10.3390/e24091282

**Published:** 2022-09-11

**Authors:** David P. Feldman, James P. Crutchfield

**Affiliations:** 1 College of the Atlantic, Bar Harbor, ME 04609, USA; 2 Department of Physics, University of California, Davis, CA 95616, USA

**Keywords:** spin systems, statistical mechanics, thermodynamics, information theory, computational mechanics, 05.50.+q, 64.60.Cn, 75.10.Hk

## Abstract

We compare and contrast three different, but complementary views of “structure” and “pattern” in spatial processes. For definiteness and analytical clarity, we apply all three approaches to the simplest class of spatial processes: one-dimensional Ising spin systems with finite-range interactions. These noncritical systems are well-suited for this study since the change in structure as a function of system parameters is more subtle than that found in critical systems where, at a phase transition, many observables diverge, thereby making the detection of change in structure obvious. This survey demonstrates that the measures of pattern from information theory and computational mechanics differ from known thermodynamic and statistical mechanical functions. Moreover, they capture important structural features that are otherwise missed. In particular, a type of mutual information called the *excess entropy*—an information theoretic measure of memory—serves to detect ordered, low entropy density patterns. It is superior in several respects to other functions used to probe structure, such as magnetization and structure factors. ϵ-Machines—the main objects of computational mechanics—are seen to be the most direct approach to revealing the (group and semigroup) symmetries possessed by the spatial patterns and to estimating the minimum amount of memory required to reproduce the configuration ensemble, a quantity known as the *statistical complexity*. Finally, we argue that the information theoretic and computational mechanical analyses of spatial patterns capture the intrinsic computational capabilities embedded in spin systems—how they store, transmit, and manipulate configurational information to produce spatial structure.

**Authors’ Note**: *What follows is a manuscript completed in 1998 (Santa Fe Institute Working Paper 98-04-026) and that, for various reasons, has not appeared in the peer-reviewed literature until now, although much of the material covered below was included in the first author’s PhD dissertation on which the second author was advisor [1]. We thank* Entropy *for the opportunity to publish it today. It appears in its original form, with only minor edits to improve clarity. We believe this overview of statistical mechanical, information theoretic, and computational mechanical approaches to discovering and quantifying patterns in one-dimensional spin systems is as relevant today as it was almost twenty-five years ago.*

*In the interim, we built on and extended the results via a number of publications, including Refs. [2,3,4,5,6,7,8,9,10]. A Python package for discrete information theory, allowing one to calculate many of the quantities discussed below, can be found at Ref. [11]. Overviews of related approaches to structural complexity include Refs. [12,13,14].*

## 1. Introduction

The questions we consider fall into three broad areas. First, what is a *pattern* [15]? An initial response might be that a pattern is some observed regularity or repeated tendency. Recall that for some time now there has been considerable interest in “pattern-forming” systems. However, what exactly does “pattern” mean in this setting? Who is to say what patterns are, and who determines which systems have generated patterns and which have not? Moreover, many natural patterns are only approximate. So how do we manage to separate pattern from mere noise? Presumably, we also have to consider the possibility that noise is part of the pattern. Is there some way to formalize what a noisy pattern is?

A second area of questions concern *organization*: what does it mean to say that a system is organized? In statistical mechanics, *order* is often associated with a broken symmetry. For example, the Ising model orders by acquiring a net magnetization when the spin-flip symmetry is broken. Can we define a similarly general notion of organization? Can we distinguish between different types of organization? There has been much effort expended recently to study “self-organizing” systems. However, who is to say which systems are organized and which are not? More to the point, where is the “self” in a self-organizing process? The organized-nonorganized distinction is very crude. Cannot there be degrees of organization? How would one say that one system is more organized than another?

A third set of questions revolves around information processing: how can we detect the computation being performed by a physical process or by other natural systems, such as the immune system or the visual cortex, in which pattern recognition, decision-making, and the like are (ostensibly) the central functions of the underlying dynamical behavior? In a condensed matter system, for example, how must spatial information be stored and shared so that the system can reach a critical state? How much historical memory is required to produce a given configuration? How do raw dynamical degrees of freedom support computation—the storage, transmission, and manipulation of information?

Are pattern, organization, and computation related in any way? Our hypothesis is that they are intimately related and that inquiring about a system’s computational capabilities is a concrete way to address questions of pattern and organization [16]. Computation, pattern, and organization are related in that they are all statements about the relationships within and between a system’s components and behavior. Restated in a more direct way, the hypothesis is simply that analyzing how a process “computes”—stores historical information, transmits information between internal degrees of freedom, and uses information to produce future behavior and system configurations—reveals how it is organized and what types of patterns it generates.

Establishing this hypothesis requires that we adopt a particular stance. Throughout this work the central issue is *discovering*, as opposed to *verifying*, pattern. The verification that a configuration displays one of a certain, a priori selected set of symmetries is not at issue here; though it is, admittedly, an important concern. Rather, the goal is to determine what organization is and which *kind* of patterns are intrinsic to a process. We ask, What in a process’s configurations and temporal behavior indicates how it is organized?

Faced with analyzing a system of many components, it is usually necessary to resort to a statistical description of some sort. A statistical analysis also becomes necessary when considering the trajectories followed by a chaotic dynamical system. To capture essential aspects of such systems, statistical analyses typically entail calculating some average property: temperature, compressibility, Lyapunov exponents, escape rates, and so on. However, these are certainly not the only quantities about which one can ask. The following considers some of the more detailed, yet still statistical, quantities that one can measure in a many-body setting and that indicate a process’s degree of organization.

Statistical mechanics has a very limited set of tools for discovering and quantifying structure, pattern, information processing, and memory in physical systems. It is our contention that to satisfactorily address these issues some tools must be added to the statistical physicist’s tool-box. The following reviews and adapts techniques and concepts from information and computation theories that will enable us to address questions of memory, structure, organization, and pattern. We apply these techniques to simple statistical mechanical systems to show that a richer set of tools is available for discovering pattern and describing organization in many-body systems.

### 1.1. Historical Context

Historically, the issues of pattern and organization have been the province of spatially-extended many-body systems, as analyzed by phase transition theory, to mention one approach. More recently, though, many of the same questions have arisen in the conundrum of deterministic chaotic dynamical systems: simple, but nonlinear processes produce unpredictable, seemingly random behavior. Physics has long possessed a measure of the uncertainty or randomness associated with a system—namely, the Shannon entropy [17,18] of the underlying distribution. The Shannon entropy, introduced over 100 years ago by Boltzmann, was adapted in the 1950s by Kolmogorov [19] and Sinai [20] to the study of dynamical systems. This, in turn, formed the foundation for the statistical analysis of deterministic sources of apparent randomness in the late 1960s through the early 1980s. These efforts to describe the randomness of a dynamical system have been rather successful. The metric entropy, Lyapunov exponents, and fractal dimensions form a widely applicable set of tools for detecting and quantifying unpredictable behavior; see, e.g., Refs. [21,22].

Since this time, however, it has become more broadly understood that a system’s randomness and unpredictability fail to capture its patterns and correlational structure. This realization has led to a considerable effort to develop a general measure or set of measures that quantify the structure of a system and the patterns it generates [16,23,24,25,26,27,28,29,30,31,32,33,34,35]. These quantities are often referred to as “complexity” measures. More properly, they should be called “structural complexity” or “statistical complexity” measures to distinguish them from Kolmogorov–Chaitin complexity [36], a measure of randomness, and computational complexity [37], a measure of resource (run time or storage) requirements in the theory of algorithms.

The following considers two approaches to measuring structure. First, we will see how information theory provides a measure of the memory stored in a system’s configurations. To date, this and related quantities have been estimated for the symbolic dynamics of chaotic dynamical systems [26,27,29,38], cellular automata [24,31], stochastic automata [32], spin systems [39,40], and hidden Markov models [16,41].

Second, we will examine how the architectural analysis of information processing provided by computation theory can be used to describe structure more completely than by using information theory or, for that matter, statistical mechanics. By using a hierarchical approach that begins with the least computationally powerful model classes, it is possible to infer the computation being performed by the system. This approach, an extension of statistical mechanics that includes elements of statistical inference and computation theory, we call *computational mechanics*.

For a more detailed discussion of the motivations and central issues that underlie computational mechanics, the reader is referred to Refs. [15,16,42]. Computational mechanics has been applied to the period-doubling and quasiperiodic routes to chaos [25,43], the dripping faucet [44], one-dimensional cellular automata [45,46], globally coupled maps [47], recurrent hidden Markov models [16,41], and stochastic resonance [48]. Computational mechanics has also been proposed [49] as a useful tool with which to re-examine the learning paradox of developmental psychology that concerns the discovery of new patterns, not seen before [15].

### 1.2. Focus

The following uses one-dimensional Ising systems to compare statistical mechanical, information theoretic, and computational mechanical views of structure, organization, and pattern. Since one-dimensional systems are generally considered simple, well-understood, and thoroughly analyzed, the contrasts between the statistical mechanical view and the structural view we take are particularly apparent.

Finite-range one-dimensional spin systems do not exhibit continuous phase transitions. As we will see, however, this does not mean that they are featureless systems, void of correlations and patterns. Indeed, a system need not be critical to be organized.

### 1.3. Applications

There are three overlapping areas of application of the tools for discovering and quantifying pattern, computation, and organization we develop here. First, the methods should be of benefit when considering small-scale physical systems as the basis of useful information-processing devices [50]. Along the same line, the information theoretic approach to memory might help clarify issues surrounding the “memory” observed in systems with charge density waves [51] or with glassy dynamics [52].

Second, it is likely that the structures that emerge in the canonical models of many-body systems (e.g., with Ising, XY, and Heisenberg Hamiltonians) can be analyzed more thoroughly through the use of computational mechanics and information theory. These model systems have formed the basis for much of our understanding of critical phenomena. Thus, it seems natural to reexamine these models by applying the computational and information theoretic apparatus discussed here.

Third, statistical mechanical techniques are now being applied to a wide range of nontraditional systems, such as self-organized criticality [53], genetic algorithms [54,55], traffic flow [56,57], and learning dynamics in neural networks [58,59]. Extant quantities in statistical mechanics have been influenced by the observability constraints of physical experiment. For the most part, only directly measurable quantities such as the pressure, conductivity, or net magnetization have been thoroughly developed. However, for some of these non-traditional systems such measurability constraints may not be limitations, since the microstates themselves can be directly observed. In these cases, one need not carry forward the traditional constraints, especially when new structural questions require different quantities to be estimated.

Information theory and computational mechanics provide a richer set of tools for studying these kinds of systems. Of course, the most revealing and meaningful quantities will always depend on the specific features of the system under study. It is not our intention to argue for *one* particular way to measure organization or pattern. Rather, we suggest that to fully capture patterns and organization in a wide range of many-body systems, the probes offered by statistical mechanics fall short; concepts and methods from information and computation theories become necessary.

### 1.4. Overview

The presentation is organized into three layers: introductory comments, definitions and reviews, and applications to 1D-spin systems. In each, we compare and contrast the statistical mechanical, information theoretic, and computational mechanical views of structure.

In Section 2 we review the basic statistical mechanical approaches to detecting and quantifying features in many-body systems. We also use this section to fix the notation and context that we will assume for the rest of the development. Researchers in statistical physics can skip to Section 2.3. Section 3 reviews information theory and defines the three key quantities: the Shannon entropy, the Shannon entropy rate, and a form of mutual information called the excess entropy. Beyond acquainting themselves with excess entropy, those conversant with coding and information theories or, say, symbolic dynamics may wish to skim this material. Section 4 then gives a concise, but self-contained, review of computational mechanics. Those familiar with previous reviews, such as Ref. [16], can skip this section.

In each of these three review sections, we begin by considering the central questions that motivate each approach and we shall see how the quantities introduced arise as natural answers to these questions. Awareness of the different motivating issues is crucial to understanding the differences and similarities between the three views of organization and pattern.

In Section 5 we report the results of applying the measures of structure to finite-range one-dimensional spin systems. We compare, for example, the excess entropy with the structure factors of statistical mechanics in Section 6. We will see that the excess entropy is capable of detecting periodic structure of any periodicity and thus may be viewed as an “all-purpose order parameter” for periodic patterns. In Section 7 we show that ϵ-machines are necessary to describe the structure of entropic patterns that do not have a strong periodic component. In so doing, we illustrate how an ϵ-machine provides an irreducible representation of an approximate symmetry. In Section 8 we directly compare the excess entropy with a number of commonly used measures in statistical mechanics: the correlation length, specific heat, ferromagnetic structure factors, and the nearest-neighbor correlation function. We argue that while there are qualitative similarities between all these functions, none can be viewed as a measure of memory in the sense that the excess entropy can be. Furthermore, we find that all these functions are maximized at different parameter values, indicating that they are not trivially related and that the statistical mechanical functions cannot be used to determine the parameter values at which a system’s spatial memory is maximized. Lastly, in Section 9 we summarize our comparisons and discuss directions for future work.

## 2. Statistical Mechanics

A central concern of equilibrium statistical mechanics is determining how physically-observable, bulk quantities can be explained from the behavior of the system’s constituents. For example, how are the conductivity, heat capacity, and compressibility of a metal determined by the interactions between the electrons and nuclei in the metal?

The starting point for such calculations is a knowledge of the microphysics—typically, the Hamiltonian for the system expressed as a sum or an integral over the system’s internal degrees of freedom. The connection between the energy determined by the Hamiltonian and the joint probability over the internal degrees of freedom is given by

(1) $$\mathrm{Pr}\left(C\right)\;\propto \;e^{-\beta H(C)}\;,$$

where C is a configuration of the system and H is the system’s Hamiltonian. The quantity $\beta =1/(k_{B}T)$ is the inverse temperature and $k_{B}$ is Boltzmann’s constant. For the remainder we set $k_{B}$ equal to one and measure the temperature *T* in dimensionless units.

In principle, given a Hamiltonian one can use Equation (1) to calculate macroscopically observable average quantities. However, performing the necessary sums is usually prohibitively difficult; a consideration we shall return to at the end of this section.

Thus, although Equation (1) sets out one (basic) approach to determining a system’s physical properties, the failure of its direct implementation leaves many questions unanswered. Not the least of which is the one that concerns us here: How does statistical mechanics go about discovering and quantifying structure? Before we begin addressing these questions, we pause to establish some notation and set the context for the following development.

### 2.1. Spin Systems: Notation and Definitions

The main object of our attention will be a one-dimensional chain $\overset{↔}{S}\equiv \ldots S_{-2}S_{-1}S_{0}S_{1}\ldots$ of spins (random variables) $S_{i}$ that range over a finite set A. For a spin-*K* system |A|=2K+1. Alternatively, one may also consider the chain as being a stationary time series of discrete measurements. We shall restrict ourselves to configuration distributions that are time independent. That is, we consider only equilibrium distributions. If we imposed some time dependence—say, a Glauber dynamics or an update rule for a one-dimensional cellular automaton—then we would need to include a time index on all the spin variables.

We divide the chain into two semi-infinite halves by choosing a site *i* as the dividing point. Denote the left half by

(2) $$\overset{\leftarrow }{S_{i}}\equiv \ldots S_{i-3}S_{i-2}S_{i-1}$$

and the right half by

(3) $$\vec{S_{i}}\equiv S_{i}S_{i+1}S_{i+2}S_{i+3}\ldots \;.$$

We will assume that a spin system is described by a spatial shift-invariant measure μ on bi-infinite configurations $\cdots s_{-2}s_{-1}s_{0}s_{1}s_{2}\cdots ;s_{i}\in A$. The measure μ induces a family of distributions that will be of primary interest. Let $\mathrm{Pr}\;(s_{i})$ denote the probability that the $i^{\mathrm{th}}$ random variable $S_{i}$ takes on the particular value $s_{i}\in A$ and $\mathrm{Pr}(s_{i+1},\ldots ,s_{i+L})$ the joint probability over blocks of *L* consecutive spins. Assuming spatial translation symmetry: $\mathrm{Pr}\left(s_{i+1},\ldots ,s_{i+L}\right)=\mathrm{Pr}\left(s_{1},\ldots ,s_{L}\right)$. We denote a block of *L* consecutive spin variables by $S^{L}\equiv S_{1}\ldots S_{L}$. We shall follow the convention that a capital letter refers to a random variable, while a lower case letter denotes a particular value of that variable. Therefore $s^{L}$ denotes a particular spin-block configuration of length *L*. Finally, in the following we shall use the term *process* to refer to the joint distribution over the bi-infinite chain of variables.

We now define the Hamiltonians we shall use to generate equilibrium distributions of our spin chain. A general Hamiltonian for a one-dimensional chain of *N* spins that interact in pairs is given by

(4) $$H\left(s^{N}\right)=-\sum_{i,j=1}^{N}J_{ij}s_{i}s_{j}-B\sum_{i=1}^{N}s_{i}\;,$$

where the $J_{ij}$s are parameters determining the strength of coupling between spins, *B* represents an external field, and $s_{i}\in \left\{+1,-1\right\}$ or $s_{i}\in \left\{↑,↓\right\}$, for example. Below, we shall consider only interactions within a finite range R<∞; that is,

(5) $$J_{ij}=0,\;\left|i-j\right|>R\;.$$

We shall also consider only coupling constants that are translationally invariant; i.e., those depending only on |i−j| and not *i* and *j* individually. Hence, we define:

(6) $$J_{r}=J_{ij}\;,$$

where r=|i−j|. Despite these restrictions on $J_{ij}$, the quantities discussed below are perfectly general and apply to any lattice system.

The canonical partition function for these spin systems is defined by

(7) $$Z=\sum_{\left\{s^{N}\right\}}e^{-\beta H(s^{N})}\;.$$

The sum is understood to extend over all ${\left|A\right|}^{N}$ possible configurations of length *N*.

The average internal energy *U* is simply the expectation value of the Hamiltonian and can be expressed as:

(8) $$U=-\frac{1}{N}\frac{\partial \ln Z}{\partial \beta }\;.$$

The free energy *F* per site is given by

(9) $$F=-\frac{T}{N}\log Z\;.$$

In the thermodynamic limit, in which the system size *N* goes to infinity, *Z* typically diverges exponentially so *F* remains finite.

The thermodynamic entropy is defined as the logarithm of the number of microstates accessible at a given energy. In the canonical ensemble, the entropy per site S is related to the free energy per site *F* via:

(10) $$S=-\frac{\partial F}{\partial T}\;.$$

Finally, the magnetization *m* per site is defined as the average:

(11) $$m\equiv \frac{1}{N}\langle \sum_{i=1}^{N}s_{i}\rangle \;,$$

where here and below angular brackets indicate thermal expectation value

(12) $$\langle •\rangle \;\equiv \;\frac{1}{Z}\sum_{\left\{s^{N}\right\}}\;•\;e^{-\beta H(s^{N})}\;.$$

### 2.2. Statistical Mechanical Measures of Structure

#### 2.2.1. Correlation Function and Correlation Length

With the above notational preliminaries out of the way, we consider our first measure of “structure”: the *two-spin correlation function* $\Gamma_{ij}$, defined in the usual way as

(13) $$\Gamma_{ij}\;\equiv \;\langle \left(s_{i}-\langle s_{i}\rangle \right)\left(s_{j}-\langle s_{j}\rangle \right)\rangle \;.$$

This quantity is sometimes called the truncated or connected correlation function to distinguish it from $\langle s_{i}s_{j}\rangle$. It follows from translation invariance that $\langle s_{i}\rangle =\langle s_{i+k}\rangle ,k=1,2,\ldots$. This enables us to write the correlation function as

(14) $$\Gamma_{ij}=\langle s_{i}s_{j}\rangle -{\langle s\rangle }^{2}\;,$$

where $\langle s\rangle \equiv \langle s_{j}\rangle$. Thus, $\Gamma_{ij}$ measures the tendency of the fluctuations (about the mean value) of spins at site *i* and at site *j* to be correlated with one another.

Again from translation invariance it follows that $\langle s_{i}s_{j}\rangle =\langle s_{i+k}s_{j+k}\rangle ,\;k=1,2,\ldots$. So, the correlation function depends only on r=|i−j| and not on *i* and *j* individually. This leads one to define:

(15) $$\Gamma \left(r\right)\;\equiv \;\langle s_{0}s_{r}\rangle -{\langle s\rangle }^{2}\;.$$

Except at a critical point, the correlations die exponentially with increasing *r*; that is,

(16) $$\Gamma \left(r\right)∼e^{-r/\xi }\;\;\mathrm{as}\;\;r\to \infty \;.$$

The quantity ξ is called the *correlation length*. Simply stated, it measures the range of influence of a single spin. Equivalently, ξ gives the size of a typical ordered cluster of spins. An infinite correlation length typically indicates that the correlation function dies algebraically, rather than exponentially. This occurs at the critical points of continuous (second or higher order) phase transitions.

#### 2.2.2. Susceptibility and Structure Factors

The *magnetic susceptibility* χ per site is defined as a measure of the system’s linear change dm in magnetization *m* per site due to the application of a small external field dB. That is,

(17) dm=χdB.

Thus,

(18) $$\chi =\frac{\partial m}{\partial B}=-\frac{\partial^{2}F}{{\left(\partial B\right)}^{2}}\;.$$

As is always the case with linear response functions, χ can be written as a sum of correlation functions

(19) $$\chi =\lim_{N\to \infty }\frac{\beta }{N}\sum_{i,j=1}^{N}\Gamma_{ij}\;.$$

We can exploit the translation invariance of $\Gamma_{ij}$ to perform one of the sums above. We then obtain:

(20) $$\chi =\beta \left[2\sum_{r=0}^{\infty }\Gamma \left(r\right)-\Gamma \left(0\right)\right]\;.$$

This expression for χ can be reconciled with its definition Equation (17) by realizing that, roughly speaking, the magnetization is more changeable with a variation in field dB the greater the correlations between spin pairs.

Equation (19) tells us that χ is a sum over correlation functions and as such might serve as a global measure of structure. In particular, consider the term $\sum_{r=0}^{\infty }\Gamma \left(r\right)$, the sum over all possible two-spin correlation functions, from Equation (20). At first blush, this seems to be an ideal quantity to use as an indicator of structure. By summing over all two-spin correlation functions χ appears to provide a measure of the total correlation across the lattice.

However, this turns out not to be the case. To see this, consider a system near an antiferromagnetic-paramagnetic transition. Clusters of ordered spins appear at all length scales, but the type of order within a cluster is antiferromagnetic—alternating up and down spins. Thus, the correlation functions Γ(r) for such a system will alternate in sign with *r* and will tend to cancel out when summed in Equation (20), resulting in a small quantity despite the presence of a strong antiferromagnetic ordering. To compensate for this, one could choose, for example, to multiply each term in the sum by ${\left(-1\right)}^{r}$. However, this is a somewhat arbitrary adaptation to a particular set of spin couplings that derives ultimately from our own appreciation of the underlying order.

Instead, we can take the Fourier transform of spin configurations. The result is a function that is usually called the *structure factor*. It is given by

(21) $$S\left(q\right)\equiv \sum_{r=0}^{\infty }e^{irq}\Gamma \left(r\right)\;.$$

The structure factor provides a measure of the correlation with a particular spatial periodicity, as measured by the wavenumber *q*. As an observable, S(q) is important for both simulation and laboratory experiments. In a simulation it is often S(q) that is calculated to look for a phase transition: an S(q) that diverges as a function of system size is a clear indication of critical behavior. In the laboratory, order in a magnetic system is often probed by means of neutron scattering. Assuming dipole interactions and fixed target spins, the probability for scattering to occur with a momentum transfer *q* is proportional to S(q); see, e.g., [60]. Neutron scattering is used, for example, to distinguish between a paramagnet and an antiferromagnet. Both types of materials have zero magnetization, but their magnetic structural properties are distinct.

Any transform (integral or discrete) carries with it representational restrictions that are implicit in its choice of function basis. For example, S(q), as with all Fourier analysis, carries an assumption that the underlying order is a linear superposition of periodic configurations. Hence, as we shall see, S(q) is not suited to detect aperiodicity. Moreover, it is sometimes the case that a particular choice of function basis results in an unnecessarily “large” description; for example, a Fourier decomposition of a square wave yields an infinite number of nonzero amplitudes.

Unfortunately, there is no universally accepted way to define a structure factor. One alternative is to define

(22) $${\tilde{S}}_{1}\left(q\right)\;\equiv \;\beta S\left(q\right)\;.$$

so that the susceptibility is more closely related to the structure factor: $\chi =2{\tilde{S}}_{1}\left(0\right)-\beta \Gamma \left(0\right)$. Another alternative is to argue that if the structure factor is to measure correlation between spins, the “self-correlation” term Γ(0) should be excluded from the sum; yielding

(23) $${\tilde{S}}_{2}\left(q\right)\;\equiv \;\sum_{r=1}^{\infty }e^{iqr}\Gamma \left(r\right)\;.$$

Both modifications of the structure factor do not significantly alter the features of its behavior reported below. As such, we shall focus our attention on S(q) as defined in Equation (21).

#### 2.2.3. Specific Heat

We conclude this brief review of statistical mechanical measures of structure by commenting on the specific heat. The specific heat *C* is a linear response function defined by

(24) dU=CdT,

where *U* is the internal energy. Like χ, *C* can be related to fluctuations—in this case, energy fluctuations:

(25) $$C=\beta^{2}\langle {\left(U-\langle U\rangle \right)}^{2}\rangle \;.$$

As a result, *C* measures fluctuations in energy, not in correlations between spins. To see this, consider a paramagnet, a spin system in which there are no couplings between the spins and so the spin variables are independently distributed. The specific heat for such a system is nonzero, reaching a maximum in the T≈B region. That *C* is nonzero for this system—a clearly correlationless paramagnet—indicates that C>0 is at best a misleading measure of spatial structure. Thus, the only structure reflected by the specific heat is local and not spatial: that in the bias of individual spins in the direction of the external field.

### 2.3. Other Statistical Mechanical Approaches to Structure

In the previous section we reviewed some basic quantities often used in statistical mechanics to detect and measure the presence of correlational structure. However, there are other, more subtle ways in which the search for structure enters into statistical mechanics than in the use of its typical observables.

A calculation of (say) the partition function by explicitly considering all allowed configurations is infeasible for all but the smallest of systems. It is quite often the case, however, that the probability distribution to be summed over has symmetries or internal structure that render large portions of the sum in Equation (7) redundant. Thus, one central challenge of statistical mechanics is to find these symmetries and figure out how to best exploit them.

As a simple example of the discovery and exploitation of symmetries, consider again the paramagnet. Since the spins do not interact, the energy of the system depends only on how many spins are up, say. Equivalently, the probability distribution of a single spin is independent of the others. Due to this particularly simple symmetry in the joint probability distribution over spin configurations, thermodynamic averages may be calculated by using the binomial theorem, rather than a brute force enumeration of all possible configurations.

A less trivial example of the “covert” role of structure in statistical mechanics is found in the technique of transfer matrices. For one-dimensional systems with finite-range interactions, such as the one-dimensional Ising models considered here, the partition function can be re-expressed in terms of the dominant eigenvalue of this finite-dimensional matrix. Moreover, the joint probabilities over spin configurations follow from the dominant left and right eigenvectors. Hence, all thermodynamic averages can be determined given knowledge of the transfer matrix. Loosely speaking, the transfer matrix encodes all of the information about the system. In subsequent sections we shall discuss transfer matrix methods in more detail.

Unfortunately, the transfer matrix method does not always work, often failing for systems with disorder or long-range interactions. It is only successful for systems whose joint probability distribution over configurations factors in a certain way: namely, the distribution over the spin chain must decompose into independent distributions over contiguous spin blocks of finite size. Said another way, if we imagine moving spatially along the chain, the stationary stochastic process generating the spins we observe must be a finite-memory Markov process.

When the transfer matrix method fails, sometimes it is possible to use an infinite dimensional matrix, i.e., an operator [61]. Another approach is the diagrammatic perturbation expansions of statistical field theory where one or several fundamental interactions are identified and their contributions to the thermodynamic quantities in question are summed up by considering more and more complicated interactions [60,62].

Yet another approach to finding and utilizing structure in the joint probability distributions over configurations relies on cycle expansion methods [63,64]. Here, one systematically approximates the partition function by considering the contributions from fundamental periodic configurations of successively longer periods. A particularly effective application of the cycle expansion technique is the calculation of the Lyapunov exponent of a product of random matrices [65].

The vantage point afforded by this brief overview suggests that one classify statistical mechanical systems by the type of mathematical entity—contiguous blocks, operators, fundamental interactions, cycles—needed to most efficiently “encode” their configurations. Then calculations of thermal averages can be performed most efficiently. In Section 4 we shall see that the ϵ-machines of computational mechanics provide the most general formalization of this idea.

## 3. Information Theory

To appreciate the interpretation and use of information-theoretic concepts in the comparisons that we develop in the following, an historical review is helpful. This will be, of necessity, brief. The interested reader is strongly advised to read basic reference works such as Ref. [17] or [18]. Those familiar with information theory may wish to skip to Section 3.2.

In the late 1940s Shannon founded the field of communication theory [17], motivated in part by his work in cryptography during World War II. This led to a study of how signals could be compressed and transmitted efficiently and error free. His basic conception was that of a *communication channel* consisting of an *information source* that produces messages which are encoded and passed through the channel. A receiver then decodes the channel’s output in order to recover the original messages. Key to his analysis was the definition of the source’s rate of information production, called the *source entropy rate*, and the maximum carrying capacity, called the *channel capacity*, of the (possibly noisy and error-prone) channel.

Much earlier, Hartley had proposed to measure the amount of information from a source via the logarithm of the number of possible source messages [66]. Shannon’s definition of the source entropy adapted Hartley’s measure to account for probabilistic structure in the source: some messages being more or less likely than others. He interpreted the negative logarithm of a message’s probability as a measure of surprise: the more unlikely a message the more informative it was, when it appeared. This surprise, averaged over a source’s messages, is the source’s entropy rate. The functional form of Shannon’s entropy, as he realized, had already been developed by Boltzmann in late 1800s as a measure of disorder of thermodynamic systems [67]. In the following we will refer to this and related quantities as Shannon entropy, however, since it will be used in the sense intended by information theory.

It is important to emphasize that the core of information theory concerns not so much the various definitions of information and entropy, but rather the relationship between the source entropy rates that can be sustained through channels and those channels’ capacities. These connections are what makes the similarity between Boltzmann’s notion of thermodynamic entropy and Shannon’s entropy rate so notable. Boltzmann clearly did not anticipate Shannon’s use of entropy.

The primary results on which information theory is built and with which it finds its technological applications are Shannon’s two central coding theorems. The first theorem says that information cannot be transmitted error-free through a channel at a rate higher the channel’s capacity. The second theorem says that as long as the source’s rate respects this limit then there exists an encoding and decoding scheme for the source’s messages such that error-free transmission is possible and can occur at rates arbitrarily close to the channel capacity.

The mathematical foundations of Shannon’s communication theory followed quickly [68,69], as did a number of applications and important extensions. For example, Jaynes re-introduced portions of information theory back into statistical mechanics, reformulating ensembles in terms of a maximum (Shannon) entropy assumption under various constraints [70]. This was partly motivated as an attempt to understand the role of probability in statistical mechanics and the similarities between statistical mechanics and statistical inference [71]. For a readable introduction to this approach to statistical mechanics, see Ref. [72]; a more thorough account can be found in Ref. [73].

The basic quantities used in information theory are various forms of Shannon entropy: the entropy *H* of a distribution, the information gain D of one distribution with respect to another, and the mutual information *I* between two distributions. When adapted and applied to different communication problems, these are the quantities in which the results of the theory are expressed. It is noteworthy that many uses of information theory in statistical physics and in nonlinear dynamics mostly employ its basic quantities and do not use the more characteristic and central aspects of coding.

### 3.1. Shannon Entropy: Its Forms and Uses

Consider a discrete random variable *X* that assumes values x∈A. The *Shannon entropy* H[X] of *X* is defined by:

(26) $$H\left[X\right]\equiv -\sum_{x\in A}\mathrm{Pr}\left(x\right)\log_{2}\mathrm{Pr}\left(x\right).$$

Note that H[X] is a function not of *x* but of the *distribution* Pr(X) of *X*. H[X] may be interpreted as the unique (up to a multiplicative constant) additive measure of uncertainty associated with a random variable *X*; see App. 2 of Ref. [17]. If the information source produces messages that are independent samples of *X* distributed according to Pr(X), then the average number of yes-no questions needed to determine a particular value *x* is between H[X] and H[X]+1. The unit of information that answers a single yes-no question is called a *bit*. This result is consonant with the interpretation of entropy as uncertainty. The more uncertain we are about an event, the larger the number of questions on average needed to ascertain the outcome [17,18]. Note that if Pr(X)=U(X), the uniform distribution over x∈X, then $H\left[X\right]=\log_{2}\left|A\right|$.

The Shannon entropy of source *X* measures the average uncertainty of observing outcomes *x* if we expect the outcomes to occur with probability P(x). However, what if, despite the actual events occurring according to P(X), we have prior knowledge that leads us to expect the outcomes are distributed with probability Q(x)? The relative information obtained in observing *X* is then given by the *information gain* D(P|Q):

(27) $$D\left(P|Q\right)\equiv \sum_{x\in A:Q(x)>0}P\left(x\right)\log_{2}\left[P\left(x\right)/Q\left(x\right)\right]\;,$$

where we assume that if Q(x)=0, then P(x)=0. The information gain is also known as the Kullback–Leibler divergence and the relative entropy. The information gain D is sometimes referred to as a distance, but it is neither symmetric in *P* and *Q* nor does it obey a triangle inequality. It is, however, nonnegative, and is zero only when the two distributions are equal. D(P|Q) is, in a sense, the number of bits it takes to change distribution *P* into *Q*. Note that $D\left(P|U\right)=\log_{2}\left|A\right|-H\left[P\right]$.

It is possible to define joint and conditional entropies [17,18]. Consider two discrete random variables, *X* and *Y*, that assume values $x\in A_{X}$ and $y\in A_{Y}$, respectively. Denote by Pr(x|y) the conditional probability that X=x given that Y=y. The *conditional entropy* of *X* conditioned on *Y* is defined by:

(28) $$H\left[X|Y\right]\equiv -\sum_{x\in A_{X},\;y\in A_{Y}}\mathrm{Pr}\left(x,y\right)\log_{2}\mathrm{Pr}\left(x|y\right)\;.$$

It measures the average uncertainty in the conditional distribution Pr(x|y). Note that H[X|Y] is not symmetric in *X* and *Y*.

The *joint entropy* is defined by

(29) $$H\left[X,Y\right]\equiv -\sum_{x\in A_{X},\;y\in A_{Y}}\mathrm{Pr}\left(x,y\right)\log_{2}\mathrm{Pr}\left(x,y\right)$$

and measures the average uncertainty associated with the joint distribution Pr(x,y).

The *mutual information* I[X;Y] between two random variables *X* and *Y* is defined by [17,18]:

(30) $$I\left[X;Y\right]\equiv \sum_{x\in A_{X},\;y\in A_{Y}}\mathrm{Pr}\left(x,y\right)\log_{2}\frac{\mathrm{Pr}(x,y)}{\mathrm{Pr}(x)\mathrm{Pr}(y)}\;.$$

The mutual information can be rewritten as the difference between a marginal and a conditional entropy:

(31) I[X;Y]=H[X]−H[X|Y].

In other words, the mutual information measures the reduction in the uncertainty of *X* given knowledge of *Y*. If the uncertainty of *X* is reduced, then we say that *Y* carries information about *X*. This is why *I* is known as the mutual information.

There are a number of basic properties of mutual information. First, it is nonnegative: I≥0. Second, *I* is symmetric in *X* and *Y*; I[X;Y]=I[Y;X]. Third, for both independent variables and zero-entropy variables, I=0.

One of the main uses of mutual information is in the definition of a channel’s information carrying capacity. If the source is denoted *X* and the output of the channel is denoted *Y*, then the channel capacity C is defined as

(32) $$C=\underset{\left\{X\right\}}{\sup}I\left[X;Y\right]\;,$$

where the supremum is taken over all information sources.

### 3.2. Entropy Growth

We now shift the emphasis back to analyzing spin configurations. Consider again the bi-infinite sequence $\ldots S_{-2}S_{-1}S_{0}S_{1}S_{2}\ldots$. The average uncertainty of observing an *L*-spin block $S^{L}$ is given by the Shannon entropy of the joint distribution $\mathrm{Pr}(s^{L})$ [18]:

(33) $$H\left(L\right)\equiv -\sum_{s^{L}\in A^{L}}\mathrm{Pr}\left(s^{L}\right)\log_{2}\mathrm{Pr}\left(s^{L}\right)\;.$$

**Figure 1:**
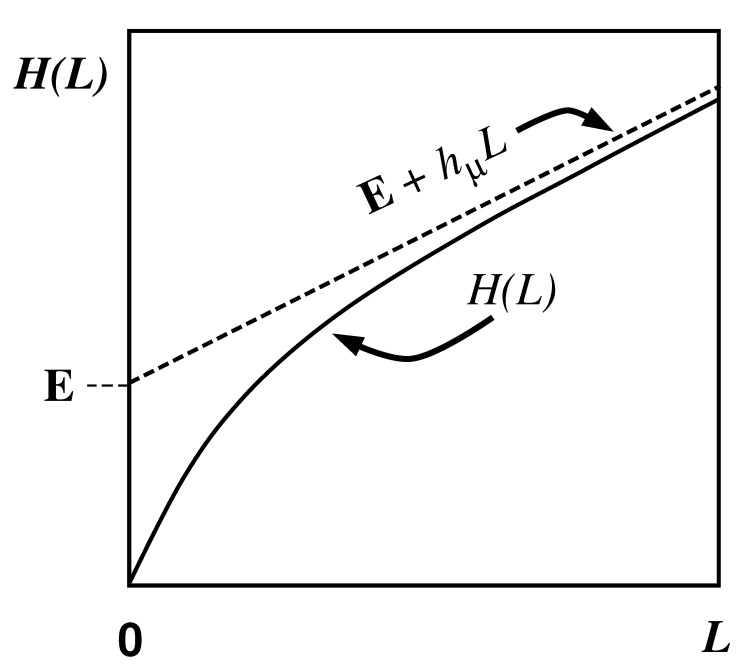
Total Shannon entropy growth for a typical information source: a schematic plot of H(L) versus *L*. H(L) increases monotonically and asymptotes to the line $E+h_{\mu }L$, where E is the excess entropy and $h_{\mu }$ is the source entropy rate.

We define H(0)≡0 and, for later use, H(L)=0,L<0. The block entropy is nonnegative, H(L)≥0, and monotonic in *L*; H(L)≤H(L+1). That is, adding an additional random variable cannot reduce uncertainty [18]. A schematic plot of H(L) versus *L* is shown in Figure 1 for a typical information source.

### 3.3. Entropy Density and Convergence to It

The spatial density of the Shannon entropy of the spin configurations is defined by

(34) $$h_{\mu }\equiv \lim_{L\to \infty }\frac{H(L)}{L}\;,$$

where μ denotes the measure over bi-infinite configurations that induces the *L*-block joint distribution $\mathrm{Pr}(S^{L})$. The quantity $h_{\mu }$ measures the irreducible randomness in spatial configurations: the randomness that remains after the correlations and structures in larger and larger spin blocks are taken into account. For physical systems $h_{\mu }$ is equivalent to thermodynamic entropy density—S in Equation (10)—in units where $k_{B}/\log_{e}2=1$. The entropy density is also known as the entropy rate or the metric entropy, depending on the application context.

The entropy density $h_{\mu }$ can be re-expressed as:

(35) $$h_{\mu }=\lim_{L\to \infty }\left[H(L+1)-H(L)\right]\;.$$

Thus, we see that the curve’s slope as L→∞ in Figure 1 corresponds to the entropy density $h_{\mu }$.

Equation (35) can also be rewritten by using the conditional entropy as defined in Equation (28) [18]:

(36) $$\begin{array}{ccc} h_{\mu } & = & \lim_{L\to \infty }H\left[S^{L}|S^{L-1}\right]\\ & = & \lim_{L\to \infty }H\left[S_{L},S_{L-1},\cdots ,S_{1}|S_{L-1},S_{L-2},\cdots ,S_{1}\right]\\ & = & \lim_{L\to \infty }H\left[S_{L}|S_{1}\ldots S_{L-1}\right]\;. \end{array}$$

Thus, $h_{\mu }$ is the uncertainty of the next spin value $s_{L}$ conditioned on the first L−1 spins in the *L*-block, as L→∞. This reinforces the interpretation of $h_{\mu }$ as the irreducible randomness associated with the system. Equation (36) indicates that $h_{\mu }$ measures our uncertainty about the variable $S_{L}$ given knowledge of *all* the spins that preceded it. In this sense $h_{\mu }$ measures, in units of bits per site, the per-spin unpredictability of the infinite string. Note that the entropy density is nonnegative: $h_{\mu }\geq 0$.

Equations (34)–(36) give different expressions for the entropy density $h_{\mu }$. These are all equivalent in the present setting, though they need not be for nonequilibrium or nonstationary processes [74].

The entropy density is a property of the system as a whole; only in special cases will the isolated-spin uncertainty H(1) be equal to $h_{\mu }$. This leads us to consider how random the spin chain appears when finite-length spin blocks are considered. This is given by:

(37) $$h_{\mu }\left(L\right)\equiv \;H\left(L\right)-H\left(L-1\right),\;\;L=1,2,\ldots \;,$$

the incremental increase in uncertainty in going from (L−1)-blocks to *L*-blocks. Thus, since we imposed the “boundary condition” H(0)=0, we have $h_{\mu }\left(1\right)=H\left(1\right)$.

**Figure 2:**
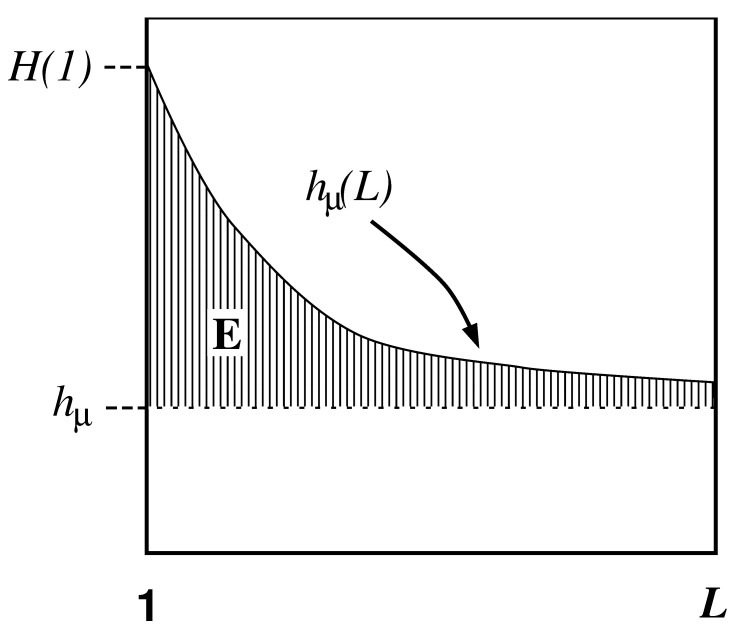
Entropy density convergence: A schematic plot of $h_{\mu }\left(L\right)$ versus *L* using the typical H(L) shown in Figure 1. The entropy density asymptote $h_{\mu }$ is indicated by the horizontal dashed line. The shaded area is the excess entropy E.

Comparing Equation (37) with Equations (35) and (36), we see that $h_{\mu }\left(L\right)$ may be viewed as the finite-*L* approximation to the entropy density $h_{\mu }$. Graphically, $h_{\mu }\left(L\right)$ is the two-point slope of the H(L) versus *L* curve; in other words, $h_{\mu }\left(L\right)$ is the discrete derivative of H(L). The convergence of $h_{\mu }\left(L\right)$ to $h_{\mu }$ is illustrated in Figure 2. The entropy density $h_{\mu }$ is indicated by a horizontal dashed line.

### 3.4. Density, Rate, and Algorithmic Complexity

Coming back to the issue of describing the observations of spin configurations we note that the entropy density $h_{\mu }$ is equivalent to the growth rate of the Kolmogorov–Chaitin (KC) complexity of spin configurations, averaged over a given ensemble [18,36]. The KC complexity of an individual configuration is defined as the length of the minimal program that, when run, will cause a universal Turing machine (UTM) to produce the configuration and then halt. The KC complexity is sometimes referred to as the algorithmic (or deterministic) complexity because it demands a deterministic accounting for every spin in the configuration. A random configuration by definition possesses no regularities so it cannot be compressed. As a result, a random configuration’s shortest description is the configuration itself. Hence, we see that the KC complexity is maximized by random configurations, as is the entropy density $h_{\mu }$.

### 3.5. Redundancy

If $h_{\mu }<\log_{2}\left|A\right|$, the full information carrying capacity of the alphabet is being underutilized. Said in a complementary fashion, in this case the information source produces sequences that have correlations. One measure of these correlations is the *redundancy* [17]:

(38) $$R\equiv \log_{2}\left|A\right|-h_{\mu }\;.$$

There is no redundancy in a completely random source, since by definition such a source has $\mathrm{Pr}\left(s^{L}\right)=U\left(s^{L}\right),\;L=1,2,\ldots$, and so $h_{\mu }=\log_{2}\left|A\right|$.

### 3.6. Shannon’s Coding Theorems

The sequence of yes-no questions leading to the identification of a particular outcome *x* of the random variable *X* defines a code for that outcome. One can show that the average (per symbol) length of the optimal, uniquely decodable binary encoding for the information source *X* lies between $h_{\mu }$ and $h_{\mu }+1$ [18]. If one tries to encode *N* copies of the variable *X*, the average length of the code approaches $Nh_{\mu }$ as N→∞. Thus, the entropy rate $h_{\mu }$ of the random variable *X* can also be interpreted as the average number of bits of memory needed to store information about the values *X* takes [17,18].

This view of entropy as average code length is in harmony with the notion of entropy as uncertainty. If we are very uncertain about the outcome of an observation, on average it will take a long code word to specify the outcome when it occurs. If we are fairly certain what the outcome will be, we can take advantage of this knowledge by using short code words for the frequently occurring outcomes. This strategy is employed in Morse code, where the most frequently occurring English letter “E” is encoded using the shortest symbol, one “dot”. Put somewhat colloquially, then, the entropy rate measures the average length (per symbol) required to describe observations of a random variable.

Now that the entropy density (or rate) has been defined we can quickly mention Shannon’s coding theorems again in order to show the utility of the various entropies just discussed and also to highlight one difference in motivation between information theory and statistical mechanics. The first coding theorem states that if $h_{\mu }>C$, the information source cannot be transmitted without errors. The second says that if $h_{\mu }<C$, then there exists an encoding of the source messages that produces a new source whose rate is less than, but arbitrarily close to C. So, by the first theorem, the source messages can be carried error free in a noisy channel of capacity C and correctly decoded. Exactly how one finds these encoding schemes is not specified by the theory, though many techniques have been developed since information theory’s introduction.

### 3.7. Two-Spin Mutual Information

We can use the mutual information to define an information-theoretic analogue of the two-spin correlation functions discussed above. The two-spin mutual information is defined by:

(39) $$I\left(r\right)\equiv I\left[S_{0};S_{r}\right]\;,$$

and measures the information shared between two spins separated by *r* sites. Using the translation invariance of spin configurations it follows that:

(40) $$I\left(r\right)=2H\left[S_{0}\right]-H\left[S_{0},S_{r}\right]\;.$$

Note that I(0)=H(1) and that for a typical source I(r) is monotone decreasing, I(r)≥I(r+1). For the special case of binary sequences in which Γ(r) vanishes as r→∞, $I\left(r\right)∼\Gamma^{2}\left(r\right),\;r\gg 1$ [75].

### 3.8. Excess Entropy

The entropy density $h_{\mu }$ measures the per-spin unpredictability of infinite configurations. However, $h_{\mu }$ says little about how difficult it is to perform this prediction. For example, consider two periodic configurations: one of period 4 and one of period 1969. Both have zero entropy density, indicating that once the periodic pattern is gleaned there is no uncertainty about the subsequent spins. However, there are important (and obvious) differences between the two configurations. It seems clear that, in some sense, the period-1969 configuration is “harder” to predict than the period-4 configuration; a distinction that is missed by stating $h_{\mu }=0$. For example, one would imagine that the configuration with the longer period requires much more memory to predict than that with the short period. How can we formalize the notions of “memory” and “difficulty” of prediction? For the remainder of this section and the following one, shall state this question more clearly and then answer it.

We begin our consideration of memory by observing that the length-*L* approximation to the entropy density $h_{\mu }\left(L\right)$ overestimates the entropy density $h_{\mu }$. Specifically, $h_{\mu }\left(L\right)$ overestimates $h_{\mu }$ by an amount $h_{\mu }\left(L\right)-h_{\mu }$ that measures how much more random single spins appear knowing the finite *L*-block statistics than knowing the statistics of the infinite configurations $\overset{↔}{S}$. In other words, this excess randomness tells us how much additional information must be gained about the configurations in order to reveal the actual per-spin uncertainty $h_{\mu }$. More precisely, the difference $h_{\mu }\left(L\right)-h_{\mu }$ is a form of redundancy, as discussed in Section 3.5 above. Though the source appears more random at length *L* by this amount, this amount is the information-carrying capacity in the *L*-blocks that is not actually random, but is due instead to correlations. We conclude that entropy convergence is related to a type of memory.

There are many ways in which the finite-*L* approximations $h_{\mu }\left(L\right)$ converge to their asymptotic value $h_{\mu }$. Recall Figure 2. Fixing the values of H(1) and $h_{\mu }$, for example, does not determine the form of the $h_{\mu }\left(L\right)$ curve. At each *L* we obtain additional information about how $h_{\mu }\left(L\right)$ converges, information not contained in the values of H(L) and $h_{\mu }\left(L\right)$ at smaller *L*. Thus, roughly speaking, each $h_{\mu }\left(L\right)$ is an independent indicator of the manner in which $h_{\mu }\left(L\right)$ converges to $h_{\mu }$.

Given that each increment $h_{\mu }\left(L\right)-h_{\mu }$ is an independent contribution in the sense just described, we sum up the individual *L*-redundancies to obtain our candidate measure of memory. The resulting quantity is the *total excess entropy* [24,26,27,29,31,32,76,77]:

(41) $$E\equiv \sum_{L=1}^{\infty }\left[h_{\mu }\left(L\right)-h_{\mu }\right]\;.$$

Shaw refers to E as the *stored information* [29], whereas Grassberger calls it the *effective measure complexity* [24]. Graphically, E is the shaded area in Figure 1. If one inserts Equation (37) into Equation (41), the sum telescopes and one arrives at an alternate expression for the excess entropy:

(42) $$E=\lim_{L\to \infty }\left[H\left(L\right)-h_{\mu }L\right]\;.$$

Hence, E is the *y*-intercept of the straight line to which H(L) asymptotes, as indicated in Figure 1. Note that the excess entropy is nonnegative, E≥0.

Looking at Equation (41), we see that, informally, E is the amount in bits, above and beyond $h_{\mu }$, of *apparent* randomness that is eventually “explained” by considering increasingly longer spin blocks. Conversely, to see the actual (asymptotic) randomness at rate $h_{\mu }$, we must extract E bits of information from observations of spin blocks. We expect a large E to indicate a large amount of structure: E is large if there are long-range correlations that account for the apparent randomness observed in distributions over small spin blocks. (Later on it will become clear that these interpretations must be substantially refined.)

These interpretations are strengthened by noting that E may be expressed as the mutual information *I* between the two semi-infinite halves of a configuration;

(43) $$E=I[\overset{\leftarrow }{S};\vec{S}]\;.$$

Note that this form makes it clear that E is spatially symmetric. Recalling that the mutual information can also be written as the difference between a joint and a conditional entropy:

(44) $$I\left[\overset{\leftarrow }{S};\vec{S}\right]=H\left[\vec{S}\right]-H\left[\vec{S}|\overset{\leftarrow }{S}\right]\;,$$

we see that E measures the average reduction in uncertainty $H[\vec{S}]$ of the right-half configuration $\vec{S}$ (the “future”), given knowledge of $\overset{\leftarrow }{S}$ (the “past”). One must interpret Equations (43) and (44) with care since they contain entropy contributions, like $H[\overset{↔}{S}]$, that individually may be infinite—even for a fair coin process.

Equations (43) and (44) allow us to interpret E as a measure of how much information one half of the spin chain carries about the other. In this restricted sense E measures the spin system’s *apparent* spatial memory. (We say “apparent” here in order to leave room for an important distinction made later.) If the configurations are perfectly random or periodic with period 1, then E vanishes. Excess entropy is positive between the two extremes of ideal randomness and trivial predictability. This property ultimately derives from its expression as a mutual information, since the mutual information between two variables vanishes either (i) when the variables are statistically independent or (ii) when they have no entropy or information to share. These extremes correspond to E vanishing in the cases of ideal randomness and trivial predictability, respectively. Finally, E measures the average degree of statistical independence of the two halves of a spin chain—how “indecomposable” the chain is.

Note that all three expressions for the excess entropy, Equations (41)–(43), indicate that E carries units of *bits*. This is clear in Equation (43), since the mutual information has units of bits. The entropy density, $h_{\mu }$ has units of bits per site, and *L*, the length of a spin block, has units of lattice sites. Hence, both terms on the right hand side of Equation (42) have units of bits, so it follows that the left hand side, E, must have units of bits as well. Lastly, Equation (41) tells us that E is the shaded area in Figure 1. The vertical axis of Figure 1 has units of bits per site while the horizontal axis has units of lattice sites. Since E is an area on Figure 1, it has units of bits.

It follows immediately that any periodic sequence of period P has $E=\log_{2}P$. Returning to the example at the beginning of this section, then, we see that a period-1969 sequence has an excess entropy $\log_{2}1969\approx 10.94$ bits, while the period-4 sequence has an excess entropy of $\log_{2}4=2$ bits. Thus, as anticipated, the period-1969 sequence does indeed possess more memory than the period-4 sequence.

### 3.9. Correlation Information

In the previous section we interpreted the excess entropy as the total amount of information that must be extracted from measuring *L*-blocks in order to recover the asymptotic entropy density—that is, to see just how random each spin is. The entropy convergence plot, Figure 2, is the discrete derivative, with respect to block length *L*, of the entropy growth curve H(L) of Figure 1. What if we take more discrete derivatives of H(L)? It turns out that the second derivative of H(L) recovers the correlation informations k(L) of Refs. [31,78] and allows for another interpretation of excess entropy.

We follow Refs. [31,78] and define the correlation information k(L) of order *L* as:

(45) $$k\left(L\right)=\sum_{\left\{s^{L}\right\}}\mathrm{Pr}\left(s^{L}\right)\log_{2}\frac{\mathrm{Pr}(s_{L-1}|s^{L-1})}{\mathrm{Pr}(s_{L-2}|s^{L-2})}\;.$$

It is easy to see that $k\left(L\right)=h_{\mu }\left(L-1\right)-h_{\mu }\left(L\right)$. Thus, the correlation informations are indeed (the negative of) a discrete derivative of the entropy convergence function $h_{\mu }\left(L\right)$. These quantities have a useful interpretation as the information gain between distant spins [78]. They can also be rewritten as the mutual information $I[s_{L-1};s^{L-1}]$ between the next spin $s_{L-1}$ and the preceding spin block $s^{L-1}$. Thus, k(L) is somewhat similar to the two-point mutual information just introduced.

From the boundary conditions on H(L), we see that $k\left(1\right)=h_{\mu }\left(1\right)=H\left(1\right)$. Note that $\lim_{L\to \infty }k\left(L\right)=0$. Under suitable assumptions about the source’s structure, it follows from the definition that the excess entropy is directly related to the correlation informations according to

(46) $$E=\sum_{L=1}^{\infty }Lk\left(L\right)\;.$$

In this form, E appears as type of correlation length: E is an average length in which the average is weighted by the correlation informations [31,78].

### 3.10. Information Theoretic Approaches to Structure in Dynamics, Statistical Physics, and Elsewhere

The total excess entropy was used by Crutchfield and Packard in Refs. [26,79,80,81] to examine the entropy convergence for noisy discrete-time nonlinear mappings. They developed a scaling theory for the entropy rate convergence: $h_{\mu }\left(L\right)-h_{\mu }\propto 2^{-\gamma L}$, where, for Markovian finite-memory chains, the excess entropy and entropy convergence exponent γ are simply related: $E=\left(H\left(1\right)-h_{\mu }\right)/\left(1-2^{-\gamma }\right)$. Analytical calculations of entropy convergence for several discrete-time nonlinear maps were carried out by Szépfalusy and Györgyi [27], by Csordás and Szépfalusy [82], and by Kaufmann [83].

Excess entropy was recoined “stored information” by Shaw [29] and subsequently “effective measure complexity” by Grassberger [24]. These two authors emphasize the entropy growth view shown in Figure 1. E has been discussed in the context of cellular automata by Grassberger [24] and by Lindgren and Nordahl [31]. Excess entropy was also mentioned briefly by Lindgren in Ref. [76]. The quantity is simply called “complexity” as applied to several stochastic automata by Li [32].

There have been other prior discussions of using information theory to measure a source’s structure. Most germane to the discussion above, Del Junco and Rahe used excess entropy to classify information sources in terms of their memory [77]. More generally, mutual information was proposed some time ago as a measure of self-organization [84,85,86]. Watanabe [87] and Kolmogorov [88] take approaches that are different, yet again. The latter is particularly notable, though brief, for how its discussion of source structure parallels the philosophies of model inference by minimum message length [89] and minimum description length [90] found in the theories of model order estimation and universal source coding. Both of these latter approaches address the discovery of source structure, though not as directly as concerns us here.

A number of the above notions have also recurred in more recent discussions of modeling information sources [34,91]. See also the references in Ref. [92] and the critical evaluation there of information-theoretic notions of complexity and structure.

It should be emphasized that there are subtle but significant differences in these works’ notions of effective complexity, memory, and information. One of the main subtleties is found in their differing approaches to minimality of the representations used to measure these quantities. Minimality is crucial for being able to conclude that a given quantity estimated from a model actually describes an intrinsic structural property of a process and is not an artifact of some unarticulated representational choice—a key issue to which we shall return repeatedly in the following. In contrast to the above, Refs. [89,90] are explicitly concerned with inferring minimal models and do so by balancing model size against model-induced error.

The preceding observations on the nature of entropy convergence stay within the framework of information theory—a largely statistical view of “structure”. These quantities and a number of the preceding observations have been known for at least a decade and a half, if not longer.

## 4. Computational Mechanics

In the previous section we saw that the excess entropy E provides a measure of the apparent spatial memory stored in configurations. However, excess entropy and the apparatus of information theory tell us nothing about *how* the system’s memory is organized and utilized. Computational mechanics [16] addresses this issue by paralleling and extending the architectural analyses found in discrete computation theory. (Basic textbooks for the latter are Refs. [93,94].) In explicitly considering a system’s apparent spatial memory E, we shall be led to put forth another measure of intrinsic memory, known as the statistical complexity and defined as the minimum amount of memory needed by any process to statistically reproduce a given configuration (and the ensemble from which it comes). This is a different interpretation of memory than given to the excess entropy. Section 4.5.6 and Section 5.1.6, however, will show that these two notions of memory are related. This additional set of theoretical tools allows us to describe structure and information processing at a more detailed and complete level than possible via information theory alone.

Like statistical mechanics, computational mechanics is concerned with a large system consisting of many individual components. However, computational mechanics addresses very different issues. The motivating questions of computational mechanics center around *how* a system processes information: How is information stored, transmitted, and transformed? How much memory is needed to statistically reproduce an ensemble of configurations and how is this memory organized? In general, we are interested in inferring the intrinsic computation being performed by the system itself.

By *intrinsic* computation [42] we mean something very different than “computation” as the word is typically applied either in reference to the use of modern digital computers as tools for simulation or for symbolic manipulation (e.g., as found in the Journal of Computational Physics) or in reference to the use of a device to perform useful information processing for some person or machine (as in updating a spreadsheet or determining the five billionth digit of π). Useful computation usually entails fixing the initial conditions and control parameters of a dynamical system so that the outcome contains some information of interest to us, as outside interpreters of some final state [95]. For example, we might employ the mapping

(47) $$x_{n+1}=\frac{1}{2}\left(x_{n}+\frac{a}{x_{n}}\right)\;,\;x_{0}=1\;,$$

which has the useful property that $\lim_{n\to \infty }x_{n}=\sqrt{a}$ [96]. This iterative procedure for increasingly accurate estimates of roots was reported by Hero of Alexandria [97] in the first century B.C.

In contrast, when we ask about intrinsic computation, we are interested not in manipulating a system to produce an output that is useful to us—which is akin to an engineering stance towards nature. Instead, we are interested in examining the information processing that the system itself performs and the underlying mechanisms that support it—which is more of a scientific stance: exploring how nature works on its own terms.

As a concrete example, consider the two-dimensional nearest-neighbor Ising model at the critical temperature. Here, the correlations between spins decay with a power law as a function of distance, yet the total magnetization of the system remains zero. Computational mechanics is concerned with what sorts of effective information processing the system must perform to reach and maintain the critical state. How much historical and spatial memory is required? How is the memory organized internally? What spatial patterns result? Are the critical configurations in any way “harder” to reach than those found at low or high temperatures? More informally, how does the system balance up and down spins so that the correlations decay as a power law, while keeping zero magnetization?

Whereas statistical mechanics starts with a system’s Hamiltonian or a description of its constituents’ local space-time behavior and interactions, computational mechanics begins with the joint probability distribution over the state space trajectories. With knowledge of this joint distribution, the intrinsic computation being performed by the system can be determined. By not requiring a Hamiltonian, computational mechanics can be applied in a wide range of contexts, including those where an energy function for the system may not be manifest.

In any case, as noted above, the two microscopic starting points in the many-body setting—a Hamiltonian or the joint probabilities of configurations over time—are related (at equilibrium) to each other by the usual canonical ensemble,

(48) $$\mathrm{Pr}\left(C\right)\;\propto \;e^{-\beta H(C)}\;,\;$$

where C is a configuration, β the inverse temperature, and H the system’s Hamiltonian.

### 4.1. Effective States: Preliminary Examples

Rather than launching into the mathematical development, we begin our review of computational mechanics with several very simple examples. These will lead quite naturally to the definitions put forth in the subsequent section.

The questions we shall be addressing for each example are: How can one statistically reproduce a given infinite configuration using the minimal amount of memory? In particular, how much information about the left half must be remembered to produce the right half? Here, *statistically reproduce* refers to the ability to generate infinite configurations whose finite-length spin blocks occur with the same probabilities as those in the original, infinite configuration.

Another, equivalent way of stating these questions is: How much memory is needed to optimally predict configurations? We define an *optimally predictive* model as one that correctly estimates all of a process’s statistics. Suppose we have a process described by the joint distribution $\mathrm{Pr}(\overset{↔}{s})$, which also determines other aspects of the process such as those described by various conditional and marginal distributions. Suppose in addition we have a model that uses a given amount of historical information to make its predictions. That is, we observe *L* spins $s^{L}$ in a configuration described by $\mathrm{Pr}(\overset{↔}{s})$. Using the information in $s^{L}$ the model produces an estimate $\hat{\mathrm{Pr}}\left(s^{K}|s^{L}\right)$ of the probability of the next *K*-spin block $s^{K}$. Then we say that the model optimally predicts the configuration (in the limit K,L→∞) if and only if $\hat{\mathrm{Pr}}\left(s^{K}|s^{L}\right)=\mathrm{Pr}\left(s^{K}|s^{L}\right)$ for all $s^{L}$ and all $s^{K}$, where $\mathrm{Pr}(s^{K}|s^{L})$ is obtained directly from $\mathrm{Pr}(\overset{↔}{s})$. In the examples to follow we will be implicitly arguing that the models presented are optimally predictive in this sense.

#### 4.1.1. Fair Coin Configuration

Consider a string of heads (H) or tails (T) generated by a fair coin toss:

(49) $${\overset{↔}{s}}^{\alpha }\equiv \cdots THTTTHHHHHTHTHTTHHT\cdots \;.$$

By definition all tosses are independently distributed; the probability that any particular toss is a heads is 1/2 and any particular length-*L* block has probability $2^{-L}$. We begin by asking: How much of the left half is needed to predict the values in the right half? Restated, imagine walking down the string from left to right, noting the state of the variables one observes. After a very long time—long enough for one to have observed as many tosses as desired—how many of the preceding variables must one keep track of in order to optimally predict those encountered later?

A moment’s reflection reveals that one does not need to keep track of any variables. Since the coin tosses are independent, knowledge of previous tosses does not reduce the uncertainty about the next toss. As a result, for this particularly simple example no memory is required to optimally predict subsequent variables. Here, the predictions are as good as they can be (i.e., optimal), which admittedly is not good at all. The uncertainty about the next coin toss is complete. The result could be either heads or tails with equal probability, as reflected by an entropy rate $h_{\mu }$ of 1 bit per toss for the fair coin.

**Figure 3:**
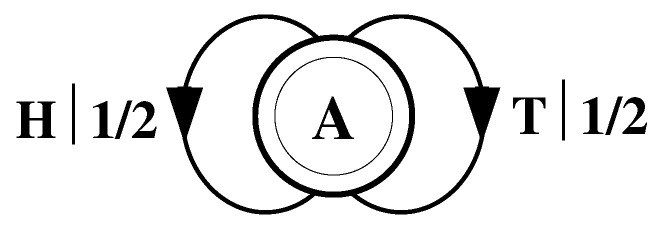
The probabilistic finite-state machine for fair coin tosses. This machine is a model of the original configuration, Equation (49), in the sense that a random walk through the machine—making state-to-state transitions following the edges, denoted s|p according the their labeled probability *p*—produces a sequential configuration of symbols $s_{i}\in A$ with the same statistical properties as the original ${\overset{↔}{s}}^{\alpha }$. For more discussion, see text.

What must one do in order to perform this optimal prediction? Equivalently, how can one statistically reproduce the configuration? The answer to these questions is illustrated by the probabilistic finite-state machine of Figure 3, which compactly tells us how to reproduce strings with the same statistics as the original configuration.

The machine operates as follows. Start in state A. With probability 1/2 generate an H and return to state A. Furthermore, with probability 1/2 generate a T and also return to state A. A random walk through the machine following these rules results in a string of H’s and T’s that is statistically identical to ${\overset{↔}{s}}^{\alpha }$. In this sense we say that the machine constitutes a model of the original fair coin process.

It is important to emphasize that no larger machine—i.e., with more states or edges—is required to reproduce all strings in the class of which ${\overset{↔}{s}}^{\alpha }$ is one realization. Nor is any smaller machine capable of doing so.

#### 4.1.2. Period-1 Configuration

Consider a string consisting of a sequence of all b’s:

(50) $${\overset{↔}{s}}^{\beta }\equiv \cdots bbbbbbbbbbbbbbbbbbbb\cdots \;.$$

As with the fair coin, it is clear that one does not need to remember previous symbols to perform optimal prediction. The value of the next variable will be a b regardless of the values of the previous variables.

**Figure 4:**
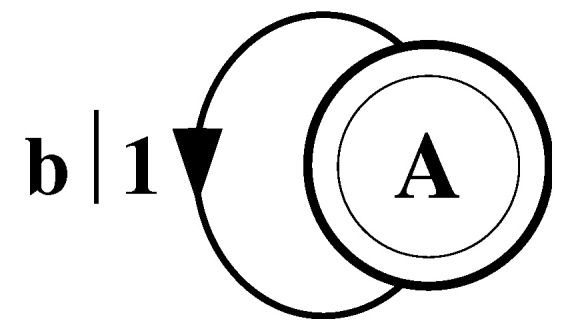
The finite-state machine for a string consisting of all b’s.

The finite-state machine for ${\overset{↔}{s}}^{\beta }$ is shown in Figure 4. From state A, the machine always outputs the symbol b and returns to state A. In this way the machine statistically reproduces ${\overset{↔}{s}}^{\beta }$. For this example the prediction is error free, as reflected in the fact that $h_{\mu }=0$.

#### 4.1.3. Period-2 Configuration

Now consider an infinite, alternating-spin configuration:

(51) $${\overset{↔}{s}}^{\gamma }\equiv \cdots ↑\;↓\;↑\;↓\;↑\;↓\;↑\;↓\;↑\;↓\;↑\;↓\;↑\;↓\;↑\;↓\;↑\;↓\cdots \;.$$

**Figure 5:**
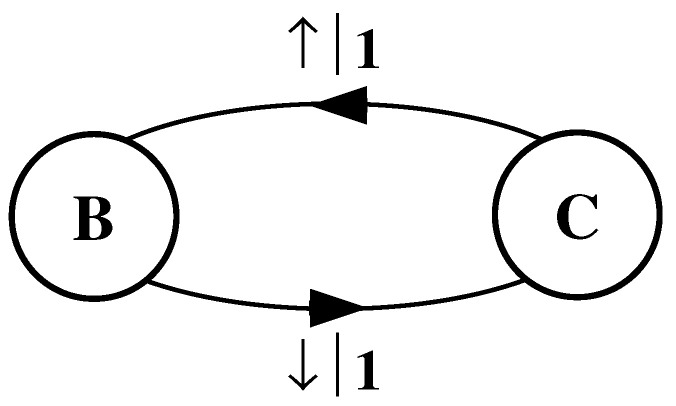
The recurrent portion of the finite-state machine for the period-2 configuration ${\overset{↔}{s}}^{\gamma }$ of Equation (51). Note that this machine has two states while the machines of Figure 3 and Figure 4 have only one state. This is an indication that the ⋯↑↓↑↓⋯ configuration requires more memory to reproduce.

Again, we begin by asking: How much of the left half is needed to predict spins in the right half? Here, some memory is needed to keep track of the phase of the alternating spin pattern. As long as this phase is remembered, one can optimally and exactly predict all the subsequent spins. As with the period-1 configuration, ${\overset{↔}{s}}^{\gamma }$ can be predicted with certainty since its entropy density is also $h_{\mu }=0$. However, to achieve this certainty, one must distinguish between the pattern’s two different phases. As a result, the state machine for ${\overset{↔}{s}}^{\gamma }$ has (at least) two states, as indicated in Figure 5.

How can we use the machine of Figure 5 to reproduce ${\overset{↔}{s}}^{\gamma }$? Unlike the previous examples, it is not clear where to begin: state B or state C? One response—consonant with assumptions implicit in equilibrium statistical mechanics—is that it does not matter. If we run the machine for infinitely long we will statistically reproduce the original configuration. The choice of starting state is just a “boundary condition” whose effects are negligible in the thermodynamic limit.

However, in another sense, the state in which we start most definitely *does* matter. Suppose we choose to start always in state B. We then examine all the length-3 spin blocks generated by this choice. We see that the string ↓↑↓ is generated with probability 1. Yet in the original configuration ${\overset{↔}{s}}^{\gamma }$ we observe Pr(↑↓↑)=1/2 and Pr(↓↑↓)=1/2. The machine of Figure 5 does not correctly predict the statistics of the configuration.

**Figure 6:**
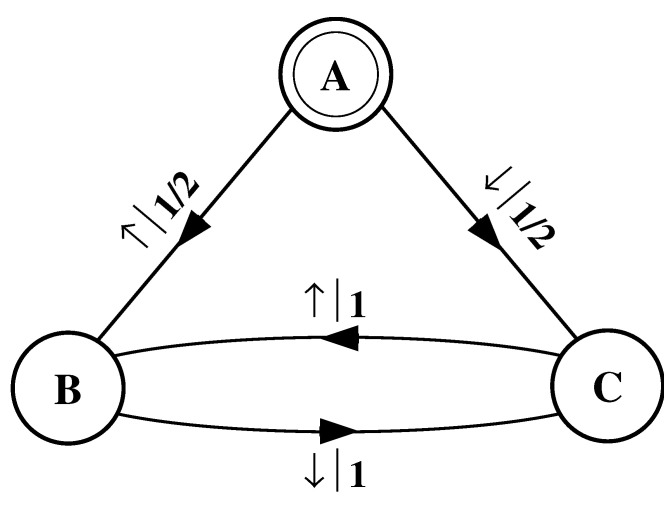
The full probabilistic finite-state machine for the period-2 configuration ${\overset{↔}{s}}^{\gamma }$. The start state A is indicated by the double circle. A is a transient state; it is never visited again after the machine outputs the first spin. States B and C are recurrent; they are visited infinitely often as the machine outputs an infinite spin configuration.

There is an easy remedy for this situation: start in state B half the time and state C half the time. We can achieve this by adding a *start state* to the model. This is shown in Figure 6. We now always begin operating our model in the unique start state A. In Figure 6 and all subsequent figures the start state is indicated by a double circle. The new, improved model generates spin blocks that exactly reproduce the distribution of finite-length spin blocks observed in the original configuration.

In this example, the start state is a transient state. It is never revisited after the machine outputs the first spin value and moves to state B or state C. The states B and C in Figure 6 are recurrent, being visited infinitely often as the machine is operated. When examining machines obtained from one-dimensional spin-1/2 Ising systems, we shall encounter examples where the start state is a recurrent state: as was the case, but not mentioned, in Figure 3 and Figure 4. We shall also see similar machines later on that have more than one transient state and that have more complex transient transition structures.

#### 4.1.4. Noisy Period-2 Configuration

Finally, consider an infinite binary string in which every other symbol sampled from the alphabet A={0,1} is a 0, but otherwise the symbols are unconstrained:

(52) $${\overset{↔}{s}}^{\delta }\equiv \cdots 01010001010100010000000100010\cdots \;.$$

**Figure 7:**
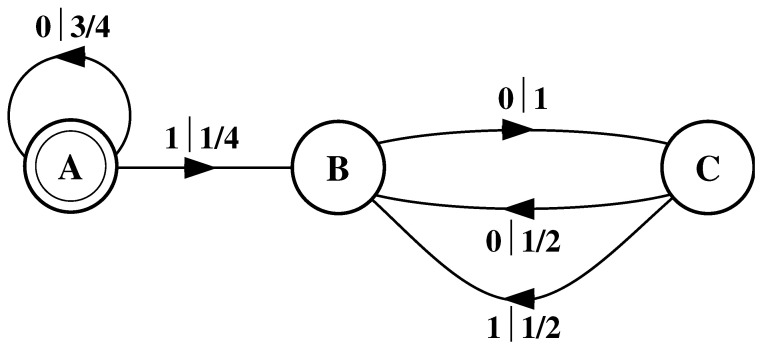
The probabilistic finite-state machine for the noisy period-2 configuration ${\overset{↔}{s}}^{\delta }$. Again, the start state A is a transient state and states B and C are recurrent.

Figuring out how to build a model capable of reproducing this configuration is perhaps not as straightforward as in the previous examples. The key realization is that once we observe a single 1 we are “synchronized” to the pattern. That is, after seeing a 1, a 0 *must* follow, since the configuration never exhibits two adjacent 1’s. After seeing the 0 that follows the first 1, a 0 or a 1 can occur with equal probability—the only rule is that every other symbol is 0. The probabilistic finite-state machine for this configuration is shown in Figure 7.

Note that the uncertainty associated with predicting the next symbol changes as one moves back and forth between state B and state C. From state B there is no uncertainty—a 0 is always the next symbol. From C there is an associated uncertainty of 1 bit, since the next symbol is equally likely to be a 0 or a 1. Thus, the entropy density is $h_{\mu }=1/2$ bit per symbol. It should not be immediately obvious how we determined the probabilities for transitions leaving state A. For this, we will need to review the general procedure for building such machines, as will be done below.

#### 4.1.5. Summary of Examples

Despite the examples’ simplicity, a few summarizing remarks are in order before moving on to formalize the notion of “effective” state that we just used implicitly.

First, note that the coin-toss configuration ${\overset{↔}{s}}^{\alpha }$ and the period-1 configuration ${\overset{↔}{s}}^{\beta }$ both result in a machine with only one state, an indication that we do not need to remember any information about the previous symbols to predict the values of the next. Thus, predicting a perfectly random process and a process with a very simple configuration are both “easy” tasks in the sense that they require small machines.

Second, note that entropy rate $h_{\mu }$ manifests itself (roughly) as the degree of branching in the machines, measured as the logarithm of the ratio of the number of edges to the number of states, in the recurrent portion of the machines. For example, in Figure 3 there are two edges leaving one state. The entropy rate is 1 bit per symbol.

Third, transient states tell one how the machines *synchronize*. For the period-2 example, we argued that the transient state A of the machine in Figure 6 was necessary so that the machine would faithfully reproduce the distribution over finite-size blocks. In a complementary way, the transient states are necessary for synchronizing the machine if one is reading in data from the configuration. Before any symbols are parsed, one does not know in which internal state the process was as it produced symbols in the configuration. This state of ignorance corresponds to the start state. Transitions are then taken from the start state corresponding to the symbols observed as the configuration is parsed. The number and structure of the transient states determine how difficult it is to synchronize—i.e., to determine in which recurrent state the system is as it produces each symbol.

Last, note that we have taken care to construct minimal machines. That is, the machines put forth are such that if one removes any state or transition then one can no longer exactly statistically reproduce the configuration. This notion of minimality will be made more precise below. In complementary fashion, in each example one gains no predictability by elaborating any of the machines by adding states or transitions.

### 4.2. Causal States and ϵ-Machines

The preceding section considered models capable of reproducing the distribution of all finite length blocks observed in several translationally invariant configurations. This section presents a general procedure for constructing such models—minimal, optimally predictive, probabilistic state machines.

First, we need to formalize the intuitive arguments through which the “effective” states of the four example systems were discovered. The key step is identifying the notion of effective state with the conditional probability distribution over right-half configurations. Furthermore, a central property is that the resulting representation be minimal. When constructing an optimally-predictive, minimal state-machine description, there is no need to distinguish between different left-half configurations that give rise to an identical state of knowledge about the right-half configurations that follow it. Maintaining a distinction between two such states adds to the model’s size without increasing its predictive ability. Therefore, we will be looking for the smallest set of predictive states.

To make these ideas precise, consider the probability distribution of all possible right halves $\vec{s}$ conditioned on a particular left half ${\overset{\leftarrow }{s_{i}}}^{L}$ of length *L* at site *i*: $\mathrm{Pr}(\vec{s}|{\overset{\leftarrow }{s_{i}}}^{L})$, 0≤L≤∞. For L=0, ${\overset{\leftarrow }{s_{i}}}^{L}$ is the empty string, denoted by λ. That is, $\mathrm{Pr}\left(\vec{s}|{\overset{\leftarrow }{s_{i}}}^{0}\right)\equiv \mathrm{Pr}\left(\vec{s}|\lambda \right)=\mathrm{Pr}\left(\vec{s}\right)$ denotes the probability of observing $\vec{s}$ unconditioned on any spins in the left half of the configuration.

We now use this form of a conditional probability to define an equivalence relation ∼ on the space of all left halves. We say that two left-half configurations at different lattice sites are equivalent (under ∼) if and only if they give rise to identical distributions over right-half configurations, conditioned on those left-halves. Formally, we define the relation ∼ by:

(53) $${\overset{\leftarrow }{s_{i}}}^{K}∼{\overset{\leftarrow }{s_{j}}}^{L}\;\mathrm{iff}\;\mathrm{Pr}\left(\vec{s}|{\overset{\leftarrow }{s_{i}}}^{K}\right)=\mathrm{Pr}\left(\vec{s}|{\overset{\leftarrow }{s_{j}}}^{L}\right)\;,$$

for all $\vec{s}$, where K,L=0,1,2,…. The induced equivalence classes are subsets of the set of all allowed ${\overset{\leftarrow }{s_{i}}}^{L}$. Appendix A reviews various properties of equivalence relations.

In a setting in which the conditional probabilities $\mathrm{Pr}(\vec{s}|{\overset{\leftarrow }{s_{i}}}^{L})$ are not known exactly, it becomes necessary as a practical matter to introduce some tolerance into the equivalence relation defined by Equation (53). Implementing this is not a straightforward task, since if one adds a tolerance δ and writes ${\overset{\leftarrow }{s_{i}}}^{K}∼{\overset{\leftarrow }{s_{j}}}^{L}\;\mathrm{iff}\;\mathrm{Pr}\left(\vec{s}|{\overset{\leftarrow }{s_{i}}}^{K}\right)=\mathrm{Pr}\left(\vec{s}|{\overset{\leftarrow }{s_{j}}}^{L}\right)+\delta$, the equivalence relation is destroyed because ∼ is no longer transitive; see Appendix A. We will address the issues surrounding the implementation of a tolerance elsewhere. Suffice it to say that the basic difficulty this introduces is common to other inference problems that involve statistical clustering and classification [98,99]. Since we are focusing here on processes for which one can perform the necessary calculations analytically, statistical estimation will not be a concern.

The equivalence classes over:

(54) $$\left\{{\overset{\leftarrow }{s_{i}}}^{L},\;i=\ldots ,-2,-1,0,1,2,\ldots \;,L=0,1,2,\ldots \right\}$$

induced by this relation are called *causal states* and denoted $S_{\alpha },\;\alpha =0,1,2,\ldots$. The $S_{\alpha }$ are the “effective states” of the previous section. Two histories, ${\overset{\leftarrow }{s}}^{L}$ and ${\overset{\leftarrow }{s}}^{K}$, belong to same causal state if, as measured by the probability distribution of subsequent spins conditioned on having seen each particular left-half configuration, they give rise to the same expectations about the configurations that follow to the right.

We shall use the convention that causal states $S_{\alpha }$ are generically indexed using Greek letters. Spin variables shall continue to be indexed with Roman letters. The equivalence class associated with $\mathrm{Pr}(\vec{s}|\lambda )$ is always the start state, since this distribution corresponds to the knowledge about right-half configurations before any spins are observed. The start state is denoted $S_{0}$.

The causal states, as determined by the equivalence classes induced by Equation (53), give transient as well as recurrent states. Consider the *predecessor set* $\left\{s^{L}\in S\right\}$ of length-*L* spin blocks that lead to causal state S. Transient states are those causal states that in the infinite sequence limit have a vanishing fraction (appropriately measured) of sequences in the predecessor set. (For a measure-theoretic treatment, see Ref. [41]). Although the size of their predecessor sets vanishes asymptotically, transient states can be visited an infinite number of times. Compare Figure 6 and Figure 7. In the former, as soon as a single spin value is read, one moves out of the transient state A. Thus, $\{s^{L}\in S\}=Ø,L>1$. Whereas in the latter figure, one eventually leaves the transient state A, but there is a single configuration—all 0s—that keeps one in A; $\left\{s^{L}\in S\right\}=\left\{0^{L}\right\}$.

In contrast, recurrent states are those visited infinitely often and that have large (positive measure) predecessor sets in the same limit. By considering how the process synchronizes—i.e., how it reaches the recurrent states as successively longer blocks are generated—it is possible to construct the transient states and their transitions from knowledge of the recurrent states alone. The procedure by which this is done is given in Appendix B.

We denote the set of causal states by $S=\{S_{\alpha },\alpha =0,\ldots ,k-1\}$. For the processes considered here S is discrete and k=|S| is finite—neither of which necessarily holds in a general setting [16,41]. Let $S^{\left(T\right)}$ denote the set of transient states and $S^{\left(R\right)}$ denote the set of recurrent states. Note that $S=S^{\left(T\right)}\cup S^{\left(R\right)}$.

There is a mirror image definition of causal states obtained by scanning the lattice in the opposite direction (right to left); which thus uses distributions conditioned on right-half configurations. Since we will study a restricted class of systems that respect this symmetry, the causal states will be the same regardless of the scanning direction. In the general case, in which this reversal symmetry need not hold, it is possible to find different causal states if one scans $\overset{↔}{s}$ in different directions [42].

Assuming scan-direction invariance, the causal states factor the joint distribution over configurations into conditionally independent distributions. That is, at time *t*, we have:

(55) $$\mathrm{Pr}\left(\overset{↔}{s}|S\right)=\mathrm{Pr}\left({\overset{\leftarrow }{s}}_{t}|S\right)\mathrm{Pr}\left({\vec{s}}_{t}|S\right)\;,$$

where S is the causal state to which the left half ${\overset{\leftarrow }{s}}_{t}$ has led. That is, having read ${\overset{\leftarrow }{s}}_{t}=\ldots s_{t-2}s_{t-1}$, S is the causal state arrived at after having observed the value $s_{t-1}$. By assuming scan-direction invariance it is the same causal state arrived at after having observed, from right to left, the last value $s_{t}$ in right-half configuration ${\vec{s}}_{t}=s_{t}s_{t+1}\ldots$. In this way, knowledge of the causal state determines the left- and right-half semi-infinite distributions. Put another way, the causal states are the (minimal) set of objects required to specify the entire joint distribution. Note that searching for and utilizing conditional factorings of joint distributions are central concerns in building statistical models for artificial intelligence and machine learning [100,101].

As we saw above, for the period-2 system there are 3 causal states, denoted in Figure 6 by A, B, and C. These causal states are subsets of the allowed ${\overset{\leftarrow }{s}}^{L}$:

(56) $$\begin{array}{ccc} A & = & \{\lambda \}\;, \end{array}$$

(57) $$\begin{array}{ccc} B & = & \left\{\;{\overset{\leftarrow }{s}}^{L}|s_{-1}=↑,s_{-2}=↓,s_{i}=s_{i+2},L\geq 1\right\}\\ & = & \{↑,↓↑,↑↓↑,↓↑↓↑,↑↓↑↓↑,\ldots \}\;,\; \end{array}$$

and

(58) $$\begin{array}{ccc} C & = & \left\{{\overset{\leftarrow }{s}}^{L}|s_{-1}=↓,s_{-2}=↑,s_{i}=s_{i+2},L\geq 1\right\}\\ & = & \{↓,↑↓,↓↑↓,↑↓↑↓,↓↑↓↑↓,\ldots \}\;. \end{array}$$

Here S={A,B,C}, $S^{\left(T\right)}=\left\{A\right\}$, and $S^{\left(R\right)}=\left\{B,C\right\}$.

Once the set S of causal states has been identified, we determine the transition probabilities $T_{\alpha \beta }^{\left(s\right)}$ between states upon seeing symbol s∈A. That is, we need to find:

(59) $$T_{\alpha \beta }^{\left(s\right)}\;\equiv \;\mathrm{Pr}\left(S_{\beta },s|S_{\alpha }\right)\;.$$

To understand the nature of the transition probabilities better, we rewrite Equation (59):

(60) $$\mathrm{Pr}\left(S_{\beta },s|S_{\alpha }\right)=\mathrm{Pr}\left(S_{\beta }|s,S_{\alpha }\right)\mathrm{Pr}\left(s|S_{\alpha }\right)\;.$$

Knowledge of the next spin’s value *s* uniquely determines the subsequent causal state $S_{\beta }$. To see this, note that moving one step to the right corresponds to moving from ${\overset{\leftarrow }{s}}_{i}$ to ${\overset{\leftarrow }{s}}_{i+1}={\overset{\leftarrow }{s}}_{i}s$. The new left-half configuration ${\overset{\leftarrow }{s}}_{i+1}$ is associated with one and only one causal state. This follows by definition, since ∼ partitions the set of left-halves; see Appendix A. Hence, observing the next spin value *s* determines the next causal state $S_{\beta }$, as the chain is parsed from left to right.

This is the sense in which the causal state representation is *deterministic*. A transition from state α to state β while outputting a symbol *s* is uniquely determined by α and *s*. That is, $\mathrm{Pr}(S_{\beta }|s,S_{\alpha })=1$ in Equation (60), assuming the transition is allowed. To illustrate this, consider the noisy period-2 machine of Figure 7. From state B, outputting a 0 leads one to state C. Furthermore, from state C, seeing a 1 determines that the next state will be B. Note, however, that knowledge of the initial and final causal states does *not* determine what symbol was produced as the transition was made. For example, either a 0 or a 1 can be produced upon a transition from state C to B.

Equation (60) indicates how to obtain the transition probabilities—now seen to be simply given by $T_{\alpha \beta }^{\left(s\right)}=\mathrm{Pr}\left(s|S_{\alpha }\right)$—from the joint probabilities over configurations. Let ${\vec{s}}^{L}=s_{0}s_{1}\cdots s_{L-1}$ be a spin block that leads to, and belongs to, the causal state $S_{\alpha }$. Then:

(61) $$\begin{array}{cc} T_{\alpha \beta }^{\left(s\right)} & =\mathrm{Pr}\left(s|S_{\alpha }\right)=\frac{\mathrm{Pr}(s_{0}s_{1}\ldots s_{L-1}s)}{\mathrm{Pr}(s_{0}s_{1}\cdots s_{L-1})}\;. \end{array}$$

where β indexes the causal state $S_{\beta }$ to which one is taken on *s*. In other words, $s_{0}s_{1}\ldots s_{L-1}s\in S_{\beta }$.

Summing over the spin values *s*, we obtain the stochastic connection matrix $T=\sum_{s\in A}T^{\left(s\right)}$, a matrix whose components $T_{\alpha \beta }$ give the probability of a transition from the $\alpha^{\mathrm{th}}$ to the $\beta^{\mathrm{th}}$ causal state;

(62) $$T_{\alpha \beta }\equiv \mathrm{Pr}\left(S_{\beta }|S_{\alpha }\right)\;.$$

Since the probabilities are normalized, $\sum_{\beta }T_{\alpha \beta }=1$, and so T is a stochastic matrix. That is, the probability of making a transition is unity. The probability $\mathrm{Pr}(S_{\alpha })$ of finding this “internal” Markov chain in the $\alpha^{\mathrm{th}}$ causal state after the machine has been scanning infinitely long is the left eigenvector of T associated with eigenvalue 1, normalized in probability. That is, $\mathrm{Pr}(S_{\alpha })$ is given by:

(63) $$\sum_{\alpha =0}^{k-1}\mathrm{Pr}\left(S_{\alpha }\right)T_{\alpha \beta }=\mathrm{Pr}\left(S_{\beta }\right)\;.$$

(In this, we are assuming that the internal Markov chain is irreducible; its recurrent states are strongly connected). The asymptotic probability of all transient states is zero;

(64) $$\mathrm{Pr}\left(S_{\alpha }\right)=0,\;S_{\alpha }\in S^{\left(T\right)}\;.$$

For the period-2 machine of Figure 6 we have:

(65) $$T^{\left(s=↑\right)}=\left(\begin{array}{ccc} 0 & 1/2 & 0\\ 0 & 0 & 0\\ 0 & 1 & 0 \end{array}\right)$$

and

(66) $$T^{\left(s=↓\right)}=\left(\begin{array}{ccc} 0 & 0 & 1/2\\ 0 & 0 & 1\\ 0 & 0 & 0 \end{array}\right)\;,$$

where we take column and row labels to correspond to causal states in the natural way: 1↦A,2↦B, and so on. We add Equations (65) and (66) to obtain the machine’s stochastic connection matrix:

(67) $$T=\left(\begin{array}{ccc} 0 & 1/2 & 1/2\\ 0 & 0 & 1\\ 0 & 1 & 0 \end{array}\right)\;.$$

Note that T is stochastic and its dominant left eigenvector, normalized in probability, is (0,1/2,1/2). Hence, the asymptotic probability of the transient state A is zero, Pr(S=A)=0, and Pr(S=B)=Pr(S=C)=1/2.

The set S together with the dynamic $\left\{T^{\left(s\right)},s\in A\right\}$ constitute a model—referred to as an *ϵ-machine* [25]—of the original process. The four example machines of the previous section, Figure 3, Figure 4 and Figure 5 and Figure 7, are all ϵ-machines. An ϵ-machine is the minimal representation that captures the intrinsic computation being performed by the system under study in the sense that it explicitly lays out how information in the left-half configuration is stored in the causal states and determines the range of right-halves that can be seen along with their probabilities. In other words, an ϵ-machine shows how much memory a process has, how it is organized, and how it is used to generate the pattern exhibited by the process.

An ϵ-machine is minimal in the sense that one cannot remove a state or a transition and still have it be optimally predictive. Minimality follows immediately from the definition of the equivalence relation, Equation (53), [25,102]. The equivalence classes induced by the relation are associated with the causal states. The procedure of forming equivalence classes ensures that we distinguish between only those states that give rise to different predictive information. As a result,

(68) $$\mathrm{Pr}\left(s|S_{\alpha }\right)\neq \mathrm{Pr}\left(s|S_{\beta }\right)\;,$$

for α≠β and for at least one value of *s*. Recall that we demand that the ϵ-machine be capable of statistically reproducing the original configuration. If we make our machine smaller by merging two states, say α and β, then it follows immediately from Equation (68) that the machine will no longer be able to exactly statistically reproduce the original configuration since it fails to distinguish between the different conditional probabilities of Equation (68). Thus, we conclude that an ϵ-machine is minimal—a property which is *derived* and not added on as an axiom to force a desired feature.

The “ϵ” in the ϵ-machine signifies that, in general, the measurement values s∈A are not direct indicators of the observed process’s internal states [42]. For example, the symbols may be discretizations of variables that are continuous in state, space, or time. For spin systems these concerns are not at issue, since we know by definition the full set of elementary measurement values, i.e., the range of spin values at each site.

In the following, we determine ϵ-machines beginning with the Hamiltonian assumed for our model spin systems. However, as mentioned above, a Hamiltonian is not necessary. The determination of an ϵ-machine does not depend on knowledge of the dynamic or rule through which the configurations were generated. Moreover, the causal states and their transition probabilities may be calculated within two different paradigms; one mathematical, the other empirical. In the first, one begins with the joint distribution over all the system variables. In the second, one is given configurations from which the joint and various conditional distributions are estimated. The overall procedure in the second setting is referred to as ϵ-machine *reconstruction*. In either case, the goal is to factor the joint distribution over spin configurations into the causal state conditional distributions. The result is an ϵ-machine that consists of the components $\left\{S,\left\{T^{\left(s\right)}\right\},A,S_{0}\right\}$, where $S_{0}\in S$ is the ϵ-machine’s unique start state.

### 4.3. Related Computational and Statistical Model Classes

It is instructive to compare the representation class of ϵ-machines to others found in computation theory and statistics. First, let’s restrict attention to ϵ-machines for 1D finite-range spin systems. If we ignore the transition probabilities, and so distinguish only between allowed and disallowed transitions, we change the ϵ-machine representation into a special class of deterministic discrete-state automata [93,94]. Unlike this general class of automata, these nonprobabilistic ϵ-machines have the following properties: (i) a unique start state, (ii) all states are accepting, (iii) all recurrent states form a single strongly connected component in the machine’s state transition graph, and (iv) the set of states is minimal.

A further restriction on these nonprobabilistic ϵ-machines is that there is a specific relationship between the structure of the transient states and the recurrent states. (This relationship does not hold in general for discrete-state automata.) That is, the nonprobabilistic ϵ-machine’s transient states can be constructed from knowledge of the recurrent causal states alone. Appendix B gives a procedure that determines this relationship for the unrestricted, probabilistic case.

Unlike discrete-state automata, however, ϵ-machine transitions are labeled with conditional probabilities $T_{\alpha \beta }^{\left(s\right)}$. Said differently, an ϵ-machine represents a configuration distribution, not just a set of allowed configurations, as the automata representations would. Therefore, in important ways ϵ-machines are a richer class of representations.

The ϵ-machines considered here can also be viewed as a type of Markov chain. First, the stochastic connection matrix T, which describes only the state-to-state transitions unconditioned by spin values, is a Markov chain over the causal states. Second, and more directly, the full ϵ-machine, including spin labelings, is a subset of models called variously *functions of Markov chains* [103], *probabilistic discrete-state automata* [104], or *stochastic deterministic finite automata* [16,41], since the output (spin) alphabet A differs from the internal (causal) state “alphabet” S. To be more specific, an ϵ-machine is a function of a Markov chain that has a unique start state and one recurrent component. These, in turn, are a subclass of *hidden Markov models* [105].

For more general processes than 1D finite-range spin systems ϵ-machines typically do not reduce to functions of a Markov chain or to probabilistic analogs of discrete-state automata. For example, ϵ-machines can have a countable infinity or even a continuum of causal states [16,41]. Thus, ϵ-machines are best first considered on their own terms—a different model class that captures different types of structure. When connections to existing representations can be made, though, one often finds interesting structural features and can use existing theory to describe them.

### 4.4. What Do ϵ-Machines Represent?

Given that ϵ-machines can be related to this range of statistical and computational model classes, it is important to note that an essential distinguishing feature of computational mechanics is its hierarchical inductive framework. It begins by trying to model the original process using the *least* powerful model class. Probabilistic finite-memory machines are employed first; that is, we simply assume k=|S| is finite. However, using a finite-memory representation may not yield a finite-size model: the number of causal states could turn out to be countably infinite, as noted above, or to lie in a fractal set or in a continuum [16,41]. If this is the case, a more powerful model than a finite-state machine must be used. One proceeds by trying to use the next most powerful model classes in a hierarchy of machines known as the causal hierarchy [16]. The latter is an analog of the Chomsky discrete-computation hierarchy of formal language theory [93,94].

It was suggested in the introduction that, in a statistical mechanics context, using the most compact mathematical entity that provides a complete description of a system is an important way to distinguish between systems that are structured in different ways. The determination of an ϵ-machine may be thought of as a formalization of this process of detection and classification of structure. An ϵ-machine, the set of causal states and their transitions, provides a direct description of the structure present in the joint probabilities over the system’s internal degrees of freedom. In particular, the ϵ-machine’s organization shows how this joint distribution factors into conditionally independent components. Thus, determining the class of ϵ-machine that provides a finite description of the original configuration allows one to distinguish between systems that are organized in fundamentally different ways.

Furthermore, an ϵ-machine gives a minimal description of the pattern or regularities in a system in the sense that the pattern *is* the algebraic structure determined by the causal states and their transitions. If, for example, the ϵ-machine has an algebraic structure that is a group, then it captures a symmetry: for example, translational or spin-flip. That is, it captures the “pattern” exhibited in the system’s configurations. Generally, though, the algebraic structure is a semigroup—and a stochastic one at that—and so not obviously interpreted in terms of symmetries. The appropriate mathematical descriptions are given in terms of measure semigroups; see, for example, Ref. [106]. Despite a lack of familiar interpretations in this more general setting, the algebraic structure still captures the intrinsic “pattern” [107]. Examples will be given below as concrete illustrations of this algebraic view of pattern.

In summary, an ϵ-machine is a model of a system’s allowed-configuration ensemble. From this model, we can proceed to define and calculate macroscopic, global properties that reflect the characteristic average information processing capabilities of the system. We now turn to discuss just what features are calculable from an ϵ-machine.

### 4.5. Global Spatial Properties from ϵ-Machines

#### 4.5.1. Statistical Complexity

An ϵ-machine is a model capable of statistically reproducing a process’s configurations. How much memory is needed on average to operate this machine? Similarly, how little internal memory could the generating process itself have used? Motivated by these questions we now define a new quantity.

To predict successive spins as one scans a configuration from left to right, one must track in which causal state the process is, since knowledge of the causal state gives the required conditional distribution for optimal prediction. Thus, by the same arguments given earlier that motivate and interpret the Shannon entropy H[X] of an information source *X* as the average minimal code size, the informational size of the distribution Pr(S) over causal states, as measured by the Shannon entropy, gives the minimum average amount of memory needed to optimally predict the right-half configurations. This quantity is the *statistical complexity* [25]:

(69) $$\begin{array}{cc} C_{\mu } & \equiv H\left[S\right]=-\sum_{\alpha =0}^{k-1}\mathrm{Pr}\left(S_{\alpha }\right)\log_{2}\mathrm{Pr}\left(S_{\alpha }\right)\;, \end{array}$$

where, again, $\mathrm{Pr}(S_{\alpha })$ is given by Equation (63). Like the excess entropy E, the statistical complexity $C_{\mu }$ is a measure of memory and has units of bits. Note, however, that the two measures of memory have *different* interpretations. The excess entropy measures the *apparent* memory stored in the configurations, since it is determined directly from the spin-block distribution; that is, from the spin observables. In contrast, $C_{\mu }$ measures the minimal amount of (hidden) memory needed to statistically reproduce the configuration ensemble. As we shall see below, these two measures of memory, though related, typically are *not* equal.

Another, coarser measure of the ϵ-machine’s size is simply the number of recurrent causal states. This motivates the definition of the *topological complexity* $C_{0}$ as the logarithm of the number of causal states [16];

(70) $$C_{0}=\log_{2}\left|S^{\left(R\right)}\right|\;.$$

The topological complexity gives a simple “counting” upper bound on the statistical complexity: $C_{\mu }\leq C_{0}$. This follows from a basic maximization property of Shannon entropy applied to a uniform distribution over the (recurrent) causal states. ($C_{0}$ should not be confused with the topological complexity of Ref. [108].)

#### 4.5.2. Block Distributions and Entropies

We claimed above that an ϵ-machine is a model of a configuration in the sense that it reproduces the spin-block distributions $\mathrm{Pr}(s^{L})$. We now show explicitly how these distributions follow from the recurrent portion of an ϵ-machine. In subsequent sections we will then be able to easily calculate the various information-theoretic and statistical-mechanical quantities defined earlier.

First, note that the sequence of causal states is Markovian. The probability of a transition from recurrent state $S_{\alpha }$ to recurrent state $S_{\beta }$ is given by $T_{\alpha \beta }$. Hence,

(71) $$\mathrm{Pr}\left(S_{\alpha },S_{\beta }\right)=\mathrm{Pr}\left(S_{\alpha }\right)T_{\alpha \beta }\;.$$

The probability that the particular sequence $S_{\alpha_{0}}\cdots S_{\alpha_{L-1}}$ occurs is given by:

(72) $$\mathrm{Pr}\left(S_{\alpha_{0}}\cdots S_{\alpha_{L-1}}\right)=\mathrm{Pr}\left(S_{\alpha_{0}}\right)\prod_{i=0}^{L-2}T_{\alpha_{i}\alpha_{i+1}}\;.$$

However, we are interested in the distribution of spin blocks, as well as sequences of causal states. Recall that $T_{\alpha \beta }^{\left(s\right)}\equiv \mathrm{Pr}\left(S_{\beta },s|S_{\alpha }\right)$ is the probability of making a transition from state α to state β while producing the spin *s*. Each (α,β)-entry in the *word matrix* $T^{s^{L}}=T^{\left(s_{0}\right)}T^{\left(s_{1}\right)}\cdots T^{\left(s_{L-1}\right)}$ gives the probability of seeing word $s^{L}=s_{0}s_{1}\ldots s_{L-1}$ starting in state α and ending in state β. Using this matrix we can easily write down an expression for the probabilities over spin blocks:

(73) $$\mathrm{Pr}\left(s^{L}\right)=\sum_{\alpha =0}^{k-1}\mathrm{Pr}\left(S_{\alpha }\right)T_{\alpha \beta }^{s^{L}}\;.$$

Here we sum over the probabilities of all sequences of L+1 causal states, selecting only those for which the particular spin sequence $s_{0}\cdots s_{L-1}$ occurs.

Given the joint distribution over spins blocks, Equation (73), the block entropies H(L) follow immediately from Equation (33).

#### 4.5.3. Two-Spin Mutual Information and Correlation Function

Recall that using the translation invariance of the spin configurations, the two-spin mutual information of Section 3.7 was given by:

(74) $$I\left(r\right)\;=\;2H\left[S_{0}\right]-H\left[S_{0},S_{r}\right]\;.$$

The second entropy term on the righthand side requires calculating the joint distribution $\mathrm{Pr}(s_{0},s_{r})$ and the first requires $\mathrm{Pr}(s_{0})$. In the previous section we derived an expression for $\mathrm{Pr}(s_{0})$, Equation (73). Thus, to calculate I(r) we need to develop an expression for $\mathrm{Pr}(s_{0},s_{r})$.

$\mathrm{Pr}(s_{0},s_{r})$ is easy to obtain by summing over all intervening spins in Equation (73):

(75) $$\begin{array}{ccc} \mathrm{Pr}(s_{0},s_{r}) & = & \sum_{s_{1},\cdots ,s_{r-1}}\mathrm{Pr}\left(s_{0}s_{1}\cdots s_{r-1}s_{r}\right) \end{array}$$

(76) $$=\sum_{\alpha =0}^{k-1}\mathrm{Pr}\left(S_{\alpha }\right)\sum_{s_{1},\cdots ,s_{r-1}}T_{\alpha \beta }^{s_{0}\ldots s_{r}}$$

(77) $$=\sum_{\alpha ,\gamma ,\delta =0}^{k-1}\mathrm{Pr}\left(S_{\alpha }\right)T_{\alpha \gamma }^{\left(s_{0}\right)}T_{\gamma \delta }^{r-1}T_{\delta \beta }^{\left(s_{r}\right)},$$

since $T_{\alpha \beta }^{r-1}=\sum_{s_{1},\cdots ,s_{r-1}}T_{\alpha \beta }^{s_{1}\ldots s_{r-1}}$ and where $T^{r}$ denotes the $r^{\mathrm{th}}$ power of the connection matrix T. The last equality follows since the summation over the α’s has the effect of multiplying together the *T* matrices.

The Shannon entropy of $\mathrm{Pr}(s_{0},s_{r})$ is $H[S_{0},S_{r}]$ and the entropy of $\mathrm{Pr}(s_{0})$ is $H[S_{0}]$ and so I(r), Equation (40), follows immediately.

Using these same distributions it is now possible to calculate Γ(r), the two-spin correlation function of Equation (13), since

(78) $$\Gamma \left(r\right)=\sum_{s_{0},s_{r}}s_{0}s_{r}\mathrm{Pr}\left(s_{0},s_{r}\right)-{\left(\sum_{s_{0}}s_{0}\mathrm{Pr}\left(s_{0}\right)\right)}^{2}\;.$$

The structure factors and susceptibility follow directly from Γ(r). Thus, all of these quantities can be readily calculated once an ϵ-machine is in hand.

#### 4.5.4. ϵ-Machine Entropy Rate

Recall from Equation (36) that the entropy density $h_{\mu }$ can be expressed as the entropy of one spin conditioned on all those spins preceding it. Using this, it is not hard to show that the entropy density can be expressed as the next-spin uncertainty averaged over the causal states:

(79) $$h_{\mu }=-\sum_{\alpha =0}^{k-1}\mathrm{Pr}\left(S_{\alpha }\right)\sum_{s\in A}\mathrm{Pr}\left(s|S_{\alpha }\right)\log_{2}\mathrm{Pr}\left(s|S_{\alpha }\right)\;,$$

where $\mathrm{Pr}(S_{\alpha })$ is given by Equation (63) and $\mathrm{Pr}(s|S_{\alpha })$ is given by Equation (61).

This result is similar to, but not the same as, that originally given in App. 4 of Ref. [17] for Markov chains. We derive it here in Appendix C. Our result is not surprising given the definition of causal states, which groups together left-half configurations that led to the same conditional distribution over possible right-half configurations. As a result, to calculate the entropy density $h_{\mu }$ one only need consider the entropy of a single spin conditioned on the current causal state.

The entropy rate is invariant under a change in the direction in which the configuration is scanned [42]. This fact is quite general and holds for any one-dimensional stationary process, a class of systems much broader than the spin systems considered here.

The minimality of ϵ-machines has an important consequence for estimating, or even analytically calculating, the entropy rate $h_{\mu }$ of a process. Merge any causal states in an ϵ-machine, in a manner leaving a well-defined machine that still generates the same set of configurations, and the entropy rate ${\hat{h}}_{\mu }$ of the modified machine is increased: ${\hat{h}}_{\mu }>h_{\mu }$ [102]. In other words, any representation smaller than the correct ϵ-machine will result in a machine that has an entropy rate higher than the original source. Thus, it follows that such a machine cannot possibly perform optimal prediction in the sense explained in Section 4.1.

#### 4.5.5. ϵ-Machine Excess Entropy

The excess entropy E can also be calculated from the probabilities of the causal states and their transitions. In the most general setting there is no compact formula for E in terms of Pr(S) and Pr(s|S), as there was for $h_{\mu }$. However, we shall see below in Section 5.1.5 that for the special case of finite-range spin systems considered here, it is possible to write down a relatively simple formula for E in terms of an ϵ-machine.

#### 4.5.6. Relationships between Measures of Memory

As remarked above, the excess entropy and the statistical complexity are different measures of a system’s memory. However, it turns out that the excess entropy sets a lower bound on the statistical complexity:

(80) $$E\leq C_{\mu }\;.$$

This result holds for any translationally invariant infinite configuration [43,109]. (Cf. Ref. [24] for a comparison of E and the *true measure complexity*.) Thus, the memory needed to perform optimal prediction of the right-half configurations can exceed the mutual information between the left and right halves themselves. This relationship reflects the fact that, in the general setting, a process’s internal state sequences are not in one-to-one correspondence with *L*-block or even *∞*-length configurations.

#### 4.5.7. The examples Analyzed Quantitatively

In Table 1 we show the results of calculating (largely by direct inspection) the entropy density $h_{\mu }$, the excess entropy E, and the statistical complexity $C_{\mu }$ for the example processes of Section 4.1.

#### 4.5.8. Scan-Direction Invariance

Interestingly, one can show that for some classes of systems (not including the finite-range spin systems examined here) $C_{\mu }$ and $C_{0}$ *are not* scan-direction invariant [42]. That is, the causal states, and as a result $C_{\mu }$ and $C_{0}$, may be different depending the direction in which the configuration is scanned: left to right or right to left. In contrast, the values of the entropy rate $h_{\mu }$, the excess entropy **E**, and the two-spin mutual information I(r) *are* independent of the direction in which the configuration is observed. This scan-direction invariance derives from these quantities’ definitions and is not a result which is particular to the spin systems considered here.

#### 4.5.9. Related, or Not, “Complexity” Measures

As noted above, an ϵ-machine is a model of the original process that uses the least powerful computational class admitting a finite model [16]. In contrast, Kolmogorov–Chaitin (KC) complexity characterizes symbol sequences by considering their representation in terms of the most powerful of the discrete computational model classes, the universal Turing machine (UTM). This is a deterministic representation and so must explicitly account for randomness and fluctuations in sequences.

Note that $C_{\mu }>0$ and E>0 do not imply that memory resources are expended to account for the randomness or thermal fluctuations present in a system. Thus, these measures of structural complexity depart markedly from the KC (deterministic UTM) complexity. As noted above, the ensemble-averaged per-site KC complexity is $h_{\mu }$ [18,36]. So, the KC complexity is dominated by a system’s random components. In turn, this masks algebraic symmetries and structural properties, unless the entropy rate is zero.

One unfortunate shortcoming of KC complexity, and its framework, is that it is in general uncomputable [18,36]. That is, unlike statistical complexity and excess entropy, there exists no general algorithm for its calculation. It should be noted, however, that in special cases such as finite-state Markov chains [18] or continuous-state dynamical systems with an absolutely continuous invariant measure [110], the *average value of the growth rate* of the Kolmogorov–Chaitin complexity can be calculated and is equal to the Shannon entropy rate $h_{\mu }$ of the process.

A quantity more closely related to statistical complexity and excess entropy is the *logical depth* of Bennett [30]. Whereas the Kolmogorov–Chaitin complexity of a symbol string is defined in terms of deterministic-UTM program length, the logical depth is defined as the time needed for the UTM, running the minimal program, to produce the string. On the one hand, if a configuration, like ${\overset{↔}{s}}^{\alpha }$ of Equation (49), is random, the shortest UTM program that reproduces it is the program “Print(${\overset{↔}{s}}^{\alpha }$).”. (The “.” delimits the program and indicates when the UTM can stop reading the input.) This is a relatively long program but takes very little time to run: a time proportional to the length of ${\overset{↔}{s}}^{\alpha }$. On the other hand, if a configuration has a simple pattern, like ${\overset{↔}{s}}^{\beta }$’s string of all b’s, then the program to reproduce it also takes a short time to run: a time proportional to the number of b’s to print. The minimal program is also short: all the UTM needs to do is loop over the command “Print b”, counting up to the desired string length. However, if a spin configuration has a great deal of intricacy—for example, if the spins code for the binary expansion of π—then the minimal program to reproduce it will involve many operations, many more than the number of desired spins.

As a result, like excess entropy and statistical complexity, the logical depth captures a property—being low for both simple and random configurations—that is distinct from randomness and from those properties captured by the entropy rate and Kolmogorov–Chaitin complexity. While there are superficial similarities, however, $C_{\mu }$ and E are measures of memory while logical depth is a measure of run time. A shortcoming of logical depth shared with KC complexity is that it is in general uncomputable [18,36]. That is, there exists no general algorithm for its calculation.

Another quantity, related to logical depth and even closer to the statistical complexity, is the *sophistication* of Koppel [111]. The sophistication measures the minimal “invariant” portion—i.e., the “program” part—of the UTM input as the size of the desired output string increases.

Probably the closest alternative to statistical complexity is found in the semigroup theoretic approach introduced by Rhodes [112]. The focus is similar in its development of measures of complexity that capture the structural decomposition of processes; see, for example, Ref. [113].

For other approaches to statistical complexity and correlational structure see Refs. [23,34,35,92,114,115] and citations therein.

#### 4.5.10. ϵ-Machine Thermodynamics

As a final note, we mention that ϵ-machines also provide a direct way to calculate the thermodynamic potentials for a process. These are also known as the fluctuation spectrum, the Renyi entropy, the spectrum of singularities, S(U) curves, and f(α) curves [22,82,116,117]. The fluctuation spectrum provides a measure of how likely a system is to deviate from its average behavior and is closely related to more modern methods, as found in the theory of large deviations [118,119], to describe a process’s behavior outside of the range of validity of the law of large numbers.

In Ref. [116] it was shown that calculating the fluctuation spectrum by first determining the ϵ-machine and then proceeding to calculate the spectrum from it yields significantly more accurate results than estimating the spectrum directly from configurations by using histograms to estimate spin-block probabilities. Finally, one can analyze the fluctuation spectra of causal state sequences themselves by replacing the Shannon entropy in the definition of statistical complexity, Equation (69), with the Renyi entropy [120]. It would be interesting to compare this to the Renyi entropy generalization of the excess entropy analyzed by Csordás and Szépfalusy [82].

### 4.6. Summary and a Look Ahead

In the previous sections we reviewed the tools used by statistical mechanics, information theory, and computational mechanics to measure correlation and structure. The main quantities from statistical mechanics are correlation functions Γ(r), the correlation length ξ, and the structure factors S(q). Information theory provides a measure, $h_{\mu }$, of the randomness or unpredictability of a system and also provides measures of the apparent spatial memory of a configuration, the excess entropy E and the coarser two-spin mutual information I(r).

However, information theory tells us little about how a system utilizes its memory nor whether the apparent memory (E) is equal to the minimum amount of memory ($C_{\mu }$) actually required internally to produce configurations. To help address this concern, computational mechanics was put forth as a way to discover and quantify the intrinsic computational capability of a system. By constructing a model (an ϵ-machine) that statistically reproduces the system’s configurations, we obtain an explicit description of the architecture of the minimal information processing apparatus needed to produce the configuration ensemble. One consequence is that the statistical-mechanical and information-theoretic quantities can be calculated directly.

Let’s now return to the main theme: discovering structure and quantifying patterns in spin systems. Do ϵ-machines capture our intuitive notion of pattern? If so, in what sense? Furthermore, how is the architectural analysis of information processing provided by computational mechanics related to the notion of pattern? It is not obvious a priori that examining the intrinsic computation of a system is a sensible approach to describing patterns. However, we will demonstrate below that ϵ-machines provide a more explicit representation of all the patterns, symmetries, and regularities in a spin configuration than is provided by either information theory or statistical mechanics. To do so, we shall calculate statistical-mechanical, information-theoretic, and computation-mechanical quantities for several short-range one-dimensional Ising systems. In the next section we report our calculational techniques more thoroughly than in Ref. [39]. We discuss our results for models with nearest and next-nearest neighbor interactions and then proceed to a direct comparison of the three different approaches to discovering and quantifying patterns.

## 5. Computational Mechanics of One-Dimensional Spin Systems

### 5.1. Calculational Methods

As is well known, the partition function *Z* for any one-dimensional spin system with finite-range interactions can be expressed in terms of the transfer matrix *V* [121]. Namely, $Z=\mathrm{Tr}\;V^{N}$, where $V^{N}$ is the $N^{\mathrm{th}}$ power of *V* and *N* is proportional to the system size. The transfer matrix may be viewed as a function of the values of blocks of consecutive spins, with the required block size depending on the interaction range. The dimensionality of the transfer matrix is chosen to be large enough so that the sum over all spin configurations, as in Equation (7), can be re-expressed as a product of transfer matrices. Hence, the transfer matrix approach effectively decomposes a configuration into a concatenation of contiguous spin blocks.

For the simple case of a spin-1/2 nearest neighbor (nn) Ising system a spin block length of 1 suffices and so *V* is a 2 × 2 matrix defined by

(81) $$V\left(s_{i},s_{i+1}\right)\equiv \exp\left[J_{1}\beta s_{i}s_{i+1}+\frac{1}{2}B\beta \left(s_{i}+s_{i+1}\right)\right]\;.$$

Here, the row and column indices correspond to the values of a single spin and its nearest neighbor to the right, respectively.

For *R*-range interactions the spins must be grouped into blocks of *R* consecutive spins. For a spin-*K* system, the row and column indices then run over the ${\left(2K+1\right)}^{R}$ possible values the spins can assume in a block of *R* sites. We shall denote the individual ${\left(2K+1\right)}^{R}$ possible values of an *R*-spin block by η. Only for the special case of a nearest neighbor (R=1) interaction does η=s, a single spin. Subscripts on the spin blocks indicate lattice site, not the particular value of the spin block. The transfer matrix connecting the $i^{\mathrm{th}}$ and ${\left(i+1\right)}^{\mathrm{st}}$ spin blocks is denoted by $V(\eta_{i},\eta_{i+1})$.

For a given system there are a number of ways to construct a transfer matrix that describes its statistical mechanics; see, e.g., Ref. [122]. A general method is elegantly described by Dobson in Ref. [123]. Below, we shall assume that *V* has been constructed to add on the effects of *R* spins per matrix operation, where *R* is again the system’s interaction range. By $u^{R}$ ($u^{L}$) we denote the right (left) eigenvector corresponding to *V*’s largest eigenvalue λ, normalized so that the inner product of $u^{R}$ and $u^{L}$ is unity. As is well known, transfer matrices for finite-range one-dimensional spin systems with finite-strength interactions possess a positive, unique largest eigenvalue.

#### 5.1.1. Determination of Recurrent Causal States

Simply stated, our goal is to go from the transfer matrix description that maps *R*-blocks to contiguous *R*-blocks to the ϵ-machine describing a stochastic process that maps site values to site values as one moves along the configuration. Said in the terminology of coding theory and symbolic dynamics, the task is to go from a contiguous-block code to a sliding-block code [124].

Our first step, taken in this section, is to find the recurrent causal states from a range-*R* spin system determined by *V*. The subsequent section determines the causal state transition probabilities. The intervening calculations, though straightforward, are a bit tedious. The reader may wish to skip to the section’s end where the results are summarized.

To find the causal states we need to form conditional probabilities as in Equation (53). In particular, we must find an expression for the probability that *L* consecutive spins take on the particular values $s_{i},s_{i+1},\cdots ,s_{i+(L-1)}$. For convenience, we let $L=RL^{\prime }$ where $L^{\prime }>0$ is an integer that indexes the contiguous *R*-blocks in the lattice; that is,

(82) $$\eta_{i}=s_{Ri}s_{Ri+1}\cdots s_{R(i+1)-1},\;i=0,1,\ldots ,L^{\prime }-1\;.$$

This choice does not affect the results, but simplifies the following derivations. After constructing a transfer matrix (e.g., Equation (81)), one can use the Boltzmann distribution of Equation (1) to obtain:

(83) $$\mathrm{Pr}\left(s_{0},s_{1},\cdots ,s_{L-1}\right)=\frac{u_{\eta_{L^{\prime }-1}}^{R}u_{\eta_{0}}^{L}}{\lambda^{L^{\prime }-1}}\prod_{i=1}^{L^{\prime }-2}V\left(\eta_{i},\eta_{i+1}\right)\;.$$

That is, for a given block of *L* spins, the probability is a product of components of the transfer matrix and its principal eigenvalue and eigenvectors. Each particular configuration $s_{0}s_{1}\cdots s_{L-1}$ specifies unique values of the contiguous *R*-spin blocks $\eta_{0},\eta_{1},\cdots ,\eta_{L^{\prime }-1}$ in the configuration. To evaluate the righthand side of Equation (83), the components of the matrices and vectors are chosen by the η variables that correspond to the particular spin variables on the left-hand side; that is, according to Equation (82).

Consider an infinite configuration split at $s_{0}$ and left- and right-half configurations of length *L* on either side:

(84) $$\overset{\leftarrow }{x}{}^{L}\equiv s_{-L}s_{-L+1}\ldots s_{-2}s_{-1}$$

and

(85) $$\vec{x}{}^{L}\equiv s_{0}s_{1}\ldots s_{L-2}s_{L-1}\;.$$

Now,

(86) $$\mathrm{Pr}\left(\vec{x}{}^{L}|\overset{\leftarrow }{x}{}^{L}\right)=\frac{\mathrm{Pr}(\vec{x}{}^{L},\overset{\leftarrow }{x}{}^{L})}{\mathrm{Pr}(\overset{\leftarrow }{x}{}^{L})}\;.$$

Using Equation (83), the definitions of $\overset{\leftarrow }{x}{}^{L}$ and $\vec{x}{}^{L}$, and Equations (84) and (85) in Equation (86) we have, after some simplifying,

(87) $$\mathrm{Pr}\left(\vec{x}{}^{L}|\overset{\leftarrow }{x}{}^{L}\right)=\frac{u_{\eta_{L^{\prime }-1}}^{R}}{\lambda^{L^{\prime }}u_{\eta_{-1}}^{R}}\prod_{i=-1}^{L^{\prime }-2}V\left(\eta_{i},\eta_{i+1}\right)\;.$$

Recall that we view this as a function over all possible length-*L* right-half configurations $\vec{x}{}^{L}$ conditioned on a particular length-*L* left-half configuration $\overset{\leftarrow }{x}{}^{L}$. Analyzing this equation is the key step in determining the causal states.

Notice that Equation (87) indicates that of all the spin blocks in $\overset{\leftarrow }{x}{}^{L}=\eta_{-L^{\prime }},\ldots ,\eta_{-1}$, $\mathrm{Pr}(\vec{x}{}^{L}|\overset{\leftarrow }{x}{}^{L})$ only depends on the single spin block $\eta_{-1}$. All the other spin blocks η in Equation (87) are members of $\vec{x}{}^{L}$. That is, the probability distribution over right-half configurations depends only on the value of the left-most (closest) neighboring block. This result holds for any L>R. Hence,

(88) $$\mathrm{Pr}\left(\vec{x}{}^{L}|\overset{\leftarrow }{x}{}^{L}\right)=\mathrm{Pr}\left(\vec{x}{}^{L}|\eta_{-1}\right)\;.$$

Expressed informally, the values of $s_{0},\ldots ,s_{L-1}$ are “shielded” from $s_{-L},\ldots ,s_{-R-1}$ by spin block $\eta_{-1}=s_{-R}\cdots s_{-1}$, $\vec{x}{}^{L}$’s leftmost *R* neighboring spins. This observation was made in a different context by Baker [125].

This somewhat surprising result can be explained physically as a direct consequence of the range-*R* interactions in the Hamiltonian. The probability of a right-half configuration $X_{0}$ depends only on its energy. Of all the spins in $\overset{\leftarrow }{x}{}^{L}$, only the spins in the $\eta_{-1}$ block—i.e., the block that neighbors $X_{0}$—contributes to the energy and hence to the probability of $X_{0}$.

Recall that two left-half configurations are considered equivalent if and only if they give rise to the same distribution of right-half configurations conditioned on having seen those particular left halves. The equivalence classes induced by this relation are identified as the causal states. Thus, Equation (87) tells us that for a spin-*K* system with range *R* interactions there are at most ${\left(2K+1\right)}^{R}$ recurrent causal states corresponding to the ${\left(2K+1\right)}^{R}$ possible values of a single spin block: $|S^{\left(R\right)}{|\leq \left(2K+1\right)}^{R}$. Recall that the recurrent causal states are the equivalence classes in the infinite-sequence limit in Equation (53). In determining the causal states, say by successively increasing *L* from 0, Equation (87) shows us that the set of causal states will not change once L>R. For any L>R, the conditional distribution of Equation (87) depends only on $\eta_{-1}$, as indicated by Equation (88).

To complete our determination of the recurrent causal states, we must make sure that each different value of $\eta_{-1}$ actually gives rise to a *different* $\mathrm{Pr}(\vec{x}{}^{L}|\eta_{-1})$. That is, we must check for all different spin-block pairs, $\eta_{-1}\neq \eta_{-1}^{\prime }$, that

(89) $$\mathrm{Pr}\left(\vec{x}{}^{L}|\eta_{-1}\right)\neq \mathrm{Pr}\left(\vec{x}{}^{L}|\eta_{-1}^{\prime }\right)\;,$$

for at least one $\vec{x}{}^{L}\in A^{L}$. Note that $\vec{x}{}^{L}$ can be decomposed into a telescoping product over its $\eta_{i}$’s; that is,

(90) $$\mathrm{Pr}\left(\vec{x}{}^{L}|\eta_{-1}\right)=\prod_{i=-1}^{L^{\prime }-2}\mathrm{Pr}\left(\eta_{i+1}|\eta_{i}\right)\;.$$

Looking at the future conditional probability distribution, Equation (87), we see that Equation (89) may be written as

(91) $$\frac{V(\eta_{-1},\eta_{0})}{u_{\eta_{-1}}^{R}}\neq \frac{V(\eta_{-1}^{\prime },\eta_{0})}{u_{\eta_{-1}^{\prime }}^{R}}\;,$$

for at least one $\eta_{0}$, the next spin-block.

It should be emphasized that we are fixing two particular values for the rightmost spin block in the left half, $\eta_{-1}$ and $\eta_{-1}^{\prime }$, and comparing the distribution over all possible values of $\vec{x}{}^{L}$ or its surrogate block $\eta_{0}$. When $\eta_{-1}\neq \eta_{-1}^{\prime }$, if Equation (91) holds for at least one $\eta_{0}$, then the conditional distributions are distinct. This, in turn, means that the causal states are in a one-to-one relation with the values of *R*-spin blocks. If this is not the case, then we have found two distinct blocks, $\eta_{-1}$ and $\eta_{-1}^{\prime }$, that lead to the same conditional distribution $\mathrm{Pr}(\vec{x}{}^{L}|•)$. Therefore, (i) $\eta_{-1}$ and $\eta_{-1}^{\prime }$ are in the same equivalence class and (ii) there are fewer recurrent causal states than there are spin blocks. For the Ising systems considered here, condition Equation (91) is almost always met; if system parameters are randomly chosen, Equation (91) holds with probability 1. The immediate conclusion is that the set of *R*-spin blocks {η} is the set of recurrent causal states. The exceptions, though, are notable, as we will see later on.

We can simplify the notation of Equation (91) by dropping the *R*-block index when referring to the transfer matrices and its eigenvectors. We can then express Equation (91) in terms of the components of the transfer matrix and the eigenvectors. That is, we change

(92) $$V\left(\eta_{-1},\eta_{0}\right)\to V_{ij}\;,$$

where $i,j=0,1,\ldots ,{\left(2K+1\right)}^{R}-1$ index the rows and columns of *V* and correspond to the values of $\eta_{-1}$ and $\eta_{0}$, respectively. Using this simpler notation, the condition for distinct conditional distributions Equation (91) may be rewritten as

(93) $$V_{ik}/u_{i}^{R}\neq V_{jk}/u_{j}^{R}\;,$$

for at least one *k*. If this is satisfied, then the recurrent causal state probabilities are given by:

(94) $$\mathrm{Pr}\left(S_{i}^{\left(R\right)}\right)=u_{i}^{R}u_{i}^{L}\;,$$

for $i=0,1,\ldots ,{\left(2K+1\right)}^{R}-1$. If Equation (93) is not satisfied, however, then the upper range of *i* will be less than ${\left(2K+1\right)}^{R}-1$ and there will be fewer causal states than *R*-spin blocks.

We have now determined an upper bound on $C_{0}$ and $C_{\mu }$ for a spin-*K* system with $R^{\mathrm{th}}$ nearest-neighbor interactions: $C_{\mu }\leq C_{0}\leq R\log_{2}\left(2K+1\right)$. This result indicates that this class of spin systems is a severely restricted subset of ϵ-machines. For example, the number of causal states is finite for all parameter values.

#### 5.1.2. Causal State Transitions

Now that we have found the recurrent causal states, our task is to determine the probabilities for transitions between them: $T_{\alpha \beta }^{\left(s\right)}=\mathrm{Pr}\left(S_{\beta },s|S_{\alpha }\right)$, where the indices run over only the recurrent causal states.

Recall that transitions between causal states are *deterministic* in the sense that knowledge of the next spin determines the next causal state; that is, $\mathrm{Pr}(S_{\beta }|s,S_{\alpha })=1$, if the transition is allowed and zero otherwise. Thus, it suffices to know $\mathrm{Pr}(s|S_{\alpha })$ to determine $T_{\alpha \beta }^{\left(s\right)}$.

It follows from Equation (83) that

(95) $$\mathrm{Pr}\left(\eta_{0}|\eta_{-1}\right)=\frac{1}{\lambda }V\left(\eta_{-1},\eta_{0}\right)\frac{u_{\eta_{0}}^{R}}{u_{\eta_{-1}}^{R}}\;,$$

where $\eta_{0}=s_{0}s_{1}\cdots s_{R-1}$ is the contiguous, nonoverlapping *R*-spin block to the right of $\eta_{-1}=s_{-R}s_{-R+1}\cdots s_{-1}$. To obtain $\mathrm{Pr}(s_{0}|\eta_{-1})$ from $\mathrm{Pr}(\eta_{0}|\eta_{-1})$ we must sum over all the spin variables in $\eta_{0}$ except for $s_{0}$. Hence:

(96) $$\mathrm{Pr}\left(s_{0}|\eta_{-1}\right)=\sum_{s_{1}}\cdots \sum_{s_{R-1}}\frac{1}{\lambda }V\left(\eta_{-1},\eta_{0}\right)\frac{u_{\eta_{0}}^{R}}{u_{\eta_{-1}}^{R}}\;.$$

For a next nearest-neighbor (R=2) system we have, for example,

(97) $$\begin{array}{cc} \; & \mathrm{Pr}(↑|\eta_{-1})=\mathrm{Pr}(↑↓|\eta_{-1})+\mathrm{Pr}(↑↑|\eta_{-1})\\ \; & \;\;=\frac{1}{\lambda }V(\eta_{-1},↑↓)\frac{u_{↑↓}^{R}}{u_{\eta_{-1}}^{R}}+\frac{1}{\lambda }V(\eta_{-1},↑↑)\frac{u_{↑↑}^{R}}{u_{\eta_{-1}}^{R}}\;. \end{array}$$

$T_{\alpha \beta }^{\left(s\right)}$ follows immediately from Equation (96).

The transient states and their transition probabilities can be determined as in Appendix B. Taken altogether, then, the recurrent and transient states plus their transition probabilities constitute an ϵ-machine for the spin system described by the transfer matrix *V*. We shall give examples of spin system ϵ-machines in the following sections.

#### 5.1.3. Spin System Statistical Complexity

A previous section identified the recurrent causal states as the possible values of the spin blocks η, assuming Equation (93) is satisfied. Recalling that the statistical complexity is the Shannon entropy over the asymptotic causal state distribution, we may use Equations (69) and (94) to obtain:

(98) $$\begin{array}{c} C_{\mu }=-\;\;\sum_{i=0}^{|S^{\left(R\right)}|-1}\;\;u_{i}^{R}u_{i}^{L}\log_{2}\left(u_{i}^{R}u_{i}^{L}\right)\;. \end{array}$$

Equation (98) is equivalent to setting L=R in Equation (33),

(99) $$C_{\mu }=H\left(R\right)\;.$$

That is, the statistical complexity is the Shannon entropy H(R) of the *R*-spin block distribution. As already noted, H(R), divided by *R* to give a density, is not the entropy density $h_{\mu }$, even if R=1.

#### 5.1.4. Spin System Entropy Density

Since the probability of a spin block depends only on the value of that block’s nearest neighbor, Equation (36) for the entropy density reduces to

(100) $$h_{\mu }=\frac{1}{R}H\left[\eta_{i}|\eta_{i-1}\right]\;,$$

a form for $h_{\mu }$ discussed in Appendix C. Using Equation (83) we find that

(101) $$h_{\mu }=\frac{1}{R}\left(\log_{2}\lambda -\lambda^{-1}\;\;\;\;\sum_{i,j=0}^{|S^{\left(R\right)}|-1}\;\;\;\;u_{i}^{R}u_{j}^{L}V_{ji}\log_{2}\left[V_{ji}\right]\right).$$

Although not apparent, it is straightforward to show that, for systems described by a finite-dimensional transfer matrix, Equation (101) is equivalent to the more familiar expression for entropy density,

(102) $$h_{\mu }=-\lim_{N\to \infty }\frac{\partial }{\partial T}\left(-\frac{T}{N}\log Z\right)\;,$$

using $Z=\mathrm{Tr}\;V^{N}$. Our method for calculating the entropy density by using Equation (36) has also been used by Lindgren [76].

#### 5.1.5. Spin System Excess Entropy

The range-*R* interactions also lead to a compact expression for the excess entropy. Recall that E may be expressed as the mutual information between the left and right halves of a configuration. Since only the neighboring *R*-spin block of one half influences the distribution over the other half, it follows that:

(103) $$\begin{array}{cc} E & =I\left[\overset{\leftarrow }{S};\vec{S}\right]=I\left[\eta_{i};\eta_{i+1}\right]=I\left[S_{0};S_{R}\right]\;, \end{array}$$

where $S_{0},S_{1},\ldots ,S_{R}$ is a spatial sequence of recurrent causal states, so that $S_{R}$ denotes the causal state seen *R* spins after seeing $S_{0}$.

Calculating E using Equation (103) requires the marginal distribution of S and the joint distribution of $S_{0}$ and $S_{R}$. The former is simply the asymptotic distribution over causal states Pr(S), as given by Equation (94). The joint distribution follows by applying the stochastic connection matrix T:

(104) $$\begin{array}{ccc} \mathrm{Pr}(S_{0},S_{R}) & = & \sum_{S_{1},\cdots ,S_{R-1}}\mathrm{Pr}\left(S_{0}\right)T_{S_{0}S_{1}}T_{S_{1}S_{2}}\cdots T_{S_{R-1}S_{R}}=\mathrm{Pr}\left(S_{0}\right)T_{S_{0}S_{R}}^{R-1}\;, \end{array}$$

where $T^{R-1}$ is the ${\left(R-1\right)}^{\mathrm{th}}$ power of T. From Equations (94), (103) and (104), E follows readily. We should emphasize, however, that Equation (103) is not completely general—it applies only to *R*-range one-dimensional spin systems for which Equation (93) holds.

In terms of the transfer matrix, an expression for E follows by inserting Equation (83) into Equation (103) and simplifying;

(105) $$\begin{array}{ccc} E & = & -\log_{2}\lambda +\lambda^{-1}\sum_{i,j=0}^{|S^{\left(R\right)}|-1}u_{i}^{R}u_{j}^{L}V_{ji}\log_{2}\left[V_{ji}\right]-\sum_{j=0}^{|S^{\left(R\right)}|-1}u_{j}^{R}u_{j}^{L}\log_{2}\left[u_{j}^{R}u_{j}^{L}\right]\;. \end{array}$$

One can also calculate E and $h_{\mu }$ by determining an expression for H(L) in terms of *V* and using Equation (42). Doing this, we get formulae that agree with those derived above.

#### 5.1.6. Relationships between Spin System Memory Measures

Note that these results—Equations (98), (101) and (105)—establish an explicit version of the inequality in Equation (80) between E and $C_{\mu }$ mentioned above; namely:

(106) $$C_{\mu }=E+Rh_{\mu }\;,$$

where *R* is the range of interaction and again assuming that Equation (93) is satisfied. Equation (106) is a consequence of the information theoretic identity:

(107) $$H\left[\eta_{i}\right]=I\left[\eta_{i};\eta_{i+1}\right]+H\left[\eta_{i+1}|\eta_{i}\right]\;.$$

This result, Equation (106), also applies to finite-step Markov chains in which blocks over the observed alphabet A are in 1−1 correspondence with the internal-state blocks $S^{R}$. Note that the fair coin and the noisy period-2 examples violate this condition and so Equation (106) does not hold for them. (See Table 1.)

Equation (106) shows that E, $C_{\mu }$, and $h_{\mu }$ are not independent for the finite-range systems considered here. As such, we will focus mostly on E and $h_{\mu }$ for the remainder. That is, when discussing ϵ-machines below, we will consider mainly their detailed structure and will not focus on the single number $C_{\mu }$.

Finally, since mutual information, and thus E, is a nonnegative quantity, we note that:

(108) $$C_{\mu }\geq h_{\mu }\;,$$

recalling the restrictions on Equation (106). It might seem puzzling that the amount of information carried by the ϵ-machine—“Which causal state is the process in?”—is *larger* than the information available (on average) from individual spin observations. However, $C_{\mu }$ and $h_{\mu }$ simply measure different types of information.

### 5.2. Spin-1/2 Nearest-Neighbor Systems

Starting with the transfer matrix, the preceding section developed a general method for determining the causal states, constructing an ϵ-machine, and calculating the statistical complexity, entropy density, and excess entropy. The results describe all finite-range one-dimensional spin systems. This section applies these results to the simple case of nearest-neighbor (nn) spin-1/2 systems; those with $J_{r}=J_{1}\delta_{r1}$ in Equation (6). The main goal is to illustrate the use of our methods and to allow the reader to gain familiarity with the quantities defined in earlier sections. Subsequent sections consider longer range models, compare and contrast E and ϵ-machines with the measures of structure found in statistical mechanics and, then, draw general conclusions about the behavior of these different quantities.

#### 5.2.1. ϵ-Machines for the Spin-1/2, Nearest-Neighbor Ising Model

**Figure 8:**
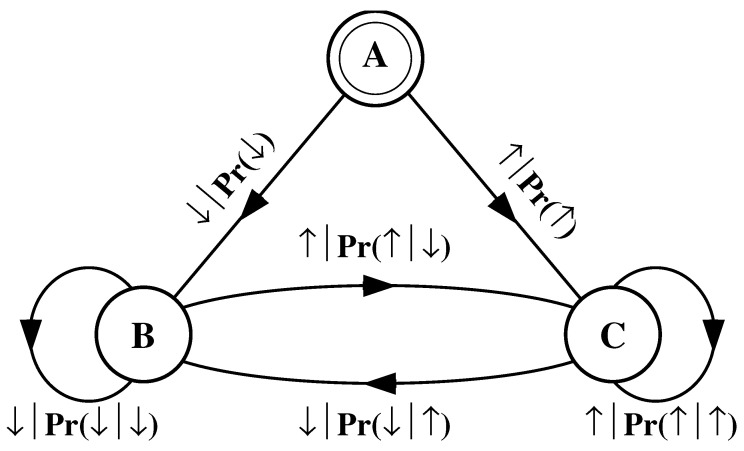
The spin-1/2 Ising ϵ-machine. The double-circled causal state A is the start state. It is a transient state, never visited again after the first transition. The two initial transitions give the probabilities of isolated up and down spins.

For the special case of a spin-1/2 system with nearest-neighbor interactions and, for those parameter values where Equation (93) holds, the corresponding ϵ-machine is shown in Figure 8. The transition probabilities are obtained from Equation (96) and the transient state construction technique of Appendix B. State A is the start state and is the only transient state. States B and C are recurrent.

The transition matrices $\left\{T^{\left(s\right)}:s\in A\right\}$ are given by:

(109) $$T^{\left(↓\right)}=\left(\begin{array}{ccc} 0 & \mathrm{Pr}(↓) & 0\\ 0 & \mathrm{Pr}(↓|↓) & 0\\ 0 & \mathrm{Pr}(↓|↑) & 0 \end{array}\right)$$

and

(110) $$T^{\left(↑\right)}=\left(\begin{array}{ccc} 0 & 0 & \mathrm{Pr}(↑)\\ 0 & 0 & \mathrm{Pr}(↑|↓)\\ 0 & 0 & \mathrm{Pr}(↑|↑) \end{array}\right)\;.$$

The stochastic connection matrix T, being the sum of the above two matrices, is:

(111) $$T=\left(\begin{array}{ccc} 0 & \mathrm{Pr}(↓) & \mathrm{Pr}(↑)\\ 0 & \mathrm{Pr}(↓|↓) & \mathrm{Pr}(↑|↓)\\ 0 & \mathrm{Pr}(↓|↑) & \mathrm{Pr}(↑|↑) \end{array}\right)\;.$$

The components $T_{\alpha \beta }$ give the probability of making a transition from causal state α to causal state β. As before, we use the convention that the numerical values of the index α correspond to the alphabetical indices of the causal states in the natural way; α=0 corresponds to causal state A, α=1 to B, etc. For example, the probability of making a transition from B to C is given by $T_{12}^{\left(↑\right)}=T_{12}=\mathrm{Pr}\left(↑|↓\right)$. The matrices given by Equations (109) and (110) are equivalent to the ϵ-machine shown in Figure 8.

In terms of the spin system parameters $J_{1}$, *B*, and *T*, the elements of the stochastic connection matrix can be calculated explicitly via Equation (96) and the transient state construction technique of Appendix B. We find:

(112) $$T=\left(\begin{array}{ccc} 0 & \frac{1+m}{2} & \frac{1-m}{2}\\ 0 & \kappa^{-1}e^{\frac{-B+J_{1}}{T}} & 1-\kappa^{-1}e^{\frac{-B+J_{1}}{T}}\\ 0 & 1-\kappa^{-1}e^{\frac{B+J_{1}}{T}} & \kappa^{-1}e^{\frac{B+J_{1}}{T}} \end{array}\right)\;.$$

The normalization factor κ is given by:

(113) $$\kappa =e^{\frac{J_{1}}{T}}\cosh\left(\frac{B}{T}\right)+\sqrt{e^{\frac{-2J_{1}}{T}}+e^{\frac{2J_{1}}{T}}\sinh^{2}\left(\frac{B}{T}\right)}\;,$$

and *m*, the magnetization, is:

(114) $$m=\frac{e^{\frac{J_{1}}{T}}\sinh\left(\frac{B}{T}\right)}{\sqrt{e^{\frac{-2J_{1}}{T}}+e^{\frac{2J_{1}}{T}}\sinh^{2}\left(\frac{B}{T}\right)}}\;.$$

#### 5.2.2. Paramagnet

We now apply these results to several particular cases, beginning with paramagnetic coupling, $J_{1}=0$. It is easy to check that Equation (93) is not satisfied. Hence, there is only one causal state and $C_{\mu }=0$ for all temperatures and all values of the external field *B*. Physically, this is because at $J_{1}=0$ there is no coupling at all between the spins; a spin exerts no influence on the value of its neighbors. As a result, there is only one conditional distribution:

(115) $$\mathrm{Pr}(\vec{x}{}^{L}|s_{-1}=\;↓)\;=\;\mathrm{Pr}(\vec{x}{}^{L}|s_{-1}=\;↑)\;.$$

**Figure 9:**
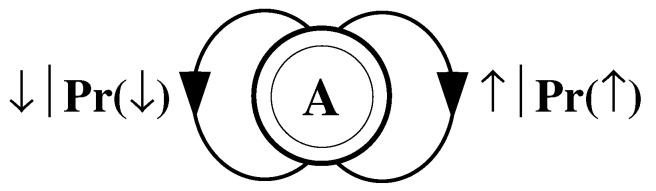
The ϵ-machine for a paramagnet. Pr(↑) and Pr(↓) depend on *B* and *T*. However, $C_{\mu }=E=0$, for T>0.

The ϵ-machine for the paramagnet is shown in Figure 9. In terms of *B* and *T*:

(116) $$\mathrm{Pr}\left(↑\right)=\left(1/2\right)\frac{e^{B/T}}{\cosh(B/T)}\;,$$

and Pr(↓)=1−Pr(↑). The start state is recurrent for this particularly simple process. If there is no external field *B* to bias the spins, then Pr(↓)=Pr(↑)=1/2 and the ϵ-machine of Figure 9 is identical to the the fair coin machine of Figure 3.

Since knowledge of a spin carries no information about the value of its neighbors, the excess entropy also vanishes for the paramagnet. If there is no external field *B* to bias the spins, all configurations are equally likely and $h_{\mu }=1$. As *B* increases from 0, the configurations are biased toward ↑, and the entropy density monotonically decreases. Note that for |B|<∞ and T>0, $h_{\mu }$ can take on all possible values except for 0; $0<h_{\mu }\leq 1$. Yet for all *B*, $C_{\mu }=E=0$. This simple example illustrates how excess entropy and statistical complexity are measuring a property that is clearly distinct from the entropy density—they are different from randomness. We can also see how the process of determining the causal states factors out the randomness in the system.

#### 5.2.3. Ferromagnetic Coupling

**Figure 10:**
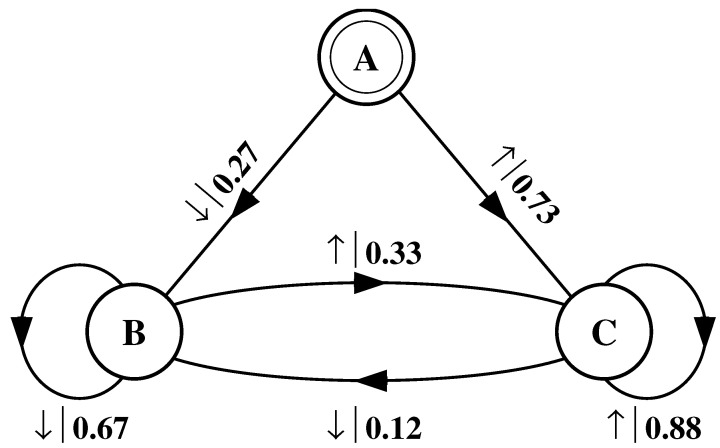
A typical ϵ-machine for ferromagnetic coupling: $J_{1}=1.0$, B=0.3, and T=1.50. $C_{\mu }=0.72$ bits, E=0.16 bits, and $h_{\mu }=0.56$ bits per site. Note the high probability of “self-transitions” from B to B and from C to C, a manifestation of the relatively large ferromagnetic interaction.

For ferromagnetic coupling, $J_{1}>0$, Equation (93) holds for all temperatures except zero and infinity. In this range the recurrent causal states may be identified with the values of a single spin. An ϵ-machine for typical parameter values is shown in Figure 10.

At infinite temperature, thermalization dominates and the spins effectively decouple; all configurations are equally likely. Thus, as for paramagnet, there is only one conditional distribution,

(117) $$\mathrm{Pr}(\vec{x}{}^{L}|s_{-1}=↓)=\mathrm{Pr}(\vec{x}{}^{L}|s_{-1}=↑)\;.$$

**Figure 11:**
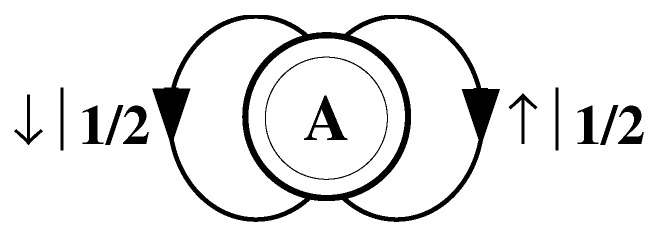
The ϵ-machine for T=∞. There is no memory, of any type: $C_{\mu }=E=0$ bits. The infinite temperature machine is identical for the ferro-, para-, and antiferromagnetic couplings so long as $J_{1}$ and *B* remain finite.

As a result, there is only one causal state, and $C_{\mu }$ and E vanish. The ϵ-machine for infinite temperature ferromagnetic coupling, shown in Figure 11, is identical to the fair coin machine, Figure 3.

**Figure 12:**
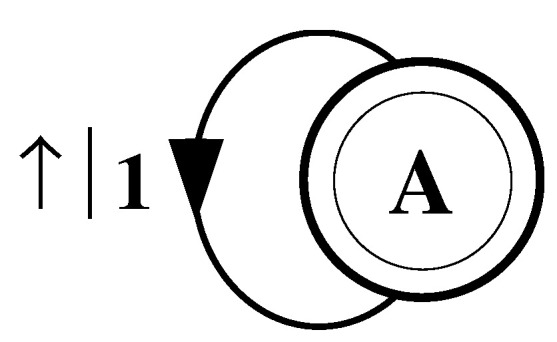
The ϵ-machine for the ferromagnetic ground state, T=0. $C_{\mu }=E=0$ bits for 0<B<∞ and $0<J_{1}$.

At zero temperature there are no thermal fluctuations and the spins are locked in their ferromagnetic ground state: all spins align with the external field. This situation is exactly the same as the period-1 example considered in Section 4.1.2. Thus, as shown in Figure 12, there is only one causal state and only one transition. The excess entropy, statistical complexity, and entropy density all vanish for this simple, trivially predictable system.

**Figure 13:**
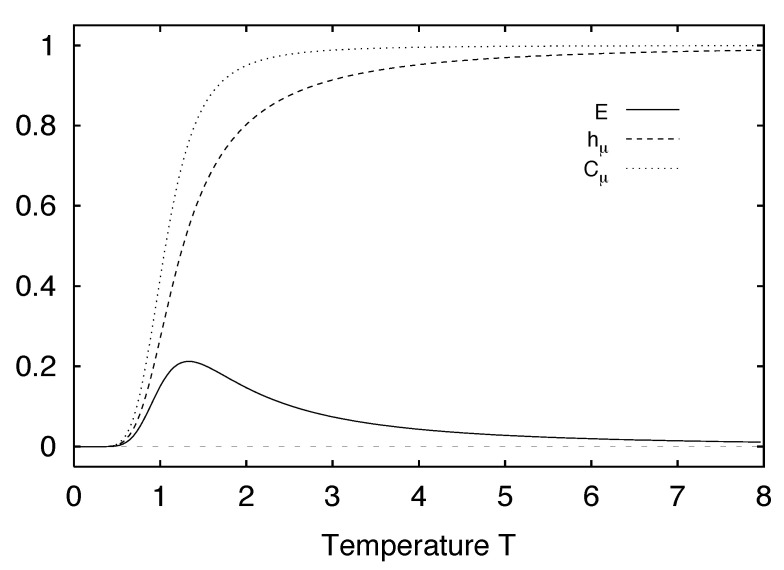
$C_{\mu }$, $h_{\mu }$, and E as a function of *T* for nn spin-1/2 ferromagnetic coupling. *B* was held at 0.20 and $J_{1}=1$.

The statistical complexity and excess entropy for nearest-neighbor ferromagnetic coupling are plotted as a function of temperature in Figure 13. $C_{\mu }$ increases monotonically as a function of temperature until T=∞ (not shown). There, as mentioned above, the couplings between spins become negligible, causing the two causal states to merge into one, yielding $C_{\mu }=0$. The monotonic increase in between these two extremes is due to the distribution over the two causal states, B and C, becoming more uniform as the temperature is increased. Since $C_{\mu }$ is the Shannon entropy of the causal state distribution, it is maximized when the distribution is uniform. This distribution is approached as one nears, but is not at, T=∞.

**Figure 14:**
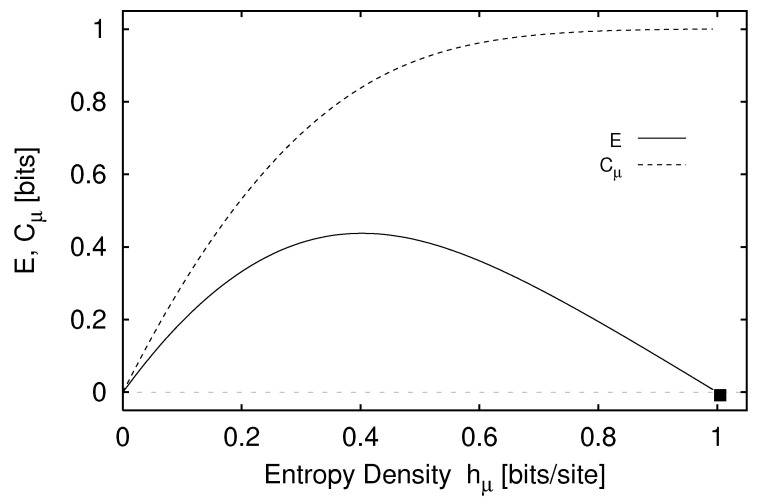
The complexity-entropy diagram for ferromagnetic coupling; $C_{\mu }$ and E plotted parametrically against $h_{\mu }$. The coupling constant and field were fixed—$J_{1}=1.0$ and B=0.05—as *T* was varied. At $h_{\mu }=1$ (T=∞), $C_{\mu }=0$ bits; this is denoted by the square token.

In Figure 14 we plot $C_{\mu }$ and E parametrically as a function of the randomness, as measured by $h_{\mu }$. This plot is referred to as the complexity-entropy diagram [25]. The benefit of this type of plot is that it is free of the external control parameters—temperature, coupling strength, and external field. Thus, the complexity-entropy diagram gives direct access to a system’s information processing capabilities and provides a common set of coordinates with which to compare the information processing properties of systems with different architectures and control parameters. For example, in Ref. [39] we used the complexity-entropy diagram to compare the configurations generated by one-dimensional Ising systems with the sequences generated by the symbolic dynamics of the logistic map.

For ferromagnetic coupling, we see in Figure 13 and Figure 14 that E has a maximum in a region between total randomness ($h_{\mu }=1$) and complete order ($h_{\mu }=0$). At low temperatures (and, hence, low $h_{\mu }$) most of the spins align with the magnetic field. At high temperatures, thermal noise dominates and the configurations are quite random. In both regimes one half of a configuration contains very little information about the other half. For low $h_{\mu }$, the spins are fixed and so there is no information to share. For high $h_{\mu }$, there is much information at each site; a roughly equal number of spins point up and down, so the single spin uncertainty is quite high. However, this information is uncorrelated with all other sites. Thus, the excess entropy is small in these temperature regimes. In between the extremes, however, E has a single maximum at the temperature where spin coupling strength balances the thermalization. The result is a maximum in the system’s apparent spatial memory.

#### 5.2.4. Antiferromagnetic Coupling

**Figure 15:**
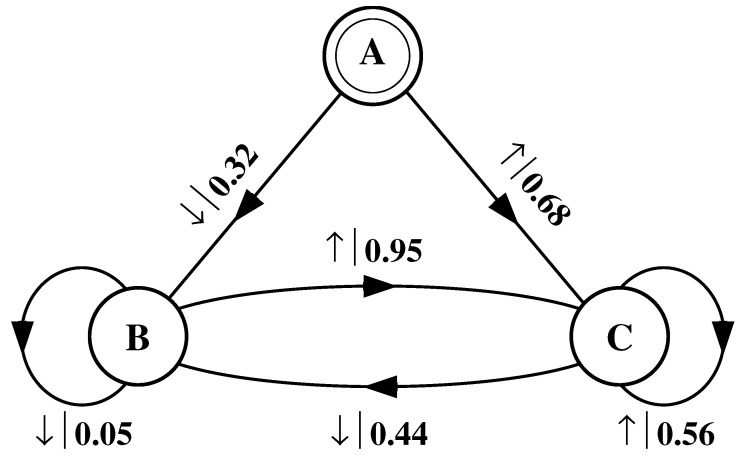
A typical ϵ-machine for antiferromagnetic (AFM) coupling. $J_{1}=-1.0$, B=1.8, and T=1.5. Giving $C_{\mu }=0.92$ bits, E=0.27 bits, and $h_{\mu }=0.65$ bits per site. Note the relatively strong interstate coupling.

An ϵ-machine for a typical antiferromagnetic (AFM) coupling ($J_{1}<0$) is shown in Figure 15. Note that “topologically” the AFM machine is identical to the FM machine of Figure 10—the states and their connectivity are identical. The difference between the two systems lies in the transition probabilities. The FM shows a high probability for self-transitions; Pr(B→B)=0.67 and Pr(C→C)=0.88. These self-loops are responsible for the FM pattern: aligned spins. For the AFM, the self-loops are relatively weaker; Pr(B→B)=0.05 and Pr(C→C)=0.56, with the high value of the latter being due partially to the high B(=1.8). This indicates a stronger tendency for spins to be anti-aligned, as expected for a system with AFM couplings.

**Figure 16:**
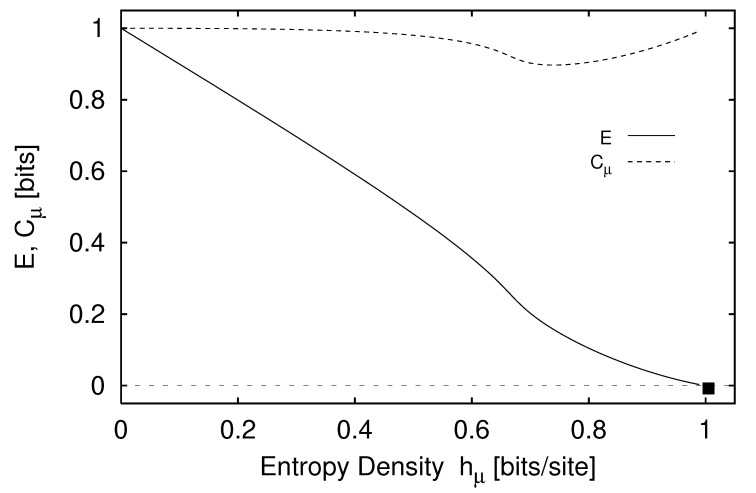
The complexity-entropy diagram for antiferromagnetic coupling. The temperature was varied as $J_{1}$ and *B* were held constant at $J_{1}=-1.0$ and B=1.80. As was the case for ferromagnet coupling, at $h_{\mu }=1$ (T=∞), $C_{\mu }=0$ bits; this is denoted by the square token.

The high-temperature behavior of the excess entropy is similar for both the AFM and FM. (See Figure 16.) Thermal fluctuations destroy all correlations and E vanishes. The low *T* behavior differs, however, as one might expect given the different ground states exhibited by models with ferro- and antiferromagnetic couplings: in the FM ground state, all the spins are aligned, while the AFM ground state consists of alternating up and down spins. The latter is, of course, the period-2 configuration given by Equation (51) that we considered back in Section 4.1 with Figure 6.

In Figure 16 we chose the antiferromagnetic coupling to be strong enough so that an antiferromagnetic ground state persists despite the presence of an external field. In the antiferromagnetic ground state the spatial configurations thus store one bit of information about whether the odd or even sites contain up spins. Accordingly, as can be seen in Figure 16, $E\to \log_{2}2=1$ bit as $h_{\mu }\to 0$.

#### 5.2.5. General Remarks

For different couplings and field strengths a range of E versus $h_{\mu }$ relationships can be realized, but their form is similar to those shown in Figure 14 and Figure 16; E either shows a single maximum or decreases monotonically. It is always the case, though, that E is bounded from above by $1-h_{\mu }$, which follows immediately if $C_{\mu }$ is set equal to its maximum value, 1 bit, in Equation (106).

We demonstrated this upper bound explicitly in Ref. [92] via a plot of the excess entropy versus entropy density for a nn Ising system with randomly chosen system parameters. Li [32] performed a similar study using several probabilistic automata.

In Section 4.5, $C_{\mu }$ was presented as a measure of structure. It is perhaps surprising, then, that it behaves so differently from E. As $h_{\mu }$ increases, one might expect $C_{\mu }$ to reach a maximum, as does E, and then decrease as the increased thermalization merges causal states that were distinct at lower temperatures. However, Figure 14 shows a monotonic increase in $C_{\mu }$ with $h_{\mu }$ for FM coupling. To understand this, recall that the number of recurrent causal states does not change as *T* is varied between zero and infinity. For the nn spin-1/2 Ising model, the number of causal states remains fixed at two. What *does* change as *T* is varied are the causal state probabilities Pr(S). For FM coupling, as the temperature rises the distribution Pr(S) becomes more uniform and so $C_{\mu }$ grows. This growth continues until *T* becomes infinite, since only there do the causal states collapse into one, at which point $C_{\mu }$ vanishes.

For AFM coupling the situation is a little different. At T=0 there are two recurrent causal states corresponding to the two spatial phases of the alternating up-down pattern. The probability of these causal states is equal. Hence, we see a low temperature statistical complexity of $C_{\mu }=1$ bit. At high (but finite) temperatures, the thermal fluctuations dominate; the anti-ferromagnetic order is lost, but the distribution over causal states is still relatively uniform, so the statistical complexity remains large. (As with FM coupling, at T=∞ the two causal states merge and $C_{\mu }$ falls to zero.) Between these extremes there is a region where the influence of the external field dominates, biasing the configurations. This is reflected in a bias in the causal state probabilities and $C_{\mu }$ dips below 1, as seen in Figure 16.

The tendency for $C_{\mu }$ to remain large for large values of $h_{\mu }$ is due to a more general effect, which follows from Equation (106): $C_{\mu }=E+Rh_{\mu }$. The memory needed to model a process (or for the process to produce its configurations) depends not only on the observed memory of the configurations generated, as measured by E, but also on its randomness, as measured by $h_{\mu }$. It is important to note, however, that $C_{\mu }$ is driven up by thermalization not because the model attempts to account for random spins in a configuration and not because the process must develop substantial memory resources to produce random spin values. Rather, $C_{\mu }$ rises with $h_{\mu }$ because Pr(S) becomes more uniform as the temperature increases. This reflects the fact that knowing in which causal state the process is becomes more informative in this regime.

### 5.3. Spin-1/2 Next-Nearest Neighbor Ising System

We now discuss the causal states and ϵ-machines for a spin system with nearest and next-nearest neighbor (nnn) interactions. That is, the coupling constants of Equation (6) now are given by

(118) $$J_{r}=J_{1}\delta_{1r}+J_{2}\delta_{2r}\;.$$

This system, capable of richer behavior than the nn system discussed above, is an important addition for discussing the detection of patterns and structure in subsequent sections. It will serve as our primary example when we compare computation-mechanical, statistical-mechanical, and information-theoretic approaches to structure.

**Figure 17:**
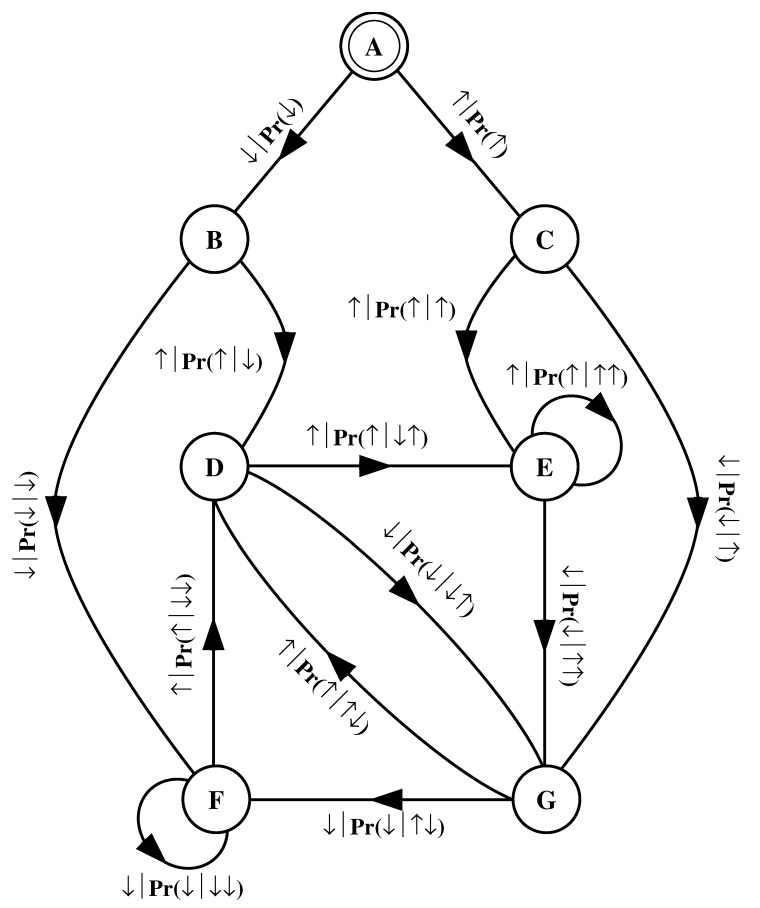
ϵ-machine for a next-nearest neighbor spin-1/2 Ising model. There are three transient states $S^{\left(T\right)}=\left\{A,B,C\right\}$ and four recurrent states $S^{\left(R\right)}=\left\{D,E,F,G\right\}$. The start state is A.

For the nnn system the recurrent causal states are, assuming Equation (93) is satisfied, in a one-to-one relation with the four possible values of a block of two spins. A general ϵ-machine for this system is shown in Figure 17. The connection matrix for this system is given by:

(119) $$T=\left(\begin{array}{ccccccc} 0 & \mathrm{Pr}(↓) & \mathrm{Pr}(↑) & 0\; & 0 & 0 & 0\\ 0 & 0 & 0 & \mathrm{Pr}(↑|↓)\; & 0 & \mathrm{Pr}(↓|↓) & 0\\ 0 & 0 & 0 & 0\; & \mathrm{Pr}(↑|↑) & 0 & \mathrm{Pr}(↓|↑)\\ 0 & 0 & 0 & 0 & \mathrm{Pr}(↑|↓↑) & 0 & \mathrm{Pr}(↓|↓↑)\\ 0 & 0 & 0 & 0 & \mathrm{Pr}(↑|↑↑) & 0 & \mathrm{Pr}(↓|↑↑)\\ 0 & 0 & 0 & \mathrm{Pr}(↑|↓↓) & 0 & \mathrm{Pr}(↓|↓↓) & 0\\ 0 & 0 & 0 & \mathrm{Pr}(↑|↑↓) & 0 & \mathrm{Pr}(↓|↑↓) & 0 \end{array}\right)\;.$$

There are several features to note about the ϵ-machine of Figure 17. First, the machine has more states than the nearest-neighbor system. This is a direct consequence of the longer-range interactions in the nnn system. Second, there are four recurrent causal states, D through G, and thus the topological complexity Equation (70) is $C_{0}=\log_{2}4=2$ bits. As noted in Section 4.5.1, the topological complexity sets an upper bound on the statistical complexity $C_{\mu }$; hence, $C_{\mu }\leq 2$ bits. Lastly, setting $h_{\mu }$ to its minimum value, 0, in Equation (106), we also obtain a bound on the excess entropy; E≤2 bits. We will discuss the behavior of E for the nnn system in the next section.

## 6. Excess Entropy Is a Wavelength-Independent Measure of Periodic Structure

Superficially, it might seem that the excess entropy and the structure factors reflect the same feature of a configuration. The excess entropy measures the total mutual information between two halves of a configuration and so may be viewed as the total apparent spatial memory stored in the configuration. The structure factors S(q), defined in Equation (21), are a sum over all two-point correlation functions and thus, in a limited sense, can be viewed as a measure of the total correlation. However, we shall see in this section that E and S(q) have several important differences.

**Figure 18:**
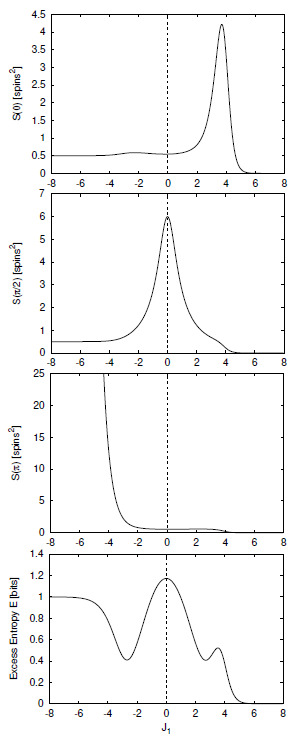
The structure factors S(0), S(π/2), and S(π) and the excess entropy E versus nearest-neighbor coupling $J_{1}$ for a next-to-nearest neighbor 1D Ising system. The parameters are B=0.05, T=1.0, and $J_{2}=-1.2$.

Figure 18 plots S(0), S(π/2), S(π), and E as a function of the coupling strength $J_{1}$ between nearest neighbors for the nnn Ising system just described. The field, temperature, and next-nearest neighbor coupling constant were held fixed at B=0.05, T=1.0, and $J_{2}=-1.2$. The structure factors of wavenumbers 0,π/2, and π correspond to wavelengths of spatial periods 1, 4, and 2, respectively.

Let’s first analyze the behavior of the structure factors in Figure 18. As $J_{1}$ goes to −∞, the thermal fluctuations become negligible and the system is confined to its ground state. The nnn coupling constant $J_{2}$ is also negligible in this limit. Hence, the system’s ground state is antiferromagnetic with period 2: alternating up and down spins and, in Figure 18, S(π) diverges.

For $J_{1}>0$ the nn coupling is ferromagnetic. As $J_{1}$ becomes larger than $J_{2}$, the system moves through a region of ferromagnetic structure similar to that reflected in Figure 13 and indicated by the S(0) peak in Figure 18. As $J_{1}\to +\infty$ the thermal fluctuations and the nnn coupling are again negligible and the system is fixed in its ground state. Here, since $J_{1}>0$, the ground state is ferromagnetic. All spins line up with the external field. As a result, $\langle s_{0}s_{r}\rangle ={\langle s\rangle }^{2}$ so all the Γ(r)’s vanish, yielding a vanishing S(0).

For $|J_{1}|\ll T$, the thermal fluctuations and the nnn coupling $J_{2}$ dominate and the lattice effectively decouples into two noninteracting chains. That is, the even and odd sites do not interact with each other. Since $J_{2}<0$, the ground state in this parameter regime is antiferromagnetic with period 4:

(120) ⋯↑↑↓↓↑↑↓↓↑↑↓↓↑↑↓↓↑↑⋯

As a result, we see a peak in S(π/2) at $J_{1}=0$ in Figure 18. The wavenumber π/2 corresponds to a period of 4. The structure factors S(0) and S(π) are insensitive to structure at this wavelength.

Figure 18 shows that the system exhibits significant structural changes, as indicated by the structure factors, as the parameter $J_{1}$ is varied. Notice, however, that analyzing the configurations using only one of the structure factors misses most of the changes that occur in the configurations elsewhere.

Excess entropy, however, is not limited to a particular wavelength. As can be seen in Figure 18, E picks up the ferromagnetic and both types of antiferromagnetic structure. This feature of the excess entropy is especially noteworthy, as statistical mechanics does not possess a structure factor that is applicable to all such situations.

The excess entropy is capable of detecting structure at any wavelength because it is a much more “global” function than the structure factors. Although calculation of the structure factor involves summing over all the variables in the chain, the correlations are considered in pairs, since the two-point correlation functions Γ(r) are summed over. Excess entropy, on the other hand, is defined as the information that the *entire* left half carries about the *entire* right half. The excess entropy treats the left half and the right half of a configuration as two (very large) composite variables; it does not break them into pairwise interactions. This is the sense in which we say that E is more global than S(q). Conversely, S(q) is somewhat “myopic”. By considering only two-point correlations modulated at some wavenumber *q*, S(q) misses structure that occurs at other wavenumbers and that is due to more-than-two-spin correlations.

The differences between E and S(q) can be better understood by considering the motivations behind their definition. Structure factors are designed to detect a pattern of a given periodicity. For example, if one performs a numerical experiment to determine the critical point of a paramagnet-antiferromagnet transition, then the antiferromagnetic structure factor S(π) is the natural quantity to use to detect the onset of antiferromagnetic ordering. If, however, one is interested in the apparent spatial memory of a configuration, E is the natural quantity to use.

Simply put, excess entropy and structure factors measure different things: E measures spatial memory and S(q) detects correlations of a particular periodicity. They behave similarly because the spatial memory of a configuration is relatively large if it has a periodic pattern. In fact, $E=\log_{2}P$ for a periodic configuration of period P. For the spin systems considered here, a configuration is periodic if and only if its entropy density $h_{\mu }$ vanishes. Thus, if a system has $h_{\mu }=0$, this implies that is periodic with $P=2^{E}$.

Since E and S(q) measure different properties of a configuration, they carry different units. The excess entropy is measured in bits while S(q) has the dimensions of spin-value squared. Note that E is a function of the distribution of the spin variables and, unlike S(q), does not depend on the values or units of the spin variables. For example, if we were to consider a model where $s_{i}\in \left\{\pm 2\right\}$ instead of $s_{i}\in \left\{\pm 1\right\}$, S(q) would increase by a factor of 4 while E would remain unchanged. This is a fairly trivial observation but, as has been mentioned elsewhere [75], it emphasizes how mutual information is a more flexible measure of correlation than that correlation function. In other words, “correlation” is best interpreted to be a statement concerning the joint distribution of two variables, not the values those variables can assume.

The fact that E and S(q) carry different units means that their numerical values are interpreted differently. This is particularly clear in the $J_{1}\to -\infty$ behavior of Figure 18. Here, S(π) diverges, indicating exact periodicity at q=π. The excess entropy, however, is finite: $E=\log_{2}2=1$ bit, indicating that the configurations store 1 bit of information.

Looking at Figure 18, it appears as if the sum of S(0), S(π/2), and S(π) might behave like E. Indeed, summing these three functions does produce a function that behaves like E for this particular system. However, summing up the relevant S(q)’s still depends on guessing the right *q*’s. A response to this objection might be to sum S(q) over *all q*’s. However, if one does this the different phases destructively interfere. As a result:

(121) $$\sum_{q=0}^{N-1}S_{N}\left(q\right)\equiv \;\sum_{q=0}^{N-1}\sum_{r=0}^{N-1}\Gamma \left(r\right)e^{2\pi irq/N}=\Gamma \left(0\right)\;.$$

All we’re left with is Γ(0), a “self-correlation” term that is a function of the distribution of a single spin and, hence, clearly is no measure of spatial structure.

Summing over the absolute value of the structure factors, as in

(122) $$\bar{S}\;\equiv \;\sum_{q=0}^{\infty }\left|S\left(q\right)\right|\;,$$

also yields a quantity that fails to measure the total correlation of the system. For example, $\bar{S}$ fails to vanish for a paramagnet in the presence of an external field.

One also might be tempted to add together all the connected correlation functions. That is, combine the two-spin, three-spin, four-spin, etc., connected correlation functions. (For a discussion of how to extend the definition of connected correlation functions to more than two spins see, e.g., Ref. [60].) However, such a sum either diverges or is simply proportional to the free energy. Neither case leads to a measure of structure.

More specifically, one can show that the connected correlation functions are, up to factors of β, the coefficients in the Taylor expansion of logZ=βF in powers of local coupling constants $J_{i}^{\prime }$ attached to each site *i*, where F=U−TS is the Helmholtz free energy [60]. Thus, a sum of the connected correlation functions corresponds to setting $J_{i}^{\prime }=1$ in this expansion. Since this is outside of the series’ radius of convergence, we conclude that this sum will fail to converge unless the series terminates. If the series terminates, however, the resulting sum is a quantity proportional to the free energy *F*.

In summary, Figure 18 illustrates one of our main points: excess entropy measures the memory stored in spatial configurations and as such is sensitive to periodic structure at any period. As far as one can tell, statistical mechanics does not possess a quantity that has these properties. The structure factors are sensitive to periodic behavior, but only at particular wavenumbers. Furthermore, the structure factors do not measure memory, carrying units of [spins${}^{2}$], whereas E carries units of bits, which often can be usefully interpreted in terms of the pattern or structure in a configuration.

Since any periodic 1D spin configuration has $h_{\mu }=0$, a vanishing entropy rate together with a positive excess entropy is an unambiguous indication of periodic order. The two information processing “coordinates”, $h_{\mu }$ and E, provide a means for detecting periodicity. However, periodicity is just one type of structure. How can we discover and describe structure that is not periodic, i.e., structure that has a positive entropy density? Moreover, different structures can have the same period. For example, ⋯↑↓↓↓↑↓↓↓⋯ and ⋯↑↑↓↓↑↑↓↓⋯ both have E=2 bits corresponding to a period of 4.

More generally, “pattern” is not synonymous with memory as measured by E. Knowing the amount of memory stored in spatial configurations does not specify how the memory is organized. Put another (obvious) way, knowledge of E alone does not allow one to reproduce the original configuration ensemble. We shall consider these issues at length now.

Lastly, note that, like E, $C_{\mu }$ serves as a measure of low-entropy memory since since by Equation (106) $h_{\mu }=0$ implies $C_{\mu }=E$.

## 7. ϵ-Machines Reveal Structural Features in Entropic Processes

In the previous section we saw that E serves to detect periodic—i.e., $h_{\mu }=0$—structure. In this section we examine systems that have relatively large entropy density, yet still produce highly structured configurations. To describe these systems, E becomes inadequate and the full apparatus of computational mechanics becomes necessary.

### 7.1. Discovering and Describing Entropic Patterns

**Figure 19:**
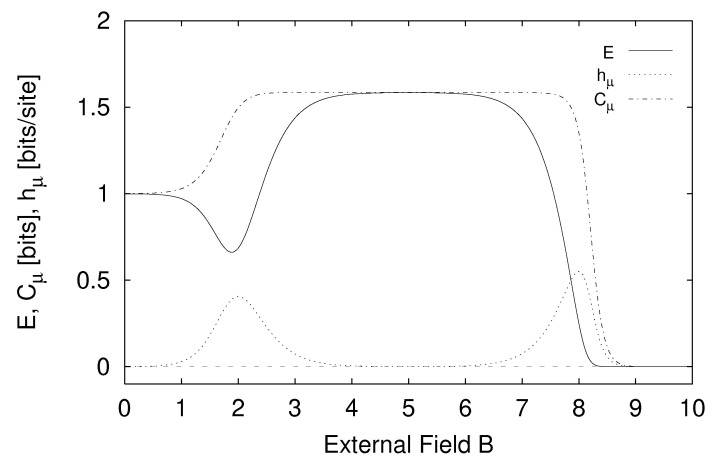
The statistical complexity $C_{\mu }$, excess entropy E, and entropy density $h_{\mu }$ versus external field *B* for a next-to-nearest neighbor 1D Ising system. The parameters are $J_{1}=-3.0$, $J_{2}=-1.0$, and T=0.2.

To illustrate this, we consider a spin-1/2 Ising model with next-nearest neighbor interactions, as analyzed in Section 5.3. We fix the coupling constants and the temperature at the following values: $J_{1}=-3.0$ and $J_{2}=-1.0$ at T=0.2. The temperature is small compared to the external parameters. Hence the system is close to its ground state and thermal excitations are small. As *B* increases, the ground state for the system changes. This can be seen by considering the excess entropy and the entropy density, which are plotted along with $C_{\mu }$ in Figure 19.

The previous section noted that a 1D spin system with $h_{\mu }=0$ is periodic with period $P=2^{E}$. For B<1/2, we see in Figure 19 that $h_{\mu }$ vanishes while E≈1 bit indicating a periodic structure of period 2. Similarly, E and $h_{\mu }$ vanish for B>8.5, indicating periodicity of period 1. For 4<B<6, E≈1.59 bits and $h_{\mu }\approx 0$, indicating that the system is in a configuration with period 3, since $\log_{2}3\approx 1.59$. Thus, as *B* is varied, the system makes transitions between three different spatially periodic ground states.

**Figure 20:**
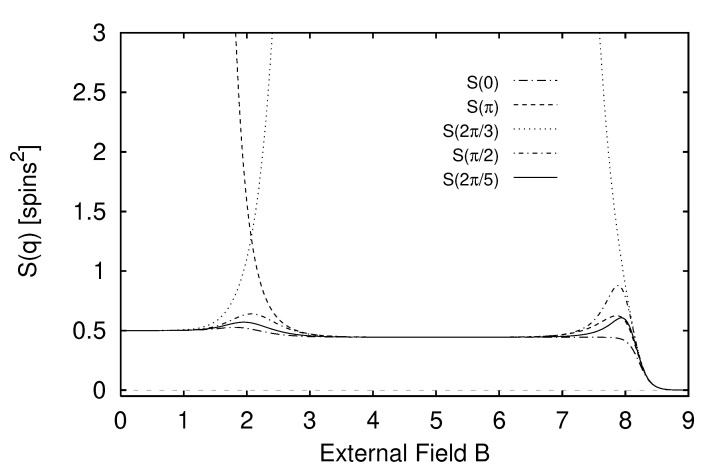
The structure factors S(0), S(π), S(2π/3), S(π/2), and S(2π/5) versus external field *B* for a next-to-nearest neighbor 1D Ising system with $J_{1}=-3.0$, $J_{2}=-1.0$, and T=0.2.

The transitions between different periodic regimes can also be seen in Figure 20 which shows plots of various structure factors as a function of the external field *B*. The period-2 structure factor S(π) diverges as *B* falls below 2. (Strictly speaking, the structure factor does not diverge. Since the temperature is nonzero, the structure factor remains finite. “Diverging” structure factors here have values around $10^{5}$.) Above B=8, all structure factors vanish, an indication that the system is in a ferromagnetic ground state. For *B* between 2 and 8, we see in Figure 20 that the period-3 structure factor S(2π/3) diverges. Note that our being able to detect these changes in the periodic structure of the system is due to judicious choices of *q* for the S(q)’s shown. This example again illustrates the utility of E as a wavelength-independent detector of periodicity.

We now turn our attention to the main question of this section: What is happening *during* the transitions between these periodic regimes? It is clear that we are witnessing a transition between different periodic behaviors. The structure factors or, for that matter, the excess entropy make this abundantly clear. However, *how* does this transition occur as *B* is varied? The S(q)’s do not help us answer this. Instead, we shall see that we have to examine the ϵ-machine.

For B=2 and B=8 we see in Figure 19 that the entropy density $h_{\mu }$ is large. Thus, the system is not spatially periodic in these regimes and cannot be well-described by structure factors. Presumably, the configurations are some mixture of the periodic ground states that dominate on either side of each transition. However, is this the case? Furthermore, if they are mixtures, how do two periodicities “mix”?

The structure factors do not provide much, if any, clue. Near B=8 in Figure 20 we see gentle peaks in S(π), S(π/2), and S(2π/5), the structure factors for patterns of periods 2, 4, and 5, respectively. Furthermore, near B=2, we see peaks in the S(q)’s for periods 1, 2, and 4. What sort of configuration could produce these structure factor amplitudes? To help us answer this question, we examine the ϵ-machines for the configurations at the transition points.

**Figure 21:**
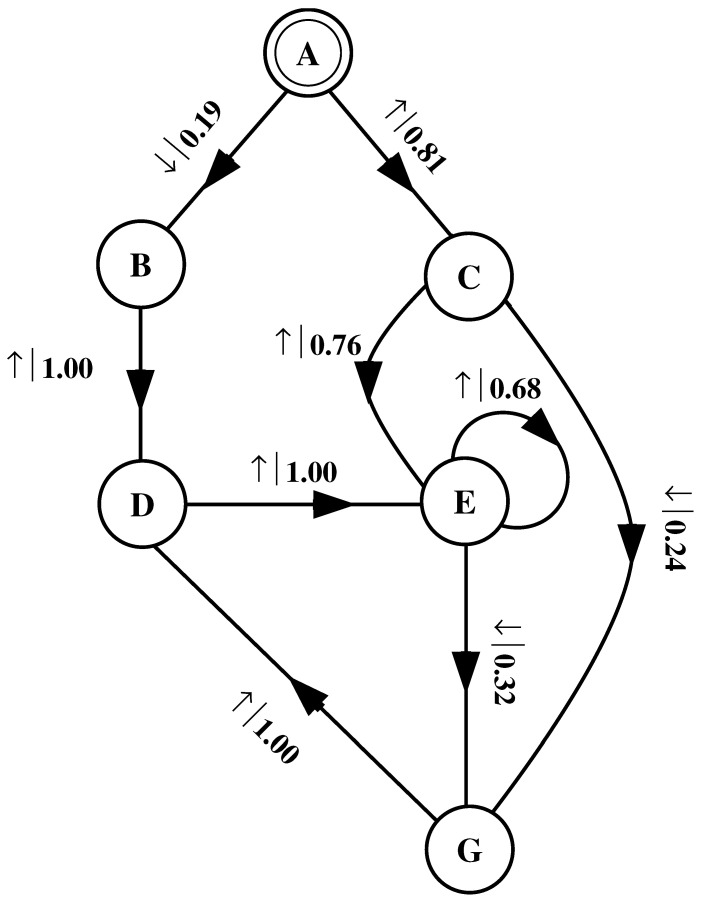
The ϵ-machine for the spin system shown in Figure 19 and Figure 20 with the external field fixed at B=8.0. The other parameters are $J_{1}=-3.0$, $J_{2}=-1.0$, and T=0.2.

Figure 21 shows the ϵ-machine for B=8.0. Transitions that occur with a probability of less than $10^{-6}$ are not shown. Note that this ϵ-machine has only 3 recurrent states, as opposed to the 4 recurrent causal states of the generic nnn ϵ-machine of Figure 17. State F has disappeared—it is reached with a probability of less than $10^{-7}$ and so is not included in Figure 21.

As expected, the ϵ-machine of Figure 21 demonstrates that the transitional structure is indeed a “mixture” of periodic behaviors of periods 1 and 3. States A, B, and C are transient states. The self-loop on state E gives the period-1 pattern …↑↑↑↑↑…. The E→G→D→E loop is the period-3 pattern …↓↑↑↓↑↑↓↑↑…. The entropy density for the configurations described by the ϵ-machine of Figure 21 is relatively high: $h_{\mu }\approx 0.551$ bits per spin. Nevertheless, the configurations have considerable structure—simply calling them random or “mostly random” is unnecessarily crude.

Note that Figure 21 is not the only way for a period-1 and a period-3 pattern to mix. For example, extra ↑’s could be inserted at both state E and state D. That is, there could be an additional self-loop on state D that occurs with a different probability than the self-loop on state E. Thus, the ϵ-machine provides more information than just showing that the configurations are a mixture of period-1 and period-3 patterns—the ϵ-machine tells us *how* the patterns combine.

Unlike the collection of statistics plotted in Figure 19 and Figure 20, the ϵ-machine provides a complete description of the configuration ensemble: The ϵ-machine is capable of statistically reproducing the entire original configuration, along with any other realizations consistent with the ensemble.

Recall that, as explained above, the ϵ-machine is a minimal description. First, the procedure of equivalence classing to determine the causal states ensures that the model has the fewest number of states while still accounting for all the causal structure of the system. Second, the model is chosen within the least powerful class that admits a finite description of the original process. Thus, in analogy with the group theoretic description of exact symmetries, the ϵ-machine may be viewed as the “irreducible representation” of the approximate symmetries. In this sense we conclude that the ϵ-machine *is* the pattern.

Note that to discover the pattern’s structure by building the ϵ-machine, no assumptions are made, aside from the translational invariance of the original configuration. That is, determining an ϵ-machine for a system is not a transform for detecting an a priori given set of patterns, as is the case with Fourier analysis and the structure factors, for example. Rather, ϵ-machines enable one to discover patterns and structures not assumed beforehand.

### 7.2. Detecting Entropic Patterns

The ϵ-machine directly reveals important and useful structural information about a configuration. As mentioned above, the machine of Figure 21 reveals how the period-1 and period-3 patterns mix to produce the configurations responsible for the complexities and structure factors observed at B=8.0 in Figure 19 and Figure 20. Furthermore, the ϵ-machine structure often can be easily translated to provide a compact description of the configuration in natural language, as already attempted in the preceding paragraphs. In the case just analyzed, configurations may be viewed as a background pattern of ↓↑↑’s with one or more extra ↑’s inserted before each ↓. Typical configurations at B=8 are:

(123) ↑↑↓↑↑↓↑↑↑↑↑↓↑↑↑↑↑↑↓↑↑↑↓↑↑↑↑↑↓↑↑↑↓↑↑↑↑↓

and

(124) ↑↑↓↑↑↓↑↑↑↓↑↑↓↑↑↑↑↓↑↑↑↓↑↑↑↓↑↑↑↑↑↓↑↑↓↑↑↑.

The probability that there are *M* extra ↑’s inserted is readily gleaned from the ϵ-machine in Figure 21: $\mathrm{Pr}\left(M\;\mathrm{extra}\;↑{}^{\prime }s\right)=0.32\times 0.68^{M}$, M=0,1,2,….

Equivalently, the ϵ-machine tells us how we can construct a different machine that produces similar configurations: a machine with a single state that generates the sequence ↓↑↑ with probability 0.32 and ↑ with probability 0.68. Note that this is *not* an ϵ-machine since each transition made by the machine does not produce one symbol.

**Figure 22:**
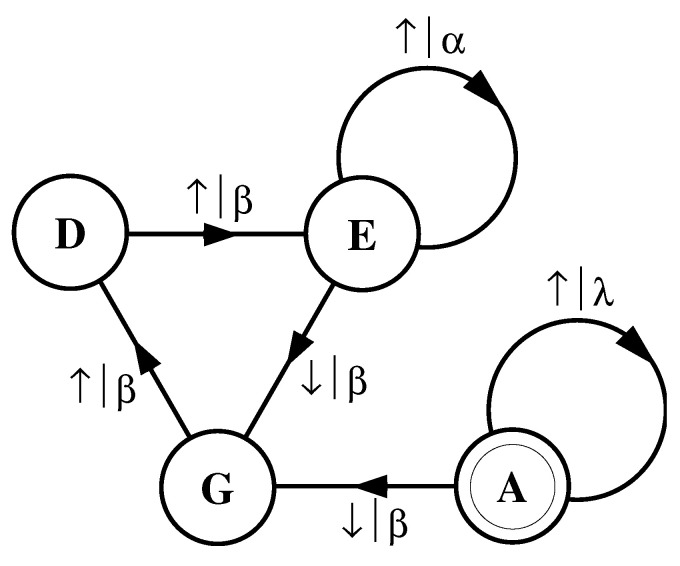
A transducer that detects the elemental spin subpatterns ↑↑↑↑… and ↓↑↑↓↑↑↓↑↑… and labels the lattice sites with the name—α or β, respectively—of the subpattern in which each site participates.

This alternative description lets us construct yet another machine, a *transducer*, that detects which sites are participating in the constituent subpatterns ${\alpha \;=\;↑}^{*}$ or $\beta ={\left(↓↑↑\right)}^{*}$, where $w^{*}$ denotes an arbitrary number of replications of *w*. This filtering machine, illustrated in Figure 22, defines a function from configurations of spins to sequences over the alphabet {α,β}. Transitions are selected deterministically according to which spin state is read in from the configuration. (See Refs. [45,126] for more discussion of building and using these types of transducers.)

Before any symbols are read in, the transducer begins in the start state, labeled A in Figure 22. If the first symbol read in is ↑ the transducer produces the null symbol λ and returns to state A. If the symbol is ↓, then the transducer is synchronized to the configuration: it “knows” what causal state the process is in and it outputs the symbol β, indicating that the observed ↓ is part of the ↓↑↑ pattern. The next two symbols read in will be ↑↑ and the transducer makes transitions from G to D and D to E. In this manner, the transducer maps the input string of ↑’s and ↓’s to a string of α’s and β’s. For example, the configuration of Equation (123) is mapped to:

(125) λλββββββαααβββαααααβββαβββααβ.

There are several features of the transducer that give it utility. First, the transducer can be viewed as giving “meaning” to individual spins [42]. Determining if a spin is part of the period-1 or period-3 pattern tells us what role that particular site is playing in the configuration. This is not a trivial observation since an isolated ↑ is a part of both of the two competing subpatterns.

Second, the transducer provides a way to recognize sequences; that is, to determine if a candidate sequence is statistically identical to the configuration from which the ϵ-machine was originally constructed. This recognition process consists of two components. First we determine if the candidate configuration is allowed. Then we check to see that the spin blocks within the candidate configuration occur with the correct probabilities.

If, as a transducer reads a configuration, a spin value is encountered for which there is no transition, then that configuration is rejected. We conclude that it is not a member of the configuration ensemble. For example, the sequence ↑↑↓↓ is rejected since there is no transition leaving state G when a ↓ is read.

To conclude that a configuration $\overset{↔}{s}$ is *statistically* consistent with the configuration from which the ϵ-machine was built, $\overset{↔}{s}$ must do more than correspond to a path through the transducer. It must also produce the correct percentage of α and β subpatterns. Figure 21 tells us that proper configurations are a type of a biased coin: with probability 0.68 a ↑ is generated and with probability 0.32 a ↓↑↑ is generated. If a configuration produced by the ϵ-machine of Figure 21 is used as input to the transducer of Figure 22, then the fraction $f_{\alpha }$ of α’s in the output produced is:

(126) $$f_{\alpha }=\frac{0.68}{0.68+3\times 0.32}\approx 0.42\;.$$

Thus, if a configuration is read in to the transducer and the fraction of α’s produced approaches 0.42, then we conclude that it is statistically identical to the original one.

For this case, the simple form of the ϵ-machine of Figure 21 lets us easily compute the finite-size scaling of the transducer output. Thus, thinking of the ϵ-machine as a biased coin, it immediately follows that the number of α’s expected in a length-*N* configuration is:

(127) $$\#\;\mathrm{of}\;\alpha {}^{\prime }s\approx 0.42\times N\pm \sqrt{0.42\times 0.58N}\;,$$

or,

(128) $$f_{\alpha }=0.42\pm 0.49N^{-1/2}\;.$$

Here we have ignored the number of spins needed to synchronize to the pattern. This simple calculation assumes, consistent with the law of large numbers, that the deviations from $f_{\alpha }$ are small. The correct way to estimate the fluctuations for ϵ-machines uses methods from large deviation theory, as done in Ref. [116].

Analytically calculating (as here) or empirically reconstructing ϵ-machines enables us to discover patterns. Analyzing the ϵ-machine reveals what the patterns are. Furthermore, the transducer—a simple modification of the ϵ-machine—tells us how we can detect these patterns. One can do more, such as calculate the expected error in the transducer’s output for finite-length input strings, as we have just outlined.

**Figure 23:**
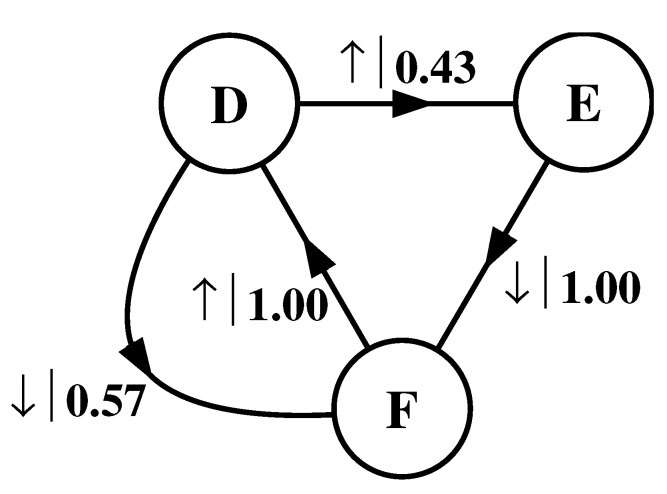
The recurrent portion of the ϵ-machine for the system shown in Figure 19 and Figure 20 with the external field fixed at B=2.0. The other parameters are $J_{1}=-3.0$, $J_{2}=-1.0$, and T=0.2.

A similar structural analysis of the configurations in the B=2 transition region follows from the ϵ-machine of Figure 23. For example, we again see a mixture of two periodic patterns. This time period-2 and period-3 subpatterns combine. Configurations consist of a “background” of ↑↓↑ with a period-2 component of ↓↑’s inserted between the two ↑’s of the background pattern. The probability that *M* period-2 blocks are inserted is $\mathrm{Pr}\left(M↑↓\mathrm{blocks}\right)=0.43\times 0.57^{M}\;,M=0,1,2,\ldots$.

In summary, the preceding subsections illustrated how ϵ-machines provide a complete, minimal description of the patterns or regularities contained in (entropic) spin configurations. Roughly speaking, they may be viewed as the irreducible representations of the statistical symmetries of the system. As such, an ϵ-machine provides a much more complete and informative description of a pattern than is available within information theory or statistical mechanics. In contrast, Figure 19 and Figure 20 do not strike us as being structurally very informative. It is clear from these plots that there is a transition between periodic behaviors, but the specifics of the structural changes are not at all obvious.

The dip in the excess entropy and the peak in the entropy density at the transition regions give a general indication of high-entropy, low-apparent-memory configurations. These structures are not periodic and, thus, are not compactly described by the structure factors that implicitly assume the system has strong periodic components. However, the configurations most certainly are not structureless. The ϵ-machine analyses showed that the constituent periodic patterns mix in very particular ways. This explicit analysis of patterns is not available within the existing frameworks of statistical mechanics or information theory.

Lastly, recall that our description of spin configurations began with a Hamiltonian with nearest and next-nearest neighbor interactions, which in turn led to a 4×4 transfer matrix. The Hamiltonian and the transfer matrix both determine all the information about the system in the sense that they can be used to calculate the probability, and thus the energy, of any configuration. However, neither the Hamiltonian nor the transfer matrix capture the intrinsic computational structure in the explicit way an ϵ-machine does nor do they provide a minimal description of the underlying patterns.

## 8. Phenomenological Comparison of Excess Entropy and Statistical Mechanical Quantities

At this point we have reached our two main conclusions. First, the entropy density $h_{\mu }$ and the excess entropy E together serve to detect periodic structure at any wavelength. If $h_{\mu }=0$, then 1D spin systems are exactly periodic with period $P=2^{E}$. Second, we have seen that the ϵ-machine *is* the underlying pattern in the sense that it is a minimal representation of all the (group and semigroup theoretic) regularities in a configuration ensemble. Excess entropy and ϵ-machines complement each other. E measures a system’s apparent spatial memory, while an ϵ-machine gives direct access to how a system is organized and how it processes information. The causal states, part of an ϵ-machine, reveal the hidden, effective states of a process.

Before we conclude, however, there are a few remaining issues that need addressing. Specifically, we need to explicitly compare the excess entropy with some of the statistical mechanical functions defined in Section 2 to see if there are additional statistical mechanical observables that could play the role that E does.

### 8.1. Excess Entropy versus Correlation Length

**Figure 24:**
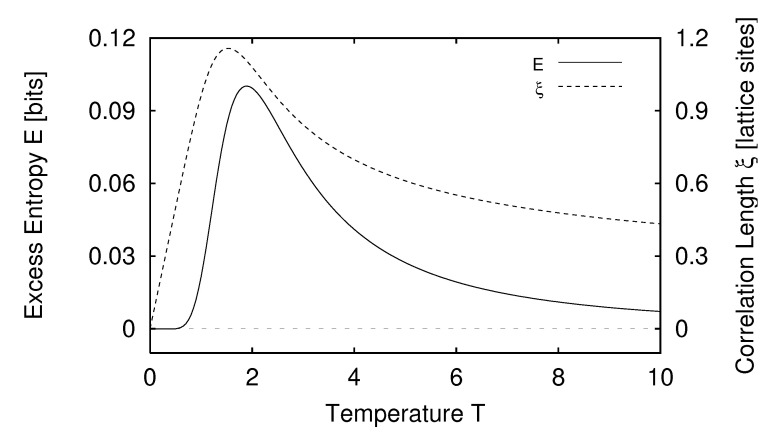
E and ξ versus *T* for nn ferromagnetic coupling with J=1.0 and B=0.5.

We begin by comparing excess entropy with the correlation length ξ, defined by Equation (15). Qualitatively, their behavior is similar, as can be seen in Figure 24 where they are plotted as a function of temperature for a ferromagnetic system in an external field. These two functions have different units; E is measured in bits, while ξ is a length measured as a number of lattice sites. Thus, their relative magnitudes cannot be meaningfully compared.

However, we can compare their qualitative behavior as the temperature is varied. Looking at Figure 24, we see that both quantities have a single maximum as a function of temperature. However, their maxima occur at different temperatures: ξ is maximized at T≈1.55, while E reaches a maximum at T≈1.90. This indicates that they are not related to each other by a simple multiplicative constant. Moreover, ξ is linear for small *T* while E vanishes exponentially.

A more important difference, though, is that E and ξ have very different physical interpretations. On the one hand, the correlation length ξ measures the *rate* at which correlations between spins decay as a function of increasing distance. The decay rate provides little or no information about how much total correlation or memory is present. The excess entropy, on the other hand, measures the mutual information between two semi-infinite halves of the configuration and thus provides a measure of the total spatial memory of the system.

### 8.2. Excess Entropy versus Specific Heat

**Figure 25:**
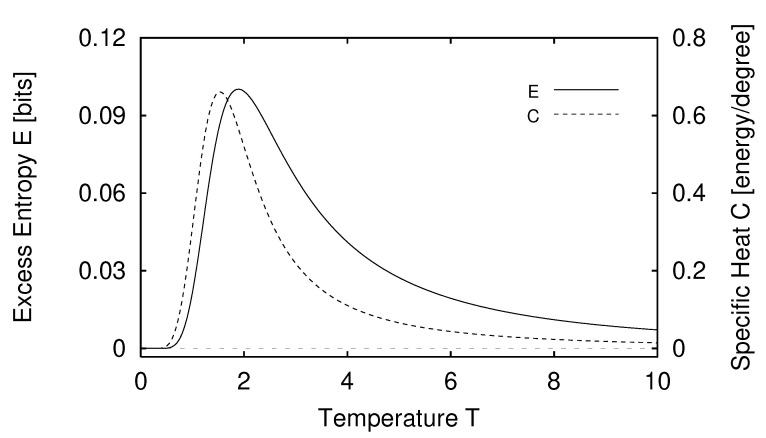
E and specific heat *C* versus *T* for a nn ferromagnet with J=1.0 and B=0.5.

Figure 25 plots the specific heat *C* and the excess entropy E as a function of temperature. As in the comparison of E and ξ, they carry different units and so their numerical values cannot be compared. Qualitatively, their behavior is more similar than found in the comparison above. However, they are maximized at different temperatures; *C* reaches a maximum at T≈1.55 while E, as above, attains its maximum value at T≈1.90.

Despite the similarities, these two quantities measure very different properties of the system. As mentioned in discussing Equation (25), the specific heat measures the system’s energy fluctuations. While these fluctuations may be evidence of correlations between different degrees of freedom, leading to a large E, this most certainly is not always the case. For example, a paramagnet has a nonzero specific heat that shows a single maximum just as *C* does in Figure 25. Yet a paramagnet, by definition, has no correlations between spins. Accordingly, the excess entropy of a paramagnet vanishes for all values of the temperature and the external field. Since the specific heat does not vanish for such a system, it is clear that *C* cannot be viewed as providing any general indication of spatial structure.

### 8.3. Excess Entropy versus Particular Structure Factors

**Figure 26:**
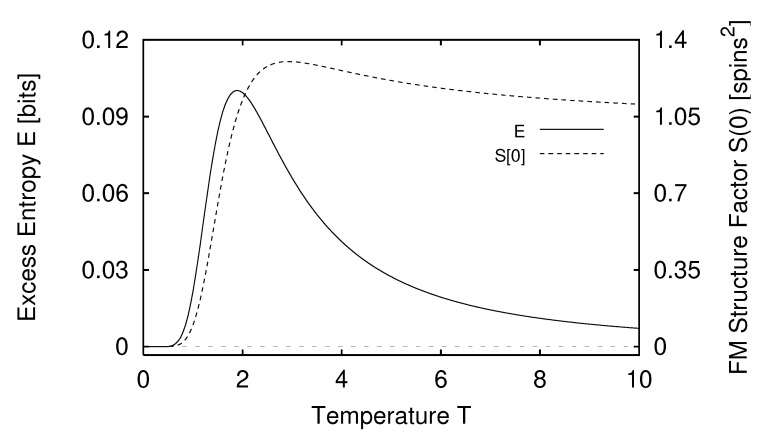
E and S(0) versus *T* for a nn ferromagnet with J=1.0 and B=0.5.

Figure 26 plots E and S(0) versus temperature. The system is ferromagnetic with J=1.0 and B=0.5. Thus, we chose to plot the structure factor for q=0 since a priori we expect ferromagnetic behavior—i.e., configurations with period 1. The behavior of E in this case has been discussed above.

In the low temperature limit S(0) vanishes. Since all the spins align with the magnetic field as T→0, ${\langle s\rangle }^{2}$ and $\langle s_{0}s_{r}\rangle$ approach 1 for all *r*. Hence, $\Gamma \left(r\right)=\langle s_{0}s_{r}\rangle -{\langle s\rangle }^{2}=0$ and so S(0) vanishes.

In contrast, the high-temperature behavior of S(0) is a little surprising—based on the above argument one would expect S(0) to go to zero as *T* goes to infinity and as the correlations vanish. However, recall that S(0) contains a “self-correlation” term, $\Gamma \left(0\right)=\langle s_{0}s_{0}\rangle -{\langle s\rangle }^{2}$. At high temperatures, the spins are randomly oriented so ${\langle s\rangle }^{2}=0$. However, $\langle s_{0}s_{0}\rangle =1$ for all temperatures since $s_{0}\in \left\{+1,-1\right\}$. Thus, Γ(0)→1 as T→∞, so S(0)→1 as T→∞.

In between these temperature extremes, there is a region where the correlation between spins is largest. Here, the system is neither random, as it is at high temperatures, nor is it trivially ordered, as it is at low temperatures. Not surprisingly, both S(0) and E reach a single maximum in the intermediate regime.

However, note that E and S(0) attain their maxima at different temperature values. The structure factor S(0) is maximized at T≈2.90, and E is maximized at T≈1.90. As discussed in Section 6, a given structure factor is designed to return a large signal if there are correlations present at that wavenumber. Its numerical value does not have a direct interpretation.

The shape of the curve in Figure 26 is unchanged if either of the two modified structure factors defined in Equations (22) and (23) are substituted for S(0). Furthermore, none of these structure factors are maximized at the same temperature that maximizes E.

### 8.4. Excess Entropy versus Γ(1)

**Figure 27:**
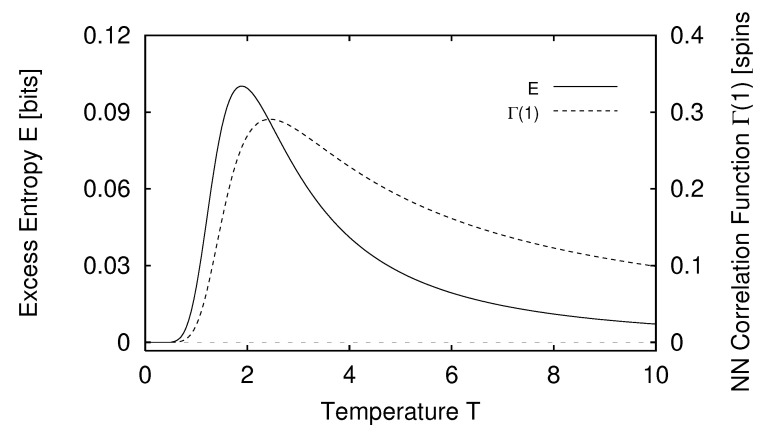
E and Γ(1) versus *T* for a nn ferromagnet with J=1.0 and B=0.5.

As our last phenomenological comparison, Figure 27 plots the nearest-neighbor correlation function Γ(1) and the excess entropy E as a function of the temperature *T*. Like the structure factor, Γ(1) carries units of $\left[{\mathrm{spins}}^{2}\right]$, not bits. As in the preceding examples, the two functions are maximized at different temperatures: Γ(1) is maximized at T≈2.50.

That Γ(1) and E reach a maximum at different temperatures is especially noteworthy since we are considering a system with only nearest-neighbor interactions. One might reasonably expect that for a system with such local, pairwise interactions, the nearest-neighbor two-spin correlation function would be sufficient to capture the system’s global correlations. Figure 27 shows that this is not the case. Even for a system with nn interactions, the nearest-neighbor correlation function does not measure apparent spatial memory as the excess entropy does.

### 8.5. Phenomenological Observations

Statistical mechanics possesses several functions that are similar to the excess entropy, but none can be interpreted as measures of spatial memory as E can be. We saw that the correlation length, specific heat, and the structure factors exhibit behavior qualitatively similar to the excess entropy for the particular class of systems studied here. However, none of these statistical mechanical quantities returns a numerical value that quantifies memory. The excess entropy, being defined as a mutual information, carries units of bits, appropriate for this type of structural feature.

Moreover, this section demonstrated that each of these quantities reaches a maximum at different parameter values. This means that the statistical mechanical functions cannot be used to determine the parameter setting at which a given system’s spatial memory is the largest. Simply put, to measure (apparent) spatial memory, one must use E.

## 9. Conclusions

In summary, we reviewed three complementary approaches to correlational structure. Section 2 briefly recounted statistical mechanical measures of structure: the correlation length ξ, the two-spin correlation function Γ(r), and the structure factors S(q). Section 3 discussed an information-theoretic approach to memory and structure, first reviewing different forms of the Shannon entropy *H* and then focusing on the excess entropy E. Last, Section 4 reviewed computational mechanics, a computation-theoretic approach to memory and structure, and introduced the ϵ-machine, a minimal representation of the deterministic and statistical regularities of a system. Section 5 then showed how ϵ-machines and the excess entropy E can be determined for one-dimensional, finite-range spin systems.

The next three sections developed a direct comparison of statistical-mechanical, information-theoretic, and computational-mechanical approaches to structural complexity. There were three main conclusions that emerged as a result of these comparisons.

First, Section 6 showed that the excess entropy E serves as a wavelength-independent measure of periodic structure. In particular, if a spin system is periodic with period P then $h_{\mu }=0$ and $E=C_{\mu }=\log_{2}P$.

Second, Section 7 showed that to fully capture the structure in highly entropic systems, one must examine ϵ-machines. This is reflected in the explicit relationship we derived in Equation (106) between entropy density, excess entropy, and statistical complexity: $C_{\mu }=E+Rh_{\mu }$. An ϵ-machine reveals how the memory is organized and gives all of the system’s (measure) semigroup theoretic properties.

Finally, Section 8 explicitly compared the excess entropy to the specific heat, correlation length, nearest-neighbor correlation function, and the ferromagnetic structure factor. We saw that these statistical mechanical functions behave similarly, but not identically to E. More importantly, none of these functions has a numerical value that can be directly interpreted as memory, as E can be. In short, then, our comparison of different approaches to structure showed that information theory and computational mechanics capture important properties of a system that statistical mechanics misses.

Several ancillary observations, based on the foregoing results, are now in order. First, we have seen that for 1D spin systems the number of causal states and their connectivity typically does not change as spin system parameters are varied. What does change, however, are the probabilities of the causal states and their transitions. In contrast, for deterministic dynamical systems it is typically the number of causal states that change as the system parameters are varied [25,43]. Thus, it is our belief that “topological” measures of structure or complexity such as those of Refs. [127,128]—i.e., those that account for configurations only in terms of whether they are allowed or disallowed, and so ignore their probabilities—will not adequately capture important structural changes in statistical mechanical systems.

Second, approaches to structural complexity, such as those of Refs. [30,34,129], that are based on the Kolmogorov–Chaitin (KC) complexity, strike us as being of little use for addressing the questions of pattern and organization posed here. Our concerns about these KC complexity-based approaches are three-fold.

First, by using a universal Turing machine (UTM), the most powerful discrete computational model, one loses the ability to distinguish between systems that can be described by different computational models less powerful than a UTM [16,25].

Second, and perhaps more importantly, the KC complexity is uncomputable in that there exists no general algorithm for its computation. Thus, approaches focusing on KC complexity, including logical depth [30] and sophistication [111], tend to be nonconstructive. In contrast, in the mathematical domain there are broad classes of processes for which the excess entropy and ϵ-machines can be determined. In the empirical domain, moreover, there exist algorithms for estimating the excess entropy and determining an ϵ-machine. The computational complexity of these algorithms is determined by the class of processes analyzed. Indeed, for the 1D spin systems studied here, we gave closed-form expressions for various complexity measures of interest.

Third, KC complexity-based approaches inherit a fundamental relativity—a relativity that is built into how regularity and structure are accounted for and that derives from the UTM’s lack of uniqueness and minimality. Computational mechanics takes a completely different approach and makes a specific commitment to causal states and ϵ-machines as a fundamental representation for the intrinsic computation embedded in a process. It also associates this, via the algebraic structure of ϵ-machines, with a system’s internal organization and the patterns the system produces.

Thus, given the problems arising from KC complexity being based on UTMs, it seems to us that these approaches to structure and pattern will continue to find few empirical applications. Significant supplemental assumptions would have to be introduced to make these approaches viable. In contrast, due to its specificity of representation, computational mechanics is testable and its hypotheses—e.g., linking pattern, organization, and computation—are refutable.

We conclude by discussing some open questions and possible areas of application. It remains an interesting open question as to how E characterizes quasiperiodic or more general $h_{\mu }=0$ aperiodic configurations. Unfortunately, the simple spin systems analyzed here are not rich enough to address this question. Another important set of issues concerns extending the information theoretic and computational mechanics approaches to more than one spatial dimension. Just as statistical mechanics in higher spatial dimensions is markedly different than in one dimension, both information theory and computational mechanics will have to be significantly extended. For example, even the scanning of site values in two-dimensional configurations becomes ambiguous [24,28,130,131] and so is an important problem in its own right, unlike in 1D. There has been some preliminary work on complexity in two spatial dimensions [46,78,127], but much remains to be done. For example, some work done on two-dimensional systems [40,132,133] calculates quantities that are essentially one-dimensional in character and so fail to adequately capture the nature of correlations and organization in two dimensions. In our view, a careful, genuinely two-dimensional treatment of a two-dimensional system is still lacking. A review of the state of affairs and some preliminary results can be found in Ref. [1].

Another important set of open questions concerns the development of better techniques for estimating the excess entropy and reconstructing ϵ-machines in experimental settings. At present, there is no complete theory of statistical error estimation for inferring ϵ-machines from finite data. Another somewhat related, and perhaps more important issue, concerns developing direct methods for experimentally estimating (say) excess entropy in a wider range of settings than those in which digitized data streams are available. For example, the structure factors S(q) can be estimated from neutron scattering experiments in a natural way. Can E also be measured in an analogously direct fashion? If so, the discovery and characterization of novel materials would be greatly facilitated.

Finally, there are a number of issues that parallel our comparisons with elementary statistical mechanics, but that relate to phase transitions and critical phenomena. Are the intrinsic computational properties reflected in the excess entropy or in infinite ϵ-machines the same within universality classes? If they are universal, do they scale with different exponents and so capture different aspects of (say) a critical state than presently appreciated? Or do computational mechanical and information theoretic quantities fall short of current notions of universality? Since they reflect rather more detailed features of the underlying distributions than typical universal quantities, they may not be universal. However, if they fail to be universal, there may be some unsettling conclusions about using concepts and methods from the theory of phase transitions and critical phenomena to study information processing in nature. In either case, we believe that answering these questions will be an essential step toward understanding how nature organizes and how its emergent structures take on functionality.

## Figures and Tables

**Table 1:** The entropy density $h_{\mu }$, the excess entropy E, and the statistical complexity $C_{\mu }$ for the four example processes of Section 4.1.

Process	$h_{\mu }$	*E*	$C_{\mu }$
**Fair Coin**	1	0	0
**Period 1**	0	0	0
**Period 2**	0	1	1
**Noisy Period 2**	1/2	1	1

**Figure A1:**
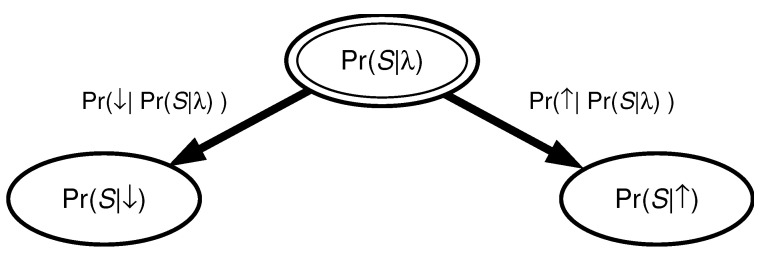
First steps in performing transient state construction for the case of a spin-1/2 system where $s_{i}\in \left\{↑,↓\right\}$. The double oval indicates the start node $n_{0}$.

**Figure A2:**
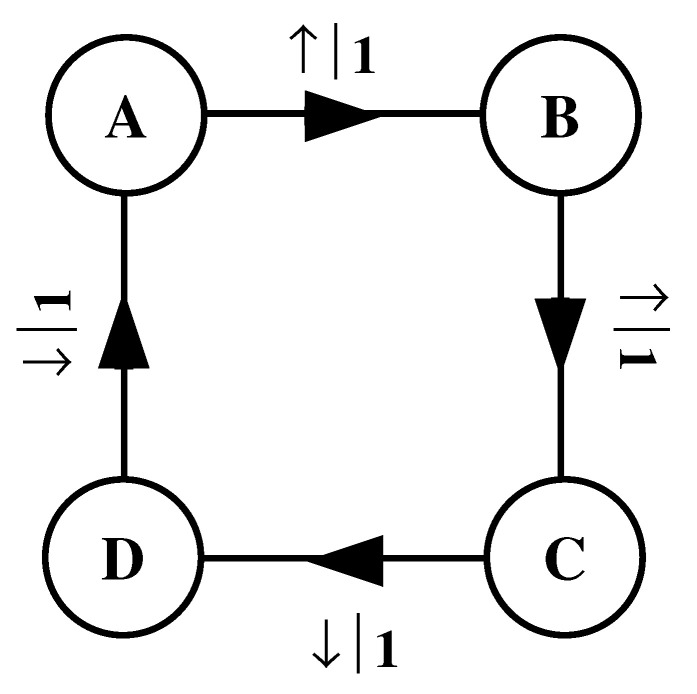
Recurrent portion of the period-4 ϵ-machine.

**Figure A3:**
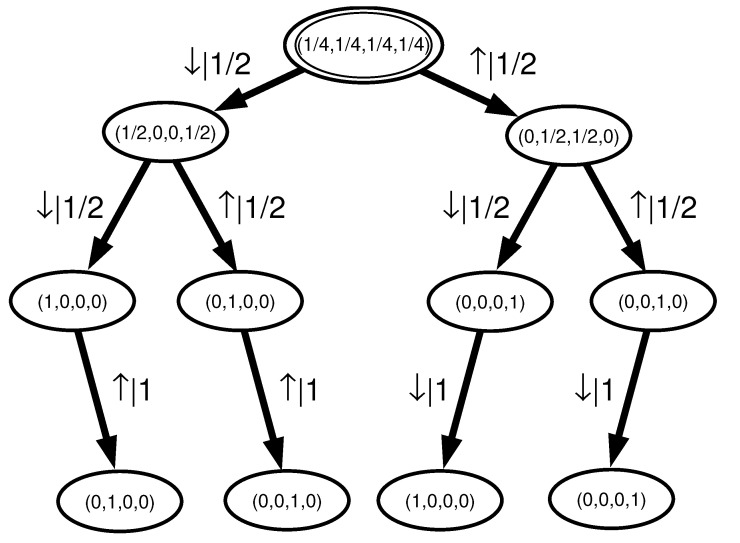
The tree T part of the way through transient state construction for the period-4 process. To construct this tree spin blocks up to length 3 were examined.

**Figure A4:**
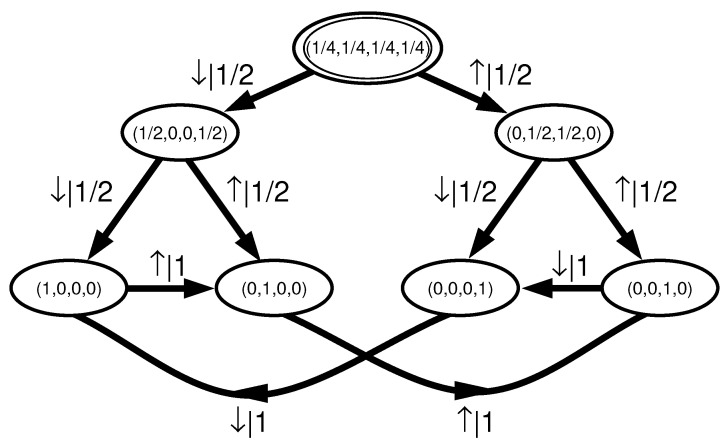
The end result of transient state construction for the period-4 process after duplicate leaves are removed.

## Appendix A. On the Equivalence Relation∼that Induces Causal States

Consider the set $\overset{\leftarrow }{X}$ of all left-half configurations, of any length:

(A1) $$\overset{\leftarrow }{X}=\{X_{:0}^{L}=s_{L-1}\cdots s_{-1}:\;s_{i}\in A,\;L=0,1,\ldots \}\;.$$

Recall that $X_{:0}^{0}=\lambda$, the empty string. It was claimed in Equation (53) that

(A2) $${\overset{\leftarrow }{s_{i}}}^{K}∼{\overset{\leftarrow }{s_{j}}}^{L}\;\Leftrightarrow \;\mathrm{Pr}\left(X_{0:}|{\overset{\leftarrow }{s_{i}}}^{K}\right)=\mathrm{Pr}\left(X_{0:}|{\overset{\leftarrow }{s_{j}}}^{L}\right)\;,$$

for all semi-infinite $X_{0:}=s_{0}s_{1}s_{2}\cdots$, where K,L=0,1,2,…, defined an equivalence relation ∼ over $\overset{\leftarrow }{X}$. Here we show that this is indeed the case by reviewing the basic properties of relations, equivalence classes, and partitions. (The proof details are straightforward and are not included. See Ref. [134].) We will drop the length variables *K* and *L* and denote by $X_{:0},X_{:0}^{\prime },{\overset{\leftarrow }{x}}^{\prime \prime }\in \;\overset{\leftarrow }{X}$ members of any length in the set of Equation (A1).

First, ∼ is a relation on $\overset{\leftarrow }{X}$ since we can represent it as a subset of the Cartesian product

(A3) $$\overset{\leftarrow }{X}\times \overset{\leftarrow }{X}=\left\{\left(X_{:0},X_{:0}^{\prime }\right):X_{:0},X_{:0}^{\prime }\in \overset{\leftarrow }{X}\right\}\;.$$

Second, the relation ∼ is an equivalence relation on $\overset{\leftarrow }{X}$ since it is

1. reflexive: $X_{:0}∼X_{:0},\;\forall \;X_{:0}\in \overset{\leftarrow }{X}$;
2. symmetric: $X_{:0}∼X_{:0}^{\prime }\Rightarrow X_{:0}^{\prime }∼X_{:0}$; and
3. transitive: $X_{:0}∼X_{:0}^{\prime }\;\mathrm{and}\;X_{:0}^{\prime }∼{\overset{\leftarrow }{x}}^{\prime \prime }\Rightarrow X_{:0}∼{\overset{\leftarrow }{x}}^{\prime \prime }$.

Third, if $X_{:0}\in \overset{\leftarrow }{X}$, the equivalence class of $X_{:0}$ is

(A4) $$\left[X_{:0}\right]=\left\{X_{:0}^{\prime }\in \overset{\leftarrow }{X}:X_{:0}^{\prime }∼X_{:0}\right\}\;.$$

The set of all equivalence classes in $\overset{\leftarrow }{X}$ is denoted $\overset{\leftarrow }{X}/∼$ and is called the *factor set* of $\overset{\leftarrow }{X}$ with respect to ∼. In Section 4.2 we called the individual equivalence classes causal states $S_{\alpha }$ and denoted the set of causal states $S=\{S_{\alpha }:\alpha =0,1,\ldots ,k-1\}$. That is, $S=\overset{\leftarrow }{X}/∼$. (We noted in the main text that k=|S| may or may not be finite.)

Finally, we list several basic properties of the causal state equivalence classes.

1. ${⋃}_{X_{:0}\in \overset{\leftarrow }{X}}\left[X_{:0}\right]=\overset{\leftarrow }{X}\;$.
2. ${⋃}_{\alpha =0}^{k-1}S_{\alpha }=\overset{\leftarrow }{X}\;$.
3. $\left[X_{:0}\right]=\left[X_{:0}^{\prime }\right]\Leftrightarrow \;X_{:0}∼X_{:0}^{\prime }\;$.
4. If $X_{:0},X_{:0}^{\prime }\in \overset{\leftarrow }{X}$, either
  (a) $\left[X_{:0}\right]⋂\;\left[X_{:0}^{\prime }\right]=\emptyset$ or
  (b) $\left[X_{:0}\right]=\left[X_{:0}^{\prime }\right]\;$.
5. The causal states S are a partition of $\overset{\leftarrow }{X}$. That is,
  (a) $S_{\alpha }\neq \emptyset$ for each α,
  (b) ${⋃}_{\alpha =0}^{k-1}S_{\alpha }=\overset{\leftarrow }{X}$, and
  (c) $S_{\alpha }\cap S_{\beta }=\emptyset$ for all α≠β.

We denote the start state with $S_{0}$. The start state is the causal state associated with $X_{:0}=\lambda$. That is, $S_{0}=\left[\lambda \right]$.

Each causal state equivalence class $S_{\alpha }$ thus has several structures attached:

1. The index α—the state’s “name”.
2. The set of left-half configurations, of various lengths, comprising the equivalence class: $\left[X_{:0}\right]=\left\{X_{:0}\in S_{\alpha }\right\}$.
3. A conditional distribution over right-half configurations: $\mathrm{Pr}\left(\vec{X}|X_{:0}\right),X_{:0}\in S_{\alpha }$. We denote this distribution more concisely by $\mathrm{Pr}(\vec{X}|S_{\alpha })$.

As noted in the text, the definitions and properties of the causal states obtained by scanning in the opposite direction, i.e., the causal states $\vec{X}/∼$, follow similarly. For general processes, $\overset{\leftarrow }{X}/∼\;\neq \;\vec{X}/∼$.

For completeness, we note that this construction of causal states is analogous to Nerode equivalence, used to determine the minimal number of states for a finite-state machine representation of a regular language [94,135]. It is also somewhat similar to the states estimated in Rissanen’s “context” algorithm [136]. Despite these similarities, there are important differences. With Nerode equivalence infinite strings and probability measures over them are not considered. For a random source—for which there is a single causal state—the context algorithm estimates a number of states that diverges (logarithmically) with the length of the data stream. In addition, there is a class of finite-automata learning algorithms that are analogous to ϵ-machine reconstruction; cf. that in Ref. [135]. Finally, the notion of causal state should be compared to the elements of minimal semigroups discussed in Ref. [112].

## Appendix B. Transient Structure from the Recurrent ϵ-Machine

This appendix shows how transient states can be constructed from the recurrent portion of an ϵ-machine. The latter, denoted $M^{\left(R\right)}$, consists of the recurrent causal states $S^{\left(R\right)}$ and their transitions $T_{\alpha \beta }^{\left(s\right)}$, where the indices α and β run over only the recurrent causal states. That the transient states can be constructed in this manner is a direct consequence of the equivalence relation ∼, defined in Equation (53), that induces the causal states.

The basic idea of the construction procedure, detailed below, is as follows. First, we assume the generating process has been operating sufficiently long so that it is in equilibrium in the sense that it is being controlled by its recurrent causal states. We also assume we have a model of the process, namely $M^{\left(R\right)}$, in hand. Then we begin making measurements—reading in spin values from a configuration—$s_{0}s_{1}s_{2}\ldots$. With each measurement, we ask: In which recurrent causal state is the process? Initially, while making measurements and, of course, even before making the first, we are uncertain about which recurrent causal state the process is in. Thus, we describe our state of knowledge by a distribution over $S^{\left(R\right)}$, denoted $\mathrm{Pr}(S^{\left(R\right)}|s_{0}s_{1}s_{2}\cdots s_{L-1})$. As we observe successive spins, this distribution changes. The structure of the machine $M^{\left(R\right)}$ determines the change when we observe an individual spin. Presumably, with a sufficient number of spin measurements we become *synchronized* with the process: That is, we know with certainty in which recurrent state the process is. This procedure of tracking how our state of knowledge $\mathrm{Pr}(S^{\left(R\right)}|s_{0}s_{1}s_{2}\cdots s_{L-1})$ refines and focuses on smaller and smaller subsets of $S^{\left(R\right)}$ determines the transient causal states. From here on we will drop the superscript (R) on S, when no ambiguity arises.

Since we assume the process is in equilibrium, we take the initial probability distribution—that associated with having made no measurements—to be the asymptotic distribution over the recurrent causal states, which is given by Equation (63). This initial distribution is denoted Pr(S|λ) to indicate that it is the distribution before any spins have been observed. (Recall that λ denotes the empty string.) In this way, the start state represents the knowledge that the process is somewhere in its set of recurrent states. After observing spin $s_{0}$ our state of knowledge about the recurrent causal state of the process has improved and is now described by the distribution $\mathrm{Pr}(S|s_{0})$. Upon observing the next spin $s_{1}$, our state of knowledge becomes $\mathrm{Pr}(S|s_{0}s_{1})$.

We may associate the recurrent causal state distributions with the causal states themselves. For example, a distribution that specifies a recurrent causal state with certainty—i.e., probability 1 of being in that state—can be taken to be that recurrent state. The transient states, in contrast, are those distributions in which the recurrent causal state is not known with certainty.

The procedure through which the transient states are deduced proceeds by constructing a tree T={N,L} consisting of a set of nodes N and a set of links L connecting the nodes. A node n∈N in the tree corresponds to a recurrent causal state distribution $\mathrm{Pr}(S|s_{0}s_{1}\cdots s_{L-1})$. A link $\ell_{s}\in L$ corresponds to a transition between successive causal state distributions that occurs upon observing a particular spin value *s*. We call a tree node for which all the outgoing links have yet to be determined a *leaf*. We denote the set of leaves by $\hat{N}$.

The tree is constructed recursively via the following steps:

1. **Initialize:** Given a recurrent ϵ-machine $M^{\left(R\right)}=\left\{S^{\left(R\right)},T_{\alpha \beta }^{\left(s\right)}\right\}$ determine the asymptotic probability of the recurrent causal states via Equation (63). This distribution is the starting node for the tree $n_{0}=\mathrm{Pr}\left(S|\lambda \right)$ and is indicated in Figure A1 by the node with the double oval. At this stage $n_{0}$ is a leaf, since we have not yet determined all the links (transitions) that leave it. Thus, N=∅ and $\hat{N}=\left\{n_{0}\right\}$.
2. **Build Transient Tree:** While $\hat{N}$ is nonempty:
  (a) **Determine Links:** For each leaf $n=\mathrm{Pr}\left(S|s^{L}\right)\in \hat{N}$ draw a link $\ell_{s}\in L$ (an outgoing transition) for each spin value *s*. Label the link with the transition probability Pr(s|n) that starting in node *n* spin value *s* is seen:
    (A5) $$\mathrm{Pr}\left(s|n\right)=\sum_{S^{\prime }\in S^{\left(R\right)}}\mathrm{Pr}\left(S^{\prime }\right)\mathrm{Pr}\left(s|S^{\prime }\right)\;.$$
    If the transition has zero probability, ignore $\ell_{s}$.
  (b) **Form Node Distributions:** For each link $\ell_{s}$ determine the probability distribution $\mathrm{Pr}(S|s^{L}s)$ to which it leads using:
    (A6) $$\begin{array}{c} \mathrm{Pr}(S|s^{L}s)=\frac{\sum_{S^{\prime }\in S^{\left(R\right)}}\mathrm{Pr}\left(S^{\prime }|s^{L}\right)\mathrm{Pr}\left(S|s,S^{\prime }\right)}{\sum_{S^{\prime },S\in S^{\left(R\right)}}\mathrm{Pr}\left(S^{\prime }|s^{L}\right)\mathrm{Pr}\left(S|s,S^{\prime }\right)}\;. \end{array}$$
    Note that the term in the denominator is simply a normalization. The quantity $\mathrm{Pr}(S|s^{L}s)$ gives the updated distribution over recurrent causal states after having observed the particular spin sequence $s^{L}s$. Recall that, since the ϵ-machine is deterministic, $\mathrm{Pr}(S|s,S^{\prime })=1$ if the transition is allowed and 0 otherwise.
  (c) **Merge Duplicate Nodes:** Now consider, in turn, the probability distributions just formed: $n=\mathrm{Pr}(S|s^{L}s)$. Is *n* identical to another node distribution $n^{\prime }\in N$?
    - If yes, then connect $\ell_{s}$ to node $n^{\prime }$.
    - If no, add *n* to the set $\hat{N}$ of tree leaves.
3. **Minimize**: The resulting machine has a recurrent part that is identical to $M^{\left(R\right)}$, but it may not be minimal. Merge nodes pairwise under the equivalence relation ∼ of Equation (53). The result is the complete ϵ-machine, with all transient and recurrent states.

We illustrate the above procedure by considering a period-4 process. The recurrent portion of its ϵ-machine $M^{\left(R\right)}$ is shown in Figure A2. The result of the first several steps of the transient state construction procedure is illustrated in Figure A3. The asymptotic probability of each recurrent causal state is 1/4. Thus, as per step 1 above, the start node is labeled Pr(S|λ)=(1/4,1/4,1/4,1/4); it is shown as the double oval of Figure A3.

From the start node, two transitions are possible—one for s=↑ and one for s=↓. In the figure they are labeled with the transition probabilities as given by Equation (A5). For example, from $n_{0}$ the probability of seeing s=↑ is given by:

(A7) $$\begin{array}{c} \begin{array}{ccc} \mathrm{Pr}(↑|n_{0}) & = & \mathrm{Pr}(A)\mathrm{Pr}(↑|A)+\mathrm{Pr}(B)\mathrm{Pr}(↑|B)+\mathrm{Pr}(C)\mathrm{Pr}(↑|C)+\mathrm{Pr}(D)\mathrm{Pr}(↑|D)\\ & = & (1/4)(1)+(1/4)(1)+(1/4)(0)+(1/4)(0)=1/2\;. \end{array} \end{array}$$

The leaves (causal state distributions) to which the links (transitions) lead are determined by Equation (A6). For example, consider the ↓ transition from $n_{0}$. The normalization factor, the denominator in Equation (A6), is 1/2 and the probability of being in causal state A is given by:

(A8) $$\begin{array}{c} \begin{array}{c} \mathrm{Pr}\left(S=A|↓\right)=2\sum_{S^{\prime }\in S^{\left(R\right)}}\mathrm{Pr}\left(S^{\prime }\right)\mathrm{Pr}(A|↓,S^{\prime })\\ =2\left[\mathrm{Pr}(A)\mathrm{Pr}(A|↓,A)+\mathrm{Pr}(B)\mathrm{Pr}(A|↓,B)\right.+\mathrm{Pr}\left(C\right)\mathrm{Pr}\left(A|↓,C\right)+\left.\mathrm{Pr}(D)\mathrm{Pr}(A|↓,D)\right]\\ =2\left[(1/4)(0)+(1/4)(0)+\right.\left.(1/4)(0)+(1/4)(1)\right]\;=\;1/2\;. \end{array} \end{array}$$

Similarly, one finds that Pr(D|↓)=1/2 and Pr(B|↓)=Pr(C|↓)=0. As a result, this node is associated with the distribution Pr(S|↓)=(1/2,0,0,1/2).

The process of adding links and nodes to the tree T is shown repeated up to length-3 spin blocks in Figure A3. At the last level of the tree, transitions that occur with probability 0 are not drawn. At this point, notice that the leaves at the bottom level have already appeared as nodes above in the tree. For example, the lower left leaf is identical to the node that is second from the left, one level above. Thus, as per step 2(c)i, the link pointing to the leaf node is directed to the pre-existing node. This reconnection step is repeated until there are no leaves left. The result is illustrated in Figure A4.

The final result of the procedure, shown in Figure A4, is the complete ϵ-machine with all recurrent and transient states. The recurrent states—distributions over recurrent causal states that determine individual causal states—are the four nodes along the bottom of the figure. They are identical to those we started out with in Figure A2. The three transient states that have been constructed are the three nodes whose distributions correspond to some uncertainty about in which recurrent causal state the process is. In this way, the structure of the transient portion of an ϵ-machine shows how successive measurements refine an observer’s knowledge about in which causal state a process is.

## Appendix C. ϵ-Machine Entropy Density

We derive Equation (79), an expression for the entropy density $h_{\mu }$, in terms of the probability of the causal states and their transitions. We begin with the expression for the entropy density, Equation (36):

(A9) $$h_{\mu }=\lim_{L\to \infty }H\left[S_{L}|S_{1}\cdots S_{L-2}S_{L-1}\right]\;.$$

Using the definition of the conditional entropy, Equation (28), this may be rewritten as:

(A10) $$h_{\mu }=\lim_{L\to \infty }-\sum_{s_{L},s^{L-1}}\mathrm{Pr}\left(s_{L},s^{L-1}\right)\log_{2}\mathrm{Pr}\left(s_{L}|s^{L-1}\right),\;$$

where $s_{L}$ denotes the single spin variable at site *L* and $s^{L-1}$ denotes the block of L−1 spins from sites 1 to L−1.

The causal states $S_{\alpha }$ partition the set $\left\{s^{L-1}\right\}$; each $s^{L-1}$ belongs to one and only one causal state equivalence class. (Cf. Appendix A.) As a result we may re express the sum as follows:

(A11) $$h_{\mu }=\lim_{L\to \infty }-\;\sum_{s_{L},\alpha }\sum_{s^{L-1}\in S_{\alpha }}\;\;\;\mathrm{Pr}\left(s_{L},s^{L-1}\right)\log_{2}\mathrm{Pr}\left(s_{L}|s^{L-1}\right).$$

Causal states were defined in Equation (53) such that two blocks of spins $s_{i}^{L-1}$ and $s_{j}^{L-1}$ belong to the same causal state if and only if $\mathrm{Pr}\left(\vec{s}|s_{i}^{L-1}\right)=\mathrm{Pr}\left(\vec{s}|s_{j}^{L-1}\right),\;\mathrm{for}\;\mathrm{all}\vec{s}$. This observation enables us to perform the inner sum in Equation (A11). Each term in the argument of the logarithm is identical, since all the $s^{L-1}$’s belong to the same causal state. As a result, we can pull this term outside the sum:

(A12) $$h_{\mu }=\lim_{L\to \infty }-\;\;\sum_{s_{L},\alpha }[\log_{2}\mathrm{Pr}\left(s_{L}|S_{\alpha }\right)\left.\;\;\;\sum_{s^{L-1}\in S_{\alpha }}\;\;\;\mathrm{Pr}\left(s_{L},s^{L-1}\right)\right].$$

Note that since we are interested in the L→∞ limit, we need only concern ourselves with recurrent causal states. The inner summation has the effect of adding up the probabilities of all the $s^{L-1}$’s in the $\alpha^{\mathrm{th}}$ causal state:

(A13) $$\sum_{s^{L-1}\in S_{\alpha }}\mathrm{Pr}\left(s_{L},s^{L-1}\right)=\mathrm{Pr}\left(s_{L},S_{\alpha }\right)\;.$$

Inserting this into Equation (A12), we immediately obtain

(A14) $$h_{\mu }=-\sum_{\alpha }\sum_{s\in A}\mathrm{Pr}\left(s,S_{\alpha }\right)\log_{2}\mathrm{Pr}\left(s|S_{\alpha }\right)\;,$$

where s∈A are the spin values that can follow $S_{\alpha }$. This result is Equation (79). A little more explicitly we have

(A15) $$h_{\mu }=-\sum_{\alpha }\mathrm{Pr}\left(S_{\alpha }\right)\sum_{s\in A}\mathrm{Pr}\left(s|S_{\alpha }\right)\log_{2}\mathrm{Pr}\left(s|S_{\alpha }\right)\;,$$

where $\mathrm{Pr}(S_{\alpha })$ is the left eigenvector of the stochastic connection matrix T, normalized in probability, and the second sum is seen to be the single-spin uncertainty at each causal state.
